# Inhibition effect of pyridoxamine on lipid hydroperoxide-derived modifications to human serum albumin

**DOI:** 10.1371/journal.pone.0196050

**Published:** 2018-04-19

**Authors:** Seon Hwa Lee, Atsushi Matsunaga, Tomoyuki Oe

**Affiliations:** Department of Bio-analytical Chemistry, Graduate School of Pharmaceutical Sciences, Tohoku University, Sendai, Japan; Kermanshah University of Medical Sciences, ISLAMIC REPUBLIC OF IRAN

## Abstract

Pyridoxamine (PM) is a promising drug candidate for treating various chronic conditions/diseases in which oxidative stress and carbonyl compounds are important factors affecting pathogenicity. These abilities of PM are mainly attributed to its inhibition of advanced glycation and lipoxidation end product formation, by scavenging reactive carbonyl species. PM might therefore prevent protein damage from lipid hydroperoxide-derived aldehydes such as 4-oxo-2(*E*)-nonenal (ONE) and 4-hydroxy-2(*E*)-nonenal (HNE) by trapping them. It was previously reported that PM reacts with ONE to produce pyrrolo-1,3-oxazine (PO8) through the formation of pyrido-1,3-oxazine (PO1/PO2). In this study, we found that ONE and HNE yield an identical product containing a pyrrole ring (PO7, PH2) upon reaction with PM. The structure of PO7/PH2 was shown by LC-MS and NMR analyses to be 1-(2-hydroxy-6-hydroxymethyl-3-methylpyridin-4-ylmethyl)-2-pentylpyrrole. PO1, PO7/PH2, and PO8 were the main stable PM-ONE/HNE adducts. In the incubation of human serum albumin (HSA) with ONE or HNE, Lys residues provided the most favorable modification sites for both aldehydes, and the number of HNE-modified sites was higher than that of ONE-modified sites. When HSA was allowed to react with a linoleic acid hydroperoxide in the presence of ascorbic acid, ONE modified more residues (10 Lys, 3 His, 2 Arg) than did HNE (8 His, 2 Lys), indicating the relative reactivity of aldehydes towards amino acid residues. Upon treatment with increasing concentrations of PM, the concentrations of ONE-modified HSA peptides, but not of HNE-modified peptides, were reduced significantly and dose-dependently. Concomitantly, the formation of PM-ONE adducts increased in a dose-dependent manner. The inhibition effect of PM was also confirmed in the cell system subjected to oxidative stress. Our results demonstrate that PM can inhibit lipid hydroperoxide-derived damage to proteins by trapping ONE preferentially, and the resulting PM-ONE adducts can be used as a dosimeter for ONE production to determine the levels of lipid peroxidation.

## Introduction

Age-related degenerative diseases are associated with various oxidative chemical modifications of proteins [1]. Reactive oxygen species can directly oxidize the amino acid side chain of Met to a sulfoxide and that of Cys to sulfenic, sulfinic, or sulfonic acid [2]. Direct protein carbonylation occurs through oxidation of Lys, Arg, and Pro residues in the presence of metal ions and H_2_O_2_ [3]. Reactive oxygen species can induce the oxidation of polyunsaturated fatty acids in membrane phospholipids and other lipid-containing structures. Enzymes such as cyclooxygenases and lipoxygenases also convert polyunsaturated fatty acids to their hydroperoxides [4]. In the presence of transition metal ions or l-ascorbic acid (AscA), the lipid hydroperoxides are homolytically decomposed to highly reactive bifunctional electrophiles [5]. 4-Oxo-2(*E*)-nonenal (ONE) and 4-hydroxy-2(*E*)-nonenal (HNE), the most abundant and reactive lipid-derived aldehydes, can react with Cys, His, and Lys through Michael addition [6]. ONE also forms Schiff base adducts with the N-terminal α-amino group, Arg, and Lys [7–9]. These modifications of amino acid residues and protein crosslinking (advanced lipoxidation end products; ALEs), are known to cause altered gene regulation and protein dysfunction [6]. Glucose and other reducing sugars can modify proteins by the formation of Amadori adducts that undergo further rearrangement and oxidative decomposition to produce advanced glycation end products (AGEs) such as pentosidine and vesperlysine [10]. The reactive carbonyl species glyoxal, methylglyoxal (MGO), and glycolaldehyde (GLA) can be derived from lipid peroxidation, as well as by the autoxidation of carbohydrate and through the Schiff base intermediate of glycation reactions [11]. These reactive carbonyl species react primarily with Lys and Arg to generate AGE/ALEs such as *N*^ε^-carboxymethyl Lys (from glyoxal or GLA), *N*^ε^-carboxyethyl Lys (from MGO), and argpyrimidine (from MGO), all of which have been implicated in a number of diseases through their effects on protein structure, function, and turnover [12–14]. The accumulation of AGEs and ALEs in protein has been linked to the development of diabetic complications [10]. Therefore, the trapping of reactive carbonyl species and the inhibition of AGE/ALE formation are considered important avenues for the treatment of these diseases. Aminoguanidine (AG) was the first AGE inhibitor to demonstrate pharmacological effects in clinical trials [15]. Although AG was shown to inhibit the chemical modification of proteins during lipid peroxidation [16] and to prevent metal-catalyzed oxidation of low-density lipoprotein [17], it was suggested that AG exerts its therapeutic effects mainly through the efficient scavenging of reactive carbonyl species [18].

Pyridoxamine (PM), a vitamin B6 vitamer, functions as a coenzyme in enzymatic transaminations *in vivo* [18]. PM has recently emerged as a promising drug candidate for the treatment of diabetic complications and other chronic conditions [18]. Initially, PM was introduced as an inhibitor of AGEs formed from Amadori adducts [19] and was shown to delay the progress of renal disease in a rat model of diabetes [20]. It has also been reported that PM can inhibit the chemical modification of proteins and trap reactive intermediates derived from carbohydrates and lipids. PM prevented the modification of Lys residues and the formation of ALEs in reactions of RNase with arachidonic acid (AA) [21]. During the incubation of PM with linoleic acid (LA) and AA, several PM adducts were identified and the concentrations of some of these adducts increased in the urine of PM-treated diabetic and hyperlipidemic rats compared with control animals [21, 22]. In diabetic rats, PM lowered the levels of MGO in red blood cells and in plasma, and blocked production of the MGO-Lys dimer by forming the MGO-PM dimer [23]. PM can also readily react with GO and GLA to form a five-ring compound with a central piperazine ring (GOPM and GLAPM, respectively). In the presence of GO and GLA, PM inhibited formation of the AGE/ALE *N*^ε^-carboxymethyl Lys during incubation with bovine serum albumin [24]. Malondialdehyde (MDA), a DNA-reactive aldehyde derived from lipid peroxidation, can be trapped by PM, thereby inhibiting lipofuscin-like fluorescence induced by MDA reacting with bovine serum albumin [25]. PM is a potent scavenger of 1,4-dicarbonyls, as well as of 1,2- (GO, MGO) and 1,3-dicarbonyls (MDA). PM forms a pyrrole adduct with 4-oxopentanal [26] and a lactam adduct with 15-E_2_-isoketal (γ-ketoaldehyde) [27]. In platelets activated by AA, PM prevents the formation of Lys adducts with levuglandin, an endogenous γ-ketoaldehyde derived from the cyclooxygenase-mediated oxidation of AA [27]. Recently, pyrrolo[2,1-*b*][1,3]oxazine was identified as the major product in the reaction between PM and ONE. That the reaction of ONE with PM was more rapid than the reaction with Lys suggested the therapeutic utility of PM for scavenging ONE, which is one of the most reactive lipid-derived aldehydes [28].

In this study, we investigated the inhibition effect of PM on protein modifications induced by ONE and HNE *in vitro*. Human serum albumin (HSA), the most abundant protein in human plasma, was employed as a model protein because it contains many nucleophilic amino acid residues. Prior to investigating the inhibition effect, PM-ONE/HNE adducts and ONE/HNE-modified tryptic HSA peptides were identified following the reaction of ONE/HNE with PM or HSA, respectively, using LC/ESI-MS. HSA was then treated with ONE or HNE in the presence of PM and changes in the levels of selected PM-ONE/HNE adducts and of ONE/HNE-modified tryptic HSA peptides were monitored. The inhibition effect of PM was then confirmed by using 13(*S*)-hydroperoxy-9,11(*Z*,*E*)-octadecadienoic acid (13-HPODE) as a source of ONE and HNE, followed by using the cell system subjected to oxidative stress.

## Materials and methods

### Materials

ONE, HNE, and 13-HPODE were purchased from Cayman Chemical Co. (Ann Arbor, MI). PM dihydrochloride was purchased from Fluka Analytical (Buchs, Switzerland). Human angiotensin (Ang) II (DRVYIHPF) was obtained from Peptide Institute, Inc. (Osaka, Japan). HPLC-grade acetonitrile (ACN), urea, sodium hydroxide, disodium hydrogenphosphate 12·H_2_O, sodium dihydrogenphosphate dihydrate, ammonium bicarbonate, dithiothreitol, iodoacetamide, ethanol, hexane, diethyl ether, formic acid (FA), CD_3_OD, and Dulbecco's phosphate buffered saline (D-PBS) were purchased from Nacalai Tesque, Inc. (Kyoto, Japan). HSA (lyophilized powder, fatty acid free, globulin free, ≥ 99% [agarose gel electrophoresis]) and AscA were purchased from Sigma-Aldrich (St. Louis, MO). Sequencing-grade modified trypsin was purchased from Promega (Madison, WI). Dulbecco's modified Eagle's medium (DMEM) and copper (II) sulfate pentahydrate (CuSO_4_·5H_2_O) were obtained from Wako Pure Chemical Industries, Ltd. (Osaka, Japan). Fetal bovine serum (FBS) was purchased from Biological Industries Israel Beit-Haemek Ltd. (Kibbutz Beit-Haemek, Israel). Chelex-100 chelating ion-exchange resin (100–200 mesh size) was purchased from Bio-Rad Laboratories (Hercules, CA). Amicon Ultra centrifugal filters (0.5 mL, 30 K) were purchased from Millipore (Billerica, MA). Ultrapure water was obtained from a Milli-Q Integral 10 (EMD Millipore) equipped with a 0.22 μm membrane cartridge.

### Liquid chromatography

Chromatography for LC systems 1 and 2 was carried out on a Surveyor MS pump (separation module) equipped with a Surveyor AS autosampler, column oven, and PDA detector (Thermo Fisher Scientific Inc., Waltham, MA). Chromatography for LC system 3 was carried out on an Agilent 1100 LC system (Agilent Technologies, Inc., Santa Clara, CA) equipped with a G1312A bin pump, G1329A ALS autosampler, G1379A degasser, and UV detector at ambient temperature. Chromatography for LC systems 4 and 5 was carried out on Ultimate 3000 LC system (Thermo Fisher Scientific Inc.) equipped with an SRD-3600 degasser, DGP-3600MB pump, FLM-3100B (nano, 2X2P-10P) flow manager, and WPS-3000TBPL (nano, CAP) autosampler. Chromatography for LC system 6 was carried out on an Ultimate 3000 LC system (Thermo Fisher Scientific Inc.) equipped with an SRD-3600 degasser, HPG-3400 RS pump, TCC-3000SD column compartment, and WPS-3000TRS autosampler. LC systems 1, 2, and 4–6 employed a Jupiter C18 column (150 × 2.0 mm i.d., 5 μm, 300 Å; Phenomenex, Torrance, CA). LC system 3 employed an Inertsil ODS-2 column (250 × 6.0 mm i.d., 5 μm, 150 Å; GL Science, Shinjuku, Tokyo, Japan). For LC system 1, solvent A was water/ACN (98:2, v/v) containing 0.1% (v/v) FA, and solvent B was ACN/water (98:2, v/v) containing 0.1% (v/v) FA. For LC system 2–6, solvent A was water containing 0.1% (v/v) FA, and solvent B was ACN containing 0.1% (v/v) FA. The linear gradient for LC system 1 was as follows; 27% B at 0 min, 30% B at 4 min, 70% B at 30 min, 90% B at 31 min, 90% B at 34 min, 27% B at 35 min, 27% B at 50 min. The linear gradient for LC system 2 and 4 was as follows; 0% B at 0 min, 60% B at 60 min, 90% B at 60.01 min, 90% B at 70 min, 0% B at 70.01 min, 0% B at 90 min. The linear gradient for LC system 3 was as follows; 20% B at 0 min, 35% B at 15 min, 95% B at 16 min, 95% B at 19 min, 20% B at 20 min, 20% B at 35 min. The linear gradient for LC system 5 was as follows; 0% B at 0 min, 50% B at 80 min, 90% B at 80.01 min, 90% B at 90 min, 0% B at 90.01 min, 0% B at 110 min. The linear gradient for LC system 6 was as follows; 0% B at 0 min, 80% B at 40 min, 95% B at 40.1 min, 95% B at 45 min, 0% B at 45.1 min, 0% B at 60 min. The separation using LC system 1 was performed with a flow rate of 0.2 mL/min at 0–35 min, 0.25 mL/min at 35–46 min, and 0.2 mL/min at 46–50 min and column oven temperature of 25 °C. The separation using LC system 3 was performed with a flow rate of 1.0 mL/min and column oven temperature of 25 °C. The separation using LC systems 2 and 4–6 was performed with a flow rate of 0.2 mL/min and column oven temperature of 40 °C.

### Mass spectrometry

The LCQ-DECA ion trap mass spectrometer (Thermo Fisher Scientific Inc.) equipped with an ESI source was used in positive ion mode for LC system 1 and 2. Data was processed using an Xcalibur (version 2.0 SR2, Thermo Fisher Scientific Inc.). The operating conditions were as follows: heated capillary temperature, 300 °C; ion spray voltage, 4.5 kV; sheath and auxiliary gas (nitrogen) pressures, 90 and 15 (arbitrary units), respectively. The parameters for ion trap MS were as follows: full scan range to obtain precursor ions, *m/z* 300–2000; isolation width, 2.0. Helium was used as the collision gas in collision-induced dissociation experiments coupled with MS/MS. The relative collision energy was set at 35%.

The LTQ Orbitrap Velos hybrid ion trap-orbitrap mass spectrometer (Thermo Fisher Scientific Inc.) equipped with an ESI source was used in the positive ion mode for LC systems 4 and 5. Data were processed using Xcalibur (version 2.1.0). The operating conditions were as follows: heated capillary, 200 °C; spray voltage, 4.5 kV; resolution 60,000; sheath and auxiliary gas (nitrogen) pressures, 50 and 15 (arbitrary units), respectively. Full scanning analyses were performed in the range of *m/z* 300–2000. Helium was used as the collision gas in collision-induced dissociation experiments coupled with MS/MS. The relative collision energy was set at 35%.

The TSQ-Vantage triple quadrupole mass spectrometer (Thermo Fisher Scientific Inc.) equipped with an ESI source was used in positive ion mode for LC systems 6. Data was processed using Xcalibur (version 2.1.0, Thermo Fisher Scientific Inc.). Argon was used as the collision gas in CID experiments coupled with MS/MS at 1.6 mTorr in the second (rf-only) quadrupole. For the selected reaction monitoring (SRM) analysis, the transition, collision energy, and *S*-lens RF amplitude were optimized. The operating conditions were as follows: heated capillary temperature, 220 °C; ion spray voltage, 3.0 kV; vaporizer temperature, 450 °C; sheath and auxiliary gas (nitrogen) pressures, 50 and 15 (arbitrary units), respectively. The scan width was *m/z* 1.00 and the scan time was 0.05 s per SRM transition.

### Database search

Peptide sequences and modifications were identified using Proteome Discoverer 1.3 (Thermo Fisher Scientific Inc.). The peak list was searched by Sequest (University of Washington, Washington) against UniprotKB/Swiss-Prot (2014_11, 547085 sequences). Search settings were as follows: taxonomy, *Homo sapiens*; enzyme, trypsin; maximum missed cleavage, 2; static modification, carbamidomethylation (Cys); dynamic modification, oxidation (Met); precursor mass tolerance, 3 ppm; fragment mass tolerance, 0.6 Da; target false discovery rate (FDR), 0.01. ONE (Cys/His/Lys), [ONE–H_2_O] (Cys/His/Lys/Arg), HNE (Cys/His/Lys), [HNE–H_2_O] (Cys/His/Lys), and [HNE– 2H_2_O] (Lys) were added to the dynamic modification list.

### NMR

The NMR spectrum was recorded on a JNM-AL400 spectrometer (JEOL Resonance Inc., Tokyo, Japan) at room temperature. The sample of PH2 [PM + HNE − 2H_2_O] was dissolved in 800 μL of CD_3_OD. Chemical sifts were given on the δ scale (ppm) by assigning the residual solvent peak for methanol (δ 3.31) as internal reference.

### Preparation of PH2 [PM + HNE − 2H_2_O] for NMR analysis

A solution of HNE (4.7 mg, 30 μmol) in 10 mL of ethanol was added to PM dihydrochloride (12.1 mg, 50 μmol) in 50 mM Chelex-treated sodium phosphate buffer (pH 7.4, 90 mL). The reaction mixture was incubated for 24 h at 37 °C, followed by the mixture was heated for 24 h at 80 °C to promote the dehydration of PH1 [PM + HNE]. After the reaction was complete, the solution was washed with hexane (100 mL × 3), followed by extraction of PH2 with diethyl ether (100 mL × 3). The ether layer was evaporated and reconstituted with 1 mL of 10% ethanol in water and then purified by LC-UV using LC system 3. PH2 was obtained as a yellow solid (0.42 mg isolated).

### Reaction of PM with ONE or HNE

A solution of ONE (4.63 μg, 30 nmol) or HNE (4.69 μg, 30 nmol) in 10 μL of ethanol was added to PM dihydrochloride (12.1 μg, 50 nmol) in 50 mM Chelex-treated sodium phosphate buffer (pH 7.4, 90 μL). The reaction mixture of ONE and HNE was incubated at 37 °C for 24 h. A portion of the reaction mixture (10 μL) was analyzed by LC/ESI-MS and MS/MS using LC system 1 for PM-ONE adducts and LC system 2 for PM-HNE adducts.

### Reaction of HSA with ONE or HNE in the presence of PM

A solution of ONE (18.5 μg, 120 nmol) or HNE (18.7 μg, 120 nmol) in 20 μL of ethanol was added to HSA (740.0 μg, 11.2 nmol) in 50 mM Chelex-treated sodium phosphate buffer (pH 7.4, 370 μL). The reaction mixtures were treated with PM dihydrochloride (0−48.2 μg, 0−200 nmol) in 50 mM Chelex-treated sodium phosphate buffer (pH 7.4, 10 μL) and incubated at 37 °C for 24 h. After incubation, solutions were subsequently transferred into a filter device (cutoff 30 kDa) and the modified HSA was separated from PM-ONE/HNE adducts by centrifugation at 11,300 × g for 12 min. The ultrafiltrate containing PM-ONE/HNE adducts was analyzed by LC/ESI-MS and MS/MS using LC system 4. In the case of HNE-modified HSA, the residue on the filter was treated with 100 μL of 0.1 M NaBH_4_ in 0.1 M sodium hydroxide. The reaction mixture was incubated for 1 h at 37 °C. After incubation, the excess reagent was removed by centrifugation using a filter device. The resulting ONE- or HNE-modified HSA were then washed with 380 μL of 6.5 M urea by centrifugation at 11,300 × g for 12 min. The solution of modified HSA (ca. 20 μL) on the filter was adjusted to approximately 2 μg/μL with 6.5 M urea (380 μL) for tryptic digestion.

### Reaction of HSA with lower concentrations of ONE or HNE in the presence of PM

A solution of ONE or HNE (1.2 or 12.0 nmol) in 20 μL of ethanol was added to HSA (740.0 μg, 11.2 nmol) in 50 mM Chelex-treated sodium phosphate buffer (pH 7.4, 370 μL). The reaction mixtures were treated with PM dihydrochloride (0−20 nmol) in 50 mM Chelex-treated sodium phosphate buffer (pH 7.4, 10 μL) and incubated at 37 °C for 24 h. After incubation, the samples were prepared for trypsin digestion as described above.

### Reaction of HSA with 13-HPODE in the presence of AscA and PM

A solution of 13-HPODE (93.8 μg, 300 nmol) in 10 μL of ethanol was added to HSA (180.0 μg, 2.72 nmol) in 50 mM Chelex-treated sodium phosphate buffer (pH 7.4). The solution was treated with PM dihydrochloride (0−12.1 μg, 0−50 nmol). The reaction was initiated by adding AscA (17.6 μg, 100 nmol) and continued for 24 h at 37 °C. The total reaction volume was maintained at 100 μL. The resulting modified HSA and PM adducts were then separated as described above. PM adducts was analyzed by LC/ESI-MS and MS/MS using LC system 4. The modified HSA (without reduction) were washed with 380 μL of 6.5 M urea by centrifugation at 11,300 g for 12 min. The solution of modified HSA (ca. 20 μL) on the filter was adjusted to approximately 2 μg/μL with 6.5 M urea (80 μL) for tryptic digestion.

### Reduction, alkylation, and digestion of modified HSA

Sequencing-grade modified trypsin was stored until use at −20 °C as 0.1 mg/mL solutions in storage buffer. An aliquot (50 μL) of the modified HSA (2 μg/μL, in 6.5 M urea) was diluted with ammonium bicarbonate (12.5 mM, 50 μL). The solution was incubated with dithiothreitol (110 mM, 10 μL, in 12.5 mM ammonium bicarbonate) at 37 °C for 1 h to reduce the disulfide bonds. The reduced samples were incubated in the dark at 37 °C for 45 min with iodoacetamide (600 mM, 10 μL, in 12.5 mM ammonium bicarbonate) to alkylate the sulfhydryl groups of Cys. The reduced and alkylated samples were diluted with ammonium bicarbonate (12.5 mM, 370 μL) and incubated with sequencing-grade modified trypsin (0.1 mg/mL, 10 μL) overnight at 37 °C. Portions of the tryptic samples (10 μL) were analyzed by LC/ESI-MS and MS/MS using LC system 5.

### Cell culture

Rat L6 skeletal muscle cells were obtained from the American Type Culture Collection (Rockville, MD). Cells were plated onto appropriate culture dishes and cultured in DMEM containing 10% (v/v) FBS. Cultures were maintained at 37 °C in a humidified atmosphere containing 5% (v/v) CO_2_. The medium was replaced every other day. All experiments were performed after L6 cells were reached 80% confluence.

### Cell treatment

Culture medium was removed and replaced with serum-free medium. The cells were then incubated with the indicated doses of PM for 3 h. After incubation, cells were treated with Ang II (50 μM) in the presence of AscA (1 mM) and CuSO_4_ (50 μM), and incubated at 37 °C for 24 h.

### Extraction of PM adducts from the cell culture medium

L6 cells were reseeded in 12-well plates for the PM adducts extraction. After the treatment described above, the culture medium was collected and the unreacted aldehydes were removed with hexane (200 μL × 3), followed by extraction of PM adducts with diethyl ether (200 μL × 3). The ether layer was evaporated and reconstituted with 40 μL of H_2_O. A portion of sample (10 μL) was analyzed by LC/ESI-SRM/MS using LC system 6.

## Results

### LC/ESI-MS analysis of the reaction between PM and ONE

**Fig 1:**
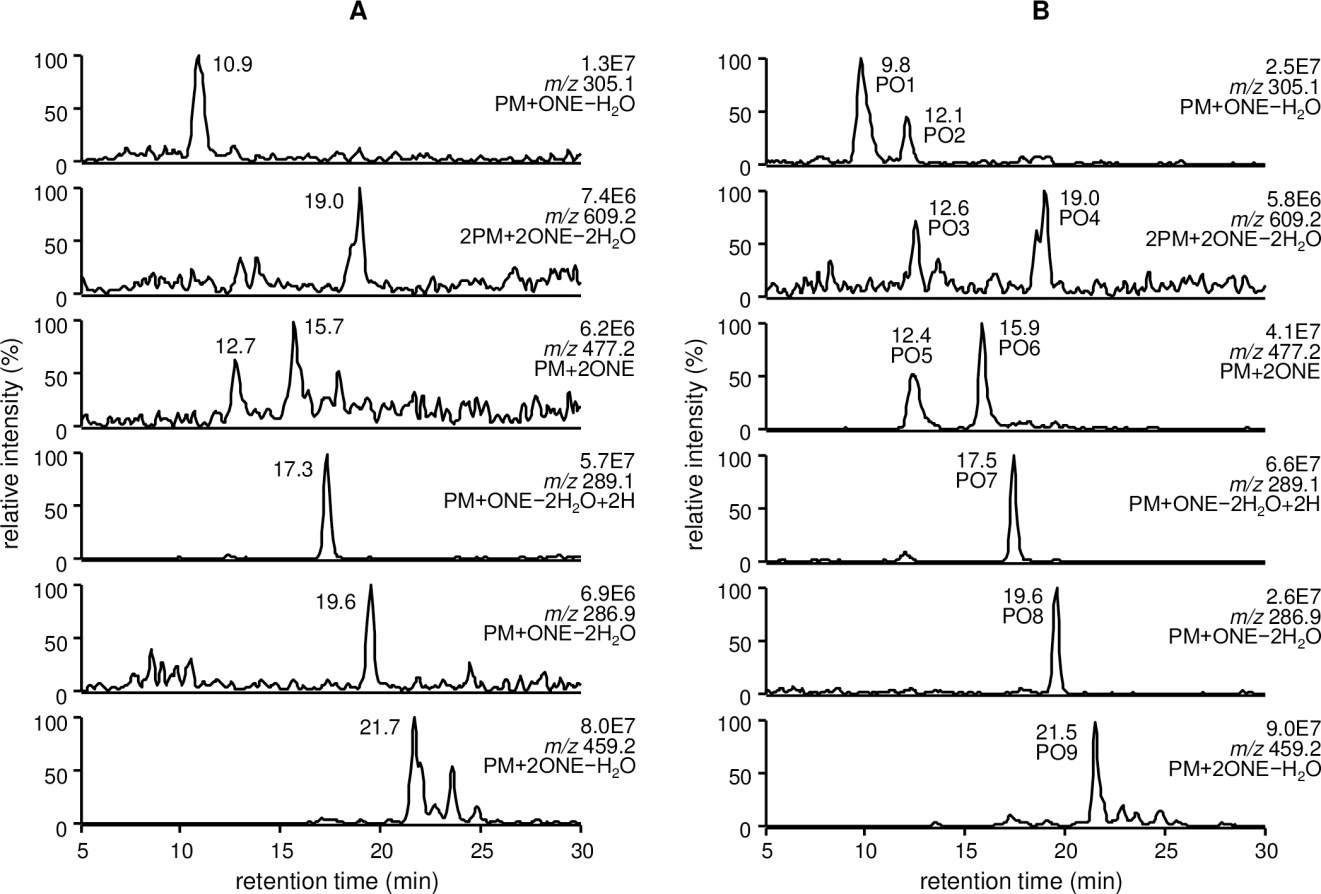
LC/ESI-MS analysis of the reaction between PM and ONE at 37 °C for (A) 24 h and (B) 5 days.

The formation of several products was observed after 24 h incubation (Fig 1A) of PM and ONE. When the incubation was continued for 5 days, 9 adducts were detected (Fig 1B). The most polar PM-ONE adducts (PO1 and PO2) eluted at 9.8 and 12.1 min, respectively, and had an identical [M + H]^+^ at *m/z* 305.1. This product mass corresponds to a 1:1 reaction of PM with ONE ([M + H]^+^; *m/z* 323.3) followed by the loss of water (− 18 Da). The products that eluted at 12.6 min (PO3) and 19.0 min (PO4) also had an identical [M + H]^+^ at *m/z* 609.2, corresponding to the reaction of two molecules of PM with two molecules of ONE ([M + H]^+^; *m/z* 645.6) and the loss of two molecules of water (− 36 Da). Similarly, the products that eluted at 12.4 min (PO5) and 15.9 min (PO6) had an identical [M + H]^+^ at *m/z* 477.2, indicating the reaction of one molecule of PM with two molecules of ONE. The products that eluted at 17.5 min (PO7) and 19.6 min (PO8) showed an [M + H]^+^ at *m/z* 289.1 and 286.9, respectively, and arose from a 1:1 reaction of PM with ONE ([M + H]^+^; *m/z* 323.3), followed by the loss of 34 Da (− 2H_2_O + 2H) and 36 Da (− 2H_2_O), respectively. The last eluting PM-ONE product had a retention time of 21.5 min (PO9). It showed an [M + H]^+^ at *m/z* 459.2, corresponding to the reaction of one molecule of PM with two molecules of ONE ([M + H]^+^; *m/z* 477.5) and the loss of water (− 18 Da).

### LC/ESI-MS/MS analysis of the major ONE-modified PM

**Fig 2:**
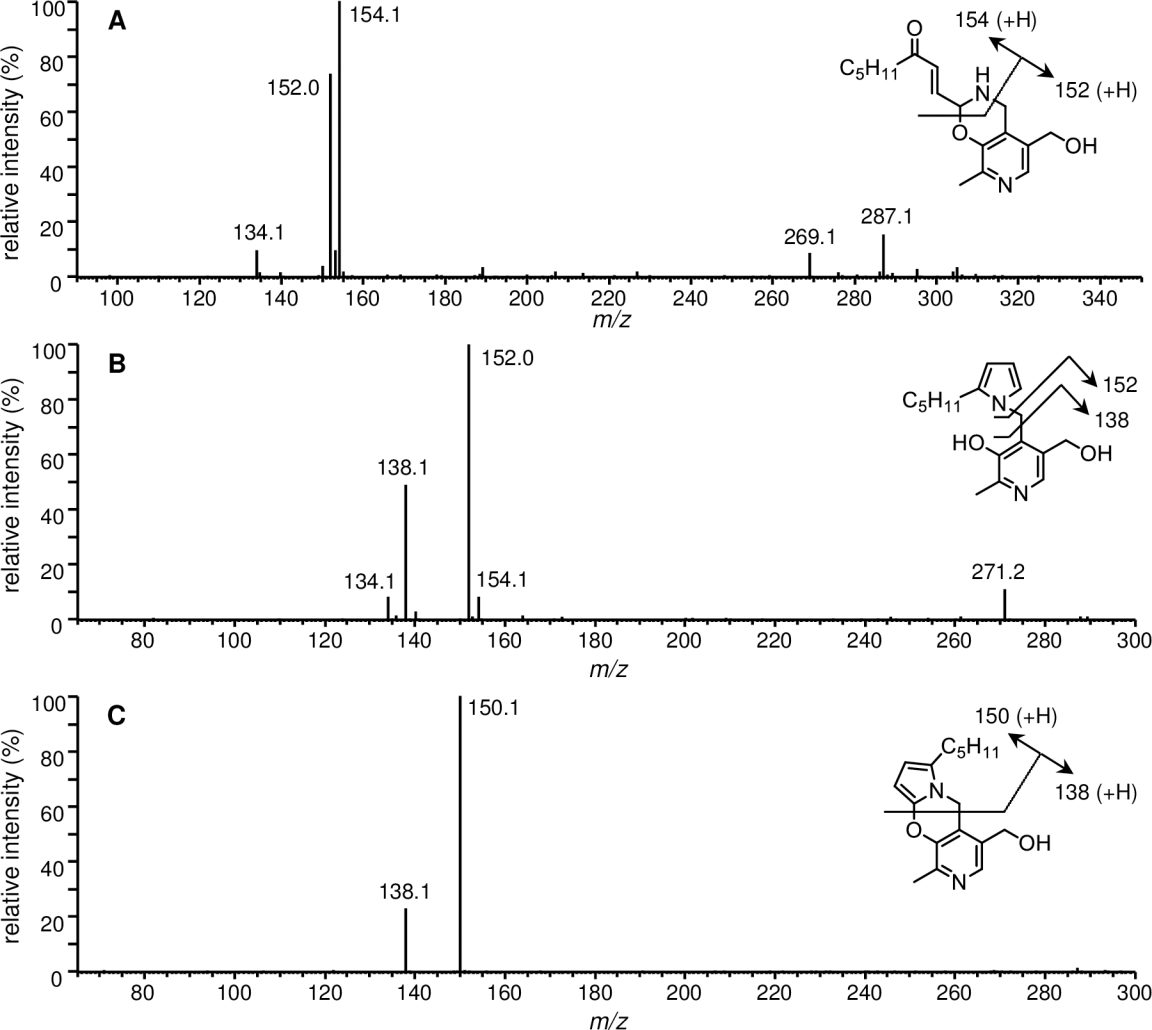
LC/ESI-MS/MS analysis of PM-ONE adducts (A) PO1 (*m/z* 305.1), (B) PO7 (*m/z* 289.1) and (C) PO8 (*m/z* 286.9).

LC/ESI-MS/MS analysis was performed for the major products (PO1, PO7, and PO8) of the reaction between PM and ONE. The MS/MS spectrum of PO1 exhibited two intense product ions, at *m/z* 154.1 and 152.0 (Fig 2A). The MS/MS spectra of PO7 and PO8 show similar sets of product ions, with the major product ions being *m/z* 152.0 and 138.1 for PO7 (Fig 2B) and *m/z* 150.1 and 138.1 for PO8 (Fig 2C). Amarnath et al. previously demonstrated that 2-aminomethylphenols and ONE form pyrido-1,3-oxazines (corresponding to PO1/PO2), followed by dehydration to the multicyclic pyrrole, pyrrolo[2,1-*b*][1,3]oxazines (corresponding to PO8) [28]. The obtained product ions of PO1 and PO8 were consistent with the reported structures.

### LC/ESI-MS analysis of the reaction between PM and HNE

**Fig 3:**
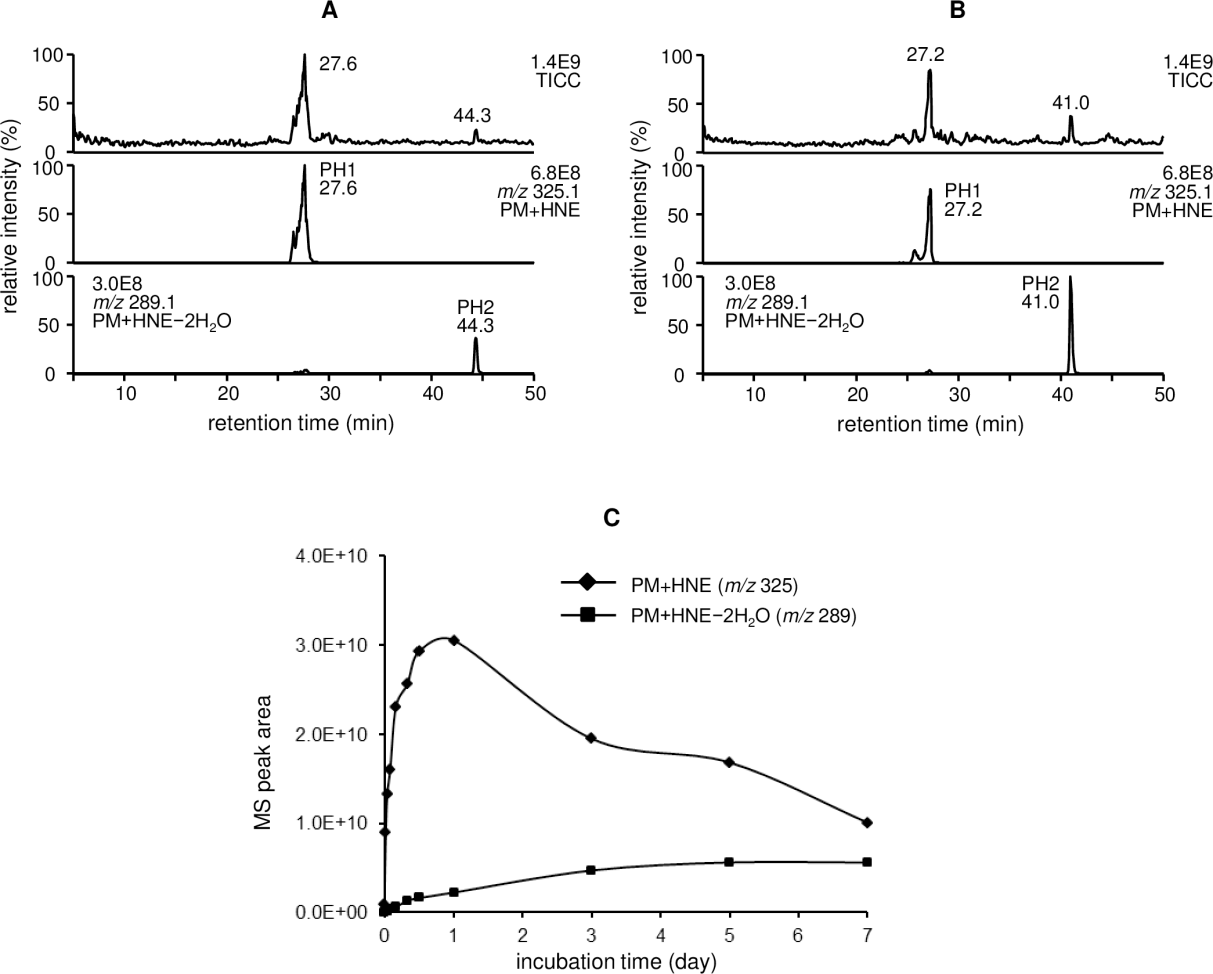
LC/ESI-MS analysis of the reaction between PM and HNE at 37 °C for (A) 24 h and (B) 5 days. (C) Formation of PM-HNE adducts in 7 days.

LC-ESI/MS analysis of the products from the reaction between PM and HNE at 37 °C for 24 h revealed the presence of two major products with [M + H]^+^ at *m/z* 325.1 (*t*_R_ = 27.6 min, PH1) and 289.1 (*t*_R_ = 44.3 min, PH2), corresponding to a 1:1 reaction of PM with HNE ([M + H]^+^, *m/z* 325.3) followed by the loss of 36 Da (− 2H_2_O) (Fig 3A). When the incubation was continued for 5 days, a decrease in PH1 and a concomitant increase in PH2 was observed (Fig 3B). Time course experiments were performed to gain further understanding of the formation of PM-HNE adducts. The reaction between PM and HNE in phosphate buffer (pH 7.4) was monitored by LC-ESI/MS for 7 days (Fig 3C). PH1 increased quickly (0–24 h) and then decreased (1–7 days), while PH2 increased to its maximum level at 5 days, suggesting dehydration of PH1 to PH2.

### LC/ESI-MS/MS analysis of the major HNE-modified PM

**Fig 4:**
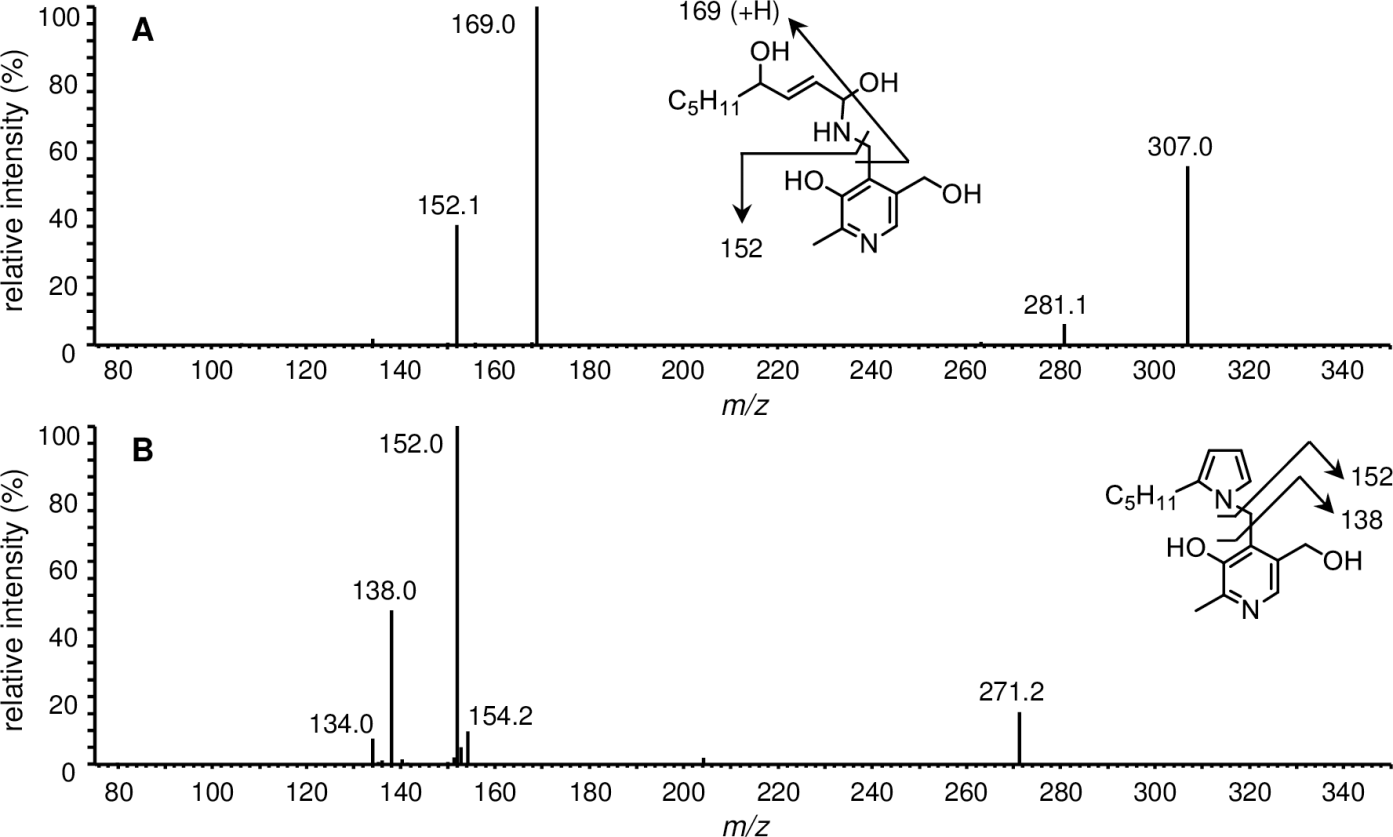
LC/ESI-MS/MS analysis of PM-HNE adducts (A) PH1 (*m/z* 325.1) and (B) PH2 (*m/z* 289.1).

LC-ESI-MS/MS analysis of the major products (PH1 and PH2) of the reaction between PM and HNE was performed. The MS/MS spectrum of PH1 revealed two characteristic product ions, at *m/z* 169.0 and 152.1 (Fig 4A). These ions were consistent with the nucleophilic addition of the PM amino group to the C-1 aldehyde of HNE. The [M + H]^+^ of PH2 corresponded to PM + HNE − 2H_2_O, suggesting that PH2 was produced via Schiff base formation followed by intramolecular cyclization and dehydration to the pyrrole. The major product ions of PH2, at *m/z* 152.0 and 138.0, indicated that the loss of two water molecules occurred at the HNE side (Fig 4B). The LC/ESI-MS and MS/MS properties of PH2 were identical to those of PO7 (PM + ONE − 2H_2_O + 2H, Fig 2B).

### NMR Analysis of PH2 (PO7)

**Fig 5:**
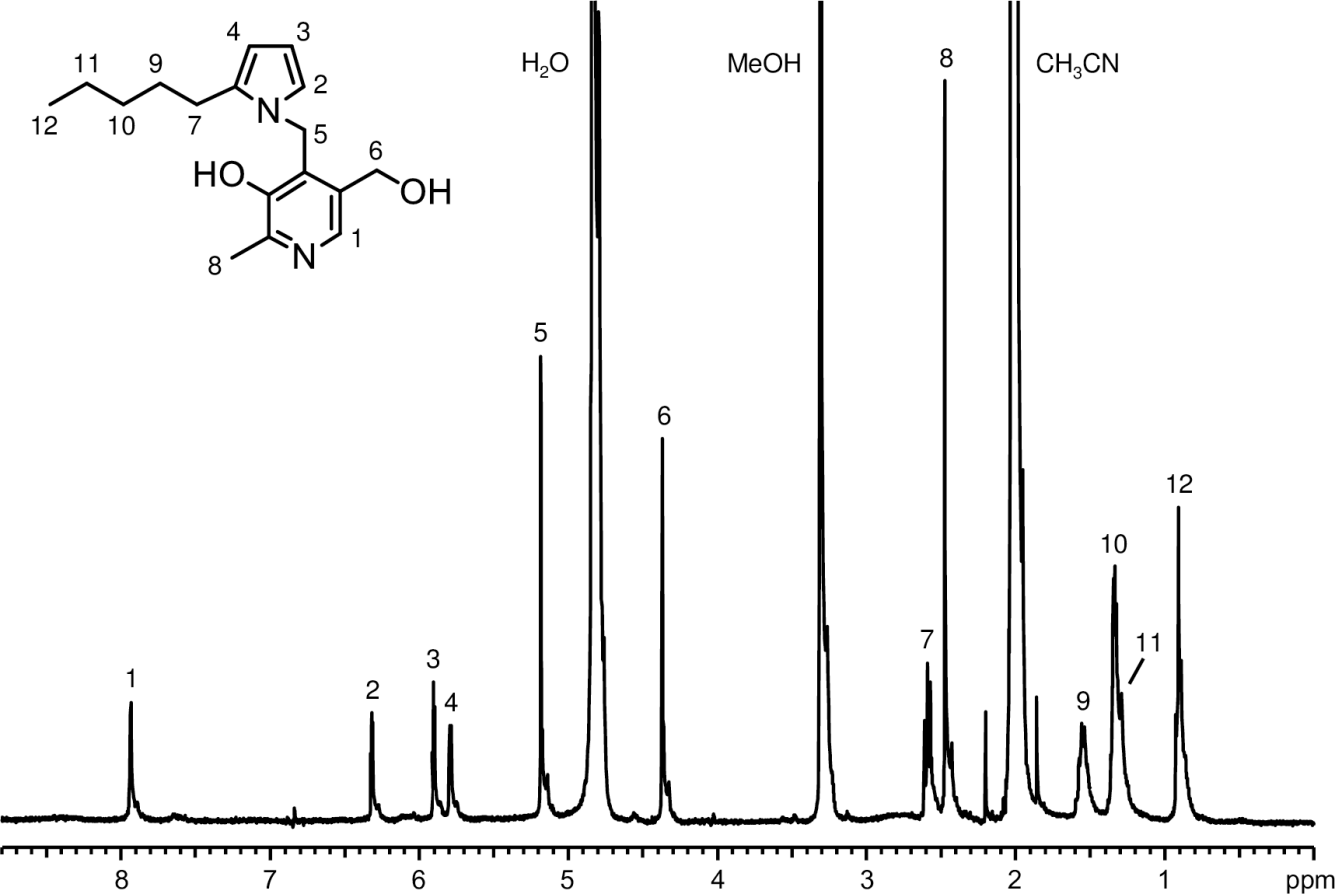
^1^H NMR spectrum of PH2 (PO2) in CD_3_OD.

**Fig 6:**
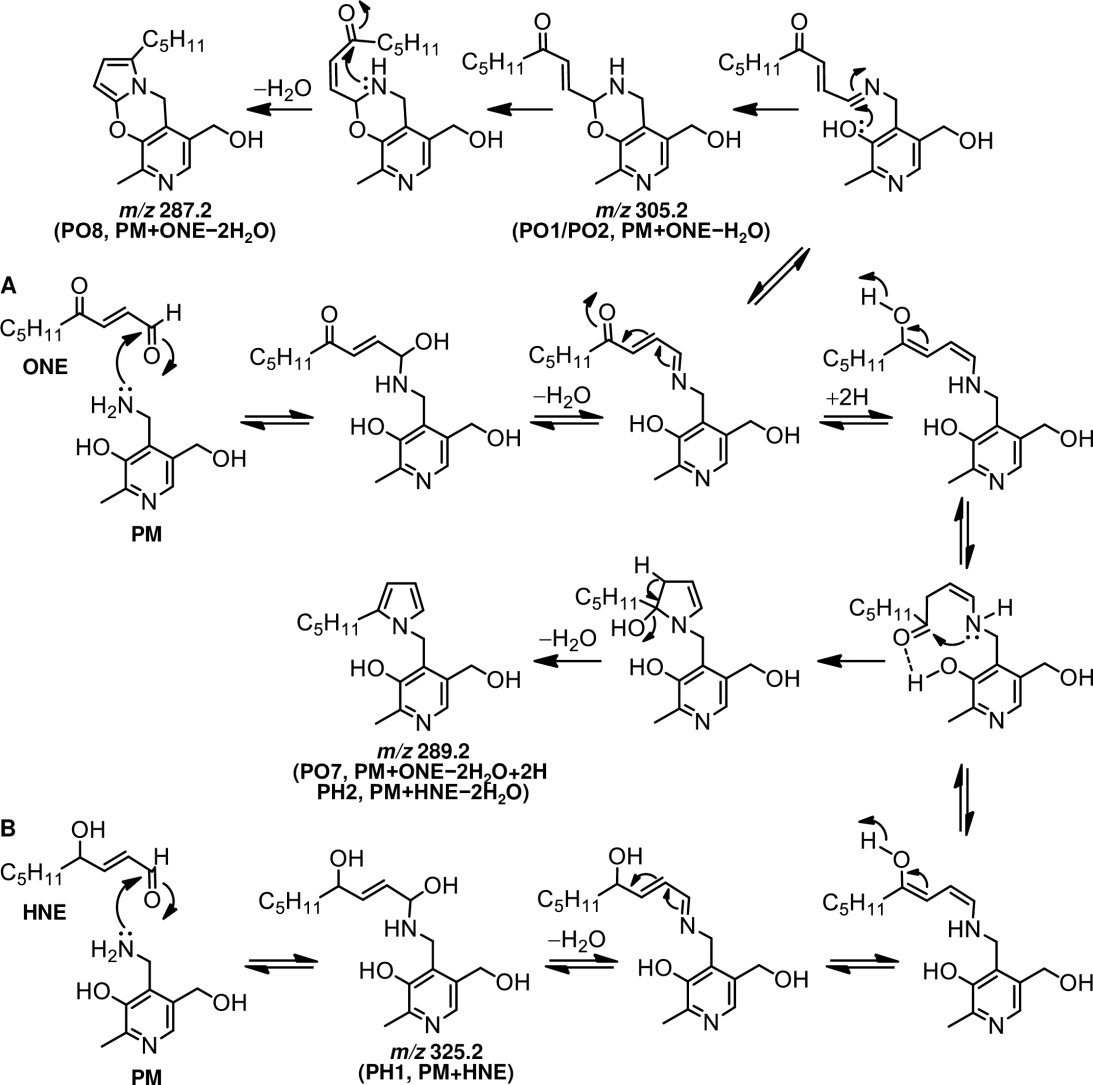
Proposed mechanism for the formation of (A) PM-ONE and (B) PM-HNE adducts from the reaction of PM with ONE or HNE, respectively.

^1^H NMR analysis revealed the presence of a pyrrole ring (Fig 5). The chemical shifts of aromatic protons at 5.79–6.32 ppm suggested that these protons (H-2, -3, and -4) were on a pyrrole ring. The *J* coupling of H-2 and -3 was consistent with that of pyrrole hydrogens at the 2- and 3-position, respectively. Proton assignments were as follows: (400 MHz, CD_3_OD) δ 0.91 (H-12, t, 3H, *J* = 7.1 Hz), 1.29 (H-11, m, 2H), 1.33 (H-10, m, 2H), 1.56 (H-9, m, 2H), 2.48 (H-8, s, 3H), 2.59 (H-7, t, 2H, *J* = 7.7 Hz), 4.37 (H-6, s, 2H), 5.19 (H-5, s, 2H), 5.79 (H-4, m, 1H), 5.90 (H-3, dd, 1H, *J* = 2.9, 3.4 Hz), 6.32 (H-2, dd, 1H, *J* = 2.9, 1.9 Hz), 7.93 (H-1, s, 1H), (OH not observed). The NMR and LC-MS (Figs 2 and 4) data were consistent with the structure of 1-(2-hydroxy-6-hydroxymethyl-3-methylpyridin-4-ylmethyl)-2-pentylpyrrole (PO7/PH2) (Fig 6).

### LC/ESI-MS analysis of the reaction between HSA and ONE

The total ion current chromatogram (TICC) of the MS (S1A Fig) showed a large number of peptide peaks. Database analyses revealed the presence of 57 ONE-modified peptides (S1 Table). Most of the modifications occurred on Lys residues (31 sites), resulting in a mass increase of 154 Da (+ ONE) or 136 Da (+ [ONE − H_2_O]). Arg residues (9 sites) were the next most favorable modification site, with a mass increase of 136 Da (+ [ONE − H_2_O]). Modifications at His residues (4 sites) were also detected. When decreased concentration of ONE (30 and 3 μM) was incubated with HSA, the modification sites were also reduced to 19 and 7, respectively (S1 Table).

### LC/ESI-MS analysis of the reaction between HSA and HNE

Database analyses for the reaction between HSA and HNE (S1B Fig) revealed the presence of 94 HNE-modified peptides (S2 Table). Most of the modifications occurred on Lys residues (44 sites), resulting in a mass increase of 158 Da (+ HNE + 2H), 140 Da (+ [HNE − H_2_O] + 2H), or 120 Da (+ [HNE − 2H_2_O], pyrrole formation). His residues (15 sites) were the next most favorable modification site, with a mass increase of 158 Da (+ HNE + 2H) or 140 Da (+ [HNE − H_2_O] + 2H). The incubation of decreased concentration of HNE (30 and 3 μM) with HSA resulted in reduction of the modification sites to 14 and 4, respectively (S2 Table).

### LC/ESI-MS analysis of the reaction between HSA and ONE in the presence of PM

Changes in the levels of PM-ONE adducts and ONE-modified HSA peptides were monitored in the reactions between HSA and ONE in the presence of increasing concentrations of PM. Prior to analysis of the ONE-modified HSA peptides, preliminary experiments were carried out to determine an appropriate internal standard (IS) and to select target modified peptides. An IS was employed to normalize the MS peak intensities of modified peptides in each sample. A^175^ACLLPK^181^ was chosen as the IS because it was consistently detected in all samples examined and contains no amino acid residues that can be modified by ONE. In addition, seven target peptides were selected based on the frequency of their detection and MS intensity.

LC/ESI-MS analyses revealed the presence of four PM-ONE adducts (S2 Fig). The products eluting at 26.3 and 29.4 min showed an [M + H]^+^ at *m/z* 305, corresponding to PO1 and PO2, respectively. The other adducts, which eluted at 36.3 min ([M + H]^+^; *m/z* 289) and 38.9 min ([M + H]^+^; *m/z* 287), corresponded to PO7 and PO8, respectively. The MS peak intensities of all PM-ONE adducts increased in a dose-dependent manner, and PO1 was the most intense (S3A Fig). S4 Fig shows the extracted ion chromatogram of seven selected ONE-modified HSA peptides, together with the IS from the reaction without PM: A^175^ACLLPK^181^ (IS, *t*_R_ = 25.4 min), S^65^LHTLFGDK*LCTVATLR^81^ (HO1, 59.0 min, * = ONE − H_2_O), K*^137^YLYEIAR^144^ (HO2, 47.2 min, * = ONE − H_2_O), Y^161^K*AAFTECCQAADK^174^ (HO3, 41.1 min, * = ONE − H_2_O), L^182^DELRDEGK*ASSAK^195^ (HO4, 35.1 min, * = ONE − H_2_O), A^210^FK*AWAVAR^218^ (HO5, 49.5 min, * = ONE − H_2_O), N^429^LGK*VGSK^436^ (HO6, 37.6 min, * = ONE), and K*^525^QTALVELVK^534^ (HO7, 50.2 min, * = ONE − H_2_O). These peptides were detected in all samples examined. The normalized MS peak intensities of seven ONE-modified peptides decreased in a dose-dependent manner upon treatment with PM (S5A Fig); for example, PM (500 μM) inhibited their formation by approximately 30–75%. This suggested that PM can protect Lys residues from ONE-derived modification. When HSA was incubated with physiologically relevant concentrations of ONE (3 μM) and PM (0.5–50 μM), dose-dependent increases in PM-ONE adducts (S3B Fig) and decreases in ONE-modified HSA peptides (S5B Fig) were also observed. However, some of adducts/modifications were below the detection limit of current LC-MS method.

### LC/ESI-MS analysis of the reaction between HSA and HNE in the presence of PM

Changes in the levels of PM-HNE adducts and in HNE-modified HSA peptides were also monitored in reactions between HSA and HNE in the presence of increasing concentrations of PM. The reaction scale, work-up procedure, and LC-MS system for analyzing the PM-HNE adducts were the same as used for analyzing the reaction with ONE. LC/ESI-MS analyses revealed the presence of two PM-HNE adducts (S6 Fig). The products eluting at 17.6 min ([M + H]^+^; *m/z* 325.1) and 36.0 min ([M + H]^+^; *m/z* 289.1) corresponded to PH1 and PH2, respectively. The MS peak intensities of both PM-HNE adducts increased in a dose-dependent manner, and PH2 was the most intense (S7A Fig). HNE-modified HSA proteins were reduced after separation of the PM-HNE adducts, followed by alkylation, digestion with trypsin, and LC/ESI-MS and MS/MS analysis using LC system 5. The tryptic HSA peptide A^175^ACLLPK^181^ was used as an IS for the normalization of six selected HNE-modified HSA peptides (S8 Fig): A^175^ACLLPK^181^ (IS, 24.8 min), S^65^LH*TLFGDK^73^ (HH1, 44.0 min, * = HNE), H*^146^PYFYAPELLFFAK^159^ (HH2, 57.4 min, * = HNE), L^182^DELRDEGK*ASSAK^195^ (HH3, 46.5 min, * = HNE − 2H_2_O), V^241^H*TECCHGDLLECADDRADLAK^262^ (HH4, 32.8 min, * = HNE), S^247^H*CIAEVENDEMPADLPSLAADFVESK^273^ (HH5, 52.0 min, * = HNE), and E^501^FNAETFTFH*ADICTLSEK^519^ (HH6, 49.1 min, * = HNE). These peptides were detected in all samples examined. In contrast to the ONE-modified HSA peptides, not all HNE-modified peptides decreased in intensity in response to increasing PM concentration (S9A Fig). Only HH3, containing a HNE-pyrrole modification at K190, exhibited a clear decrease in a dose-dependent manner, with 500 μM PM inhibiting HH3 formation by approximately 40%. This suggested that PM may protect proteins from HNE-pyrrole adduction at Lys residues but not from Michael addition at His residues. When HSA was incubated with physiologically relevant concentrations of HNE (3 μM) and PM (0.5–50 μM), PM-HNE adduct PH2 (S7B Fig) increased in a dose-dependent manner. As for HNE-modified peptides, a dose-dependent reduction was observed only for HH3 (S9B Fig), supporting the results obtained from the experiments with higher concentrations of HNE and PM above.

### LC/ESI-MS analysis of the reaction between HSA and 13-HPODE in the presence of AscA and PM

**Fig 7:**
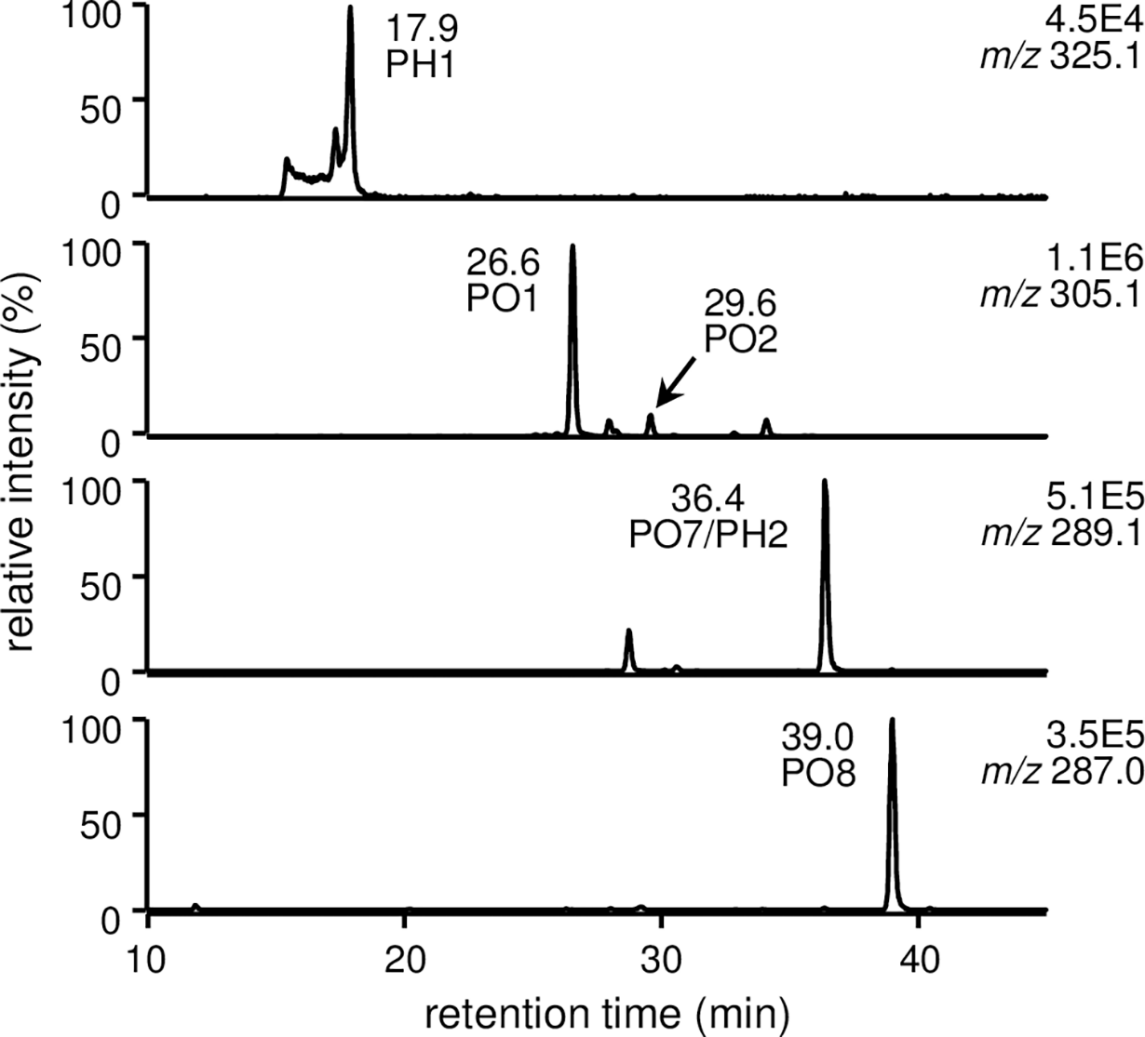
LC/ESI-MS analysis of PM-ONE/HNE adducts in the reaction between HSA and 13-HPODE in the presence of AscA and PM.

**Fig 8:**
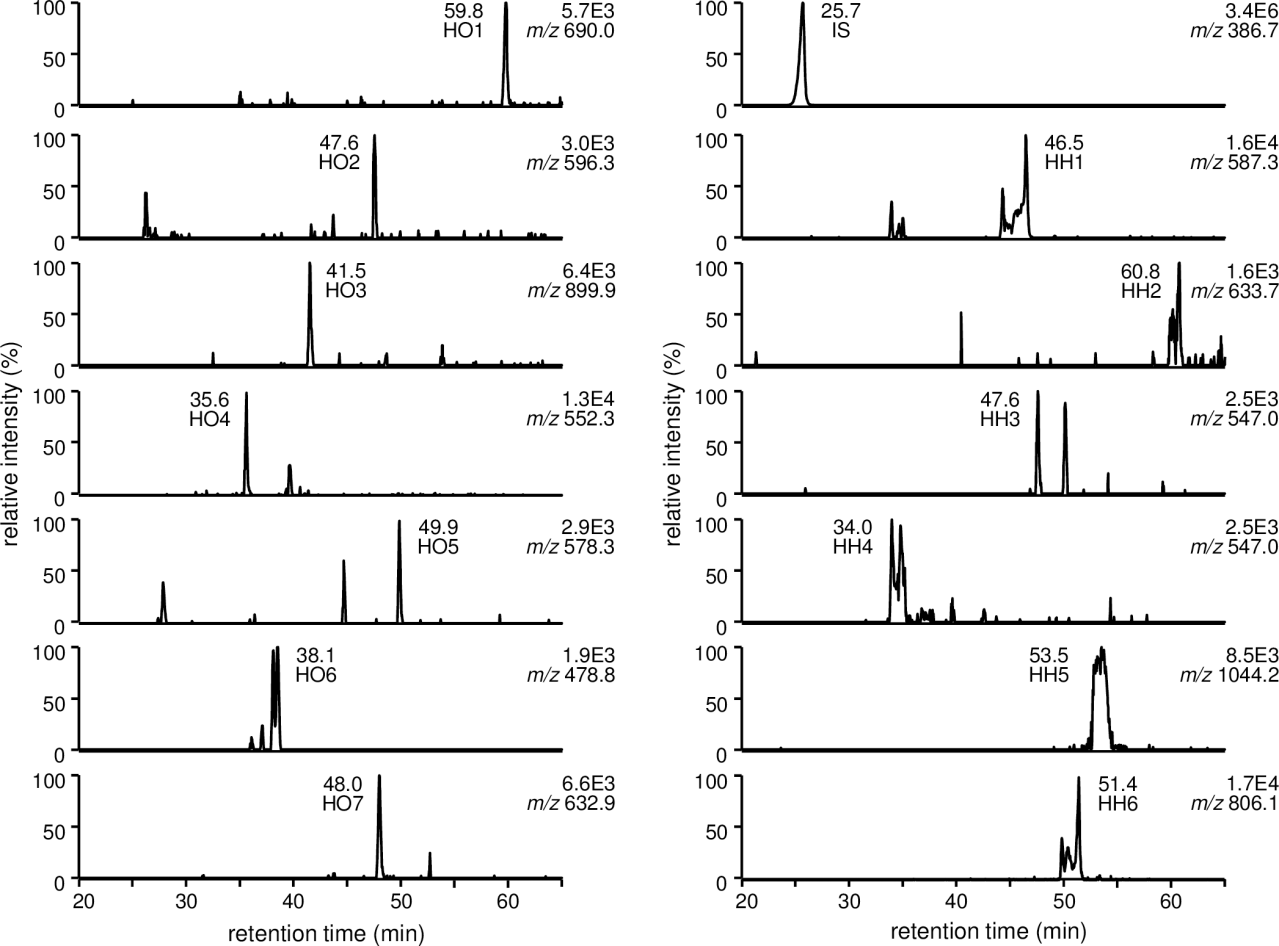
LC/ESI-MS analysis of IS and selected ONE/HNE-modified HSA peptides in the reaction between HSA and 13-HPODE in the presence of AscA and PM.

**Fig 9:**
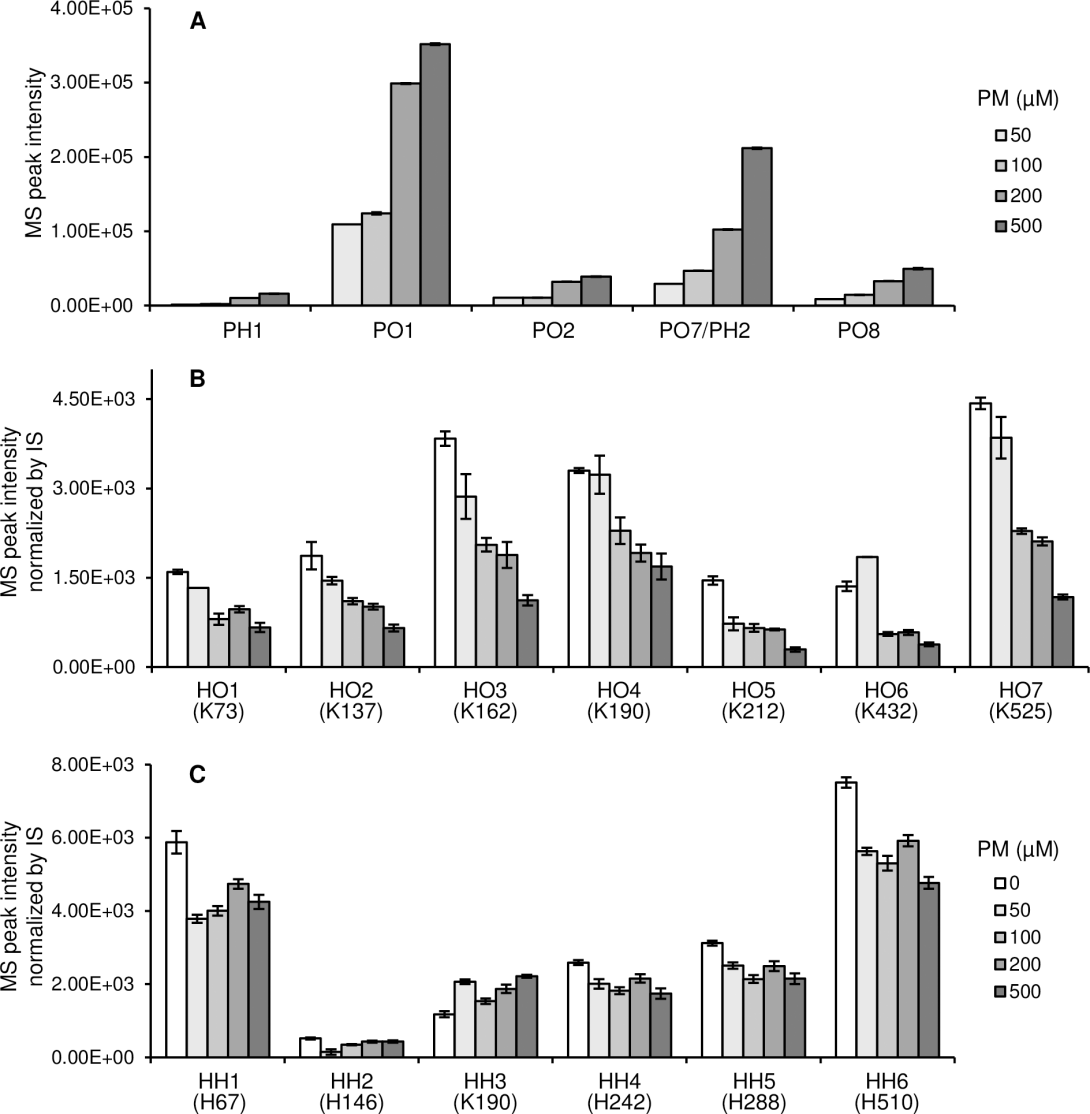
Changes in levels of (A) PM-ONE/HNE adducts, (B) ONE-modified HSA peptides, and (C) HNE-modified HSA peptides in the reactions between HSA and 13-HOPDE in the presence of AscA and increasing concentrations of PM. The information in parentheses indicates modification site. Data are presented as means ± SEM (error bars) from triplicate samples except for HO1 and HO6 at 50 μM PM (from single sample).

The major hydroperoxide derived from LA, 13-HPODE, was employed as a source of ONE and HNE. In the absence of PM, database analyses revealed the presence of 16 ONE-modified and 16 HNE-modified peptides (Table 1, S10 Fig). The modification sites were as follows: H^39^, K^73^, K^136^, K^137^, R^145^, H^146^, K^162^, K^190^, K^205^, K^212^, H^247^, R^337^, K^378^, K^432^, and K^525^ (10 Lys, 3 His, 2 Arg) for ONE modification, and H^39^, H^67^, H^146^, K^190^, K^205^, H^242^, H^247^, H^288^, H^338^, and H^510^ (8 His, 2 Lys) for HNE modification. In the presence of PM, the PM-ONE/HNE adducts PH1, PO1, PO2, PO7/PH2, and PO8 were formed (Fig 7). Changes in the levels of PM-ONE/HNE adducts were monitored, as well as of seven ONE-modified peptides (HO1–7) modified at K^73^, K^137^, K^162^, K^190^, K^212^, K^432^, and K^525^, and six HNE-modified peptides (HH1–6) modified at H^67^, H^146^, K^190^, H^242^, H^247^, and H^288^ (Fig 8). The levels of all PM-ONE/HNE adducts increased dose-dependently in response to PM. The MS peak of PO1 was the most intense, followed by PO7/PH2 and PO8 (Fig 9A). The normalized MS peak intensities of the seven ONE-modified peptides decreased in a PM dose-dependent manner (Fig 9B). In particular, the levels of HO3, HO4, and HO7, modified at K^162^, K^190^, and K^525^, respectively, showed a clear dose-dependent decrease, with 500 μM PM inhibiting their formation by approximately 67%, 56%, and 60%, respectively. In contrast, no dose-dependent change in concentration was observed for the selected HNE-modified peptides (Fig 9C). Changes in levels of ONE/HNE-modified peptides were re-evaluated using MS peak intensity relative to corresponding intact peptides (S11 Fig). Similar patterns of level changes were exhibited with both evaluation methods. However, relative levels of some modified peptides cannot be determined because their intact peptides were not detected as shown in S11 Fig. A possible reason for the disappearance of intact peptide is that reactive sites can be modified by multiple aldehydes derived from 13-HPODE decomposition.

**Table 1:** List of ONE- and HNE-modified HSA peptides identified from the reaction of HSA with 13-HPODE in the presence of AscA.

Peptide Sequence	Modification		Peptides monitored
	Site	Type	
ALVLIAFAQYLQQCPFEDH*VK	H39	ONE, HNE	
SLH*TLFGDK	H67	HNE	HH1
SLH*TLFGDKLCTVATLR	H67	HNE	
SLHTLFGDK*LCTVATLR	K73	ONE, ONE−H_2_O	HO1
LVRPEVDVMCTAFHDNEETFLK*K	K136	ONE−H_2_O	
K*YLYEIAR	K137	ONE−H_2_O	HO2
R*H*PYFYAPELLFFAK	R145/H146	ONE/HNE	
H*PYFYAPELLFFAK	H146	HNE	HH2
RH*PYFYAPELLFFAK	H146	ONE, HNE	
RYK*AAFTECCQAADK	K162	ONE, ONE−H_2_O	
YK*AAFTECCQAADK	K162	ONE, ONE−H_2_O	HO3
LDELRDEGK*ASSAK	K190	ONE−H_2_O, HNE−2H_2_O	HO_4_, HH_3_
CASLQK*FGER	K205	ONE−H_2_O, HNE−2H_2_O	
AFK*AWAVAR	K212	ONE−H_2_O	HO5
VH*TECCHGDLLECADDR	H242	HNE	
VH*TECCHGDLLECADDRADLAK	H242	HNE	HH4
VH*TECCH*GDLLECADDR	H242/H247	HNE/ONE, HNE	
VH*TECCH*GDLLECADDRADLAK	H242/H247	HNE/HNE	
SH*CIAEVENDEMPADLPSLAADFVESK	H288	HNE	HH5
R*H*PDYSVVLLLR	R337/H338	ONE/HNE	
RH*PDYSVVLLLR	H338	HNE	
VFDEFK*PLVEEPQNLIK	K378	ONE	
NLGK*VGSK	K432	ONE	HO6
EFNAETFTFH*ADICTLSEK	H510	HNE	HH6
K*QTALVELVK	K525	ONE−H_2_O	HO7

* indicates a modification site.

### LC/ESI-SRM/MS analysis of PM adducts in the cell system subjected to oxidative stress

**Fig 10:**
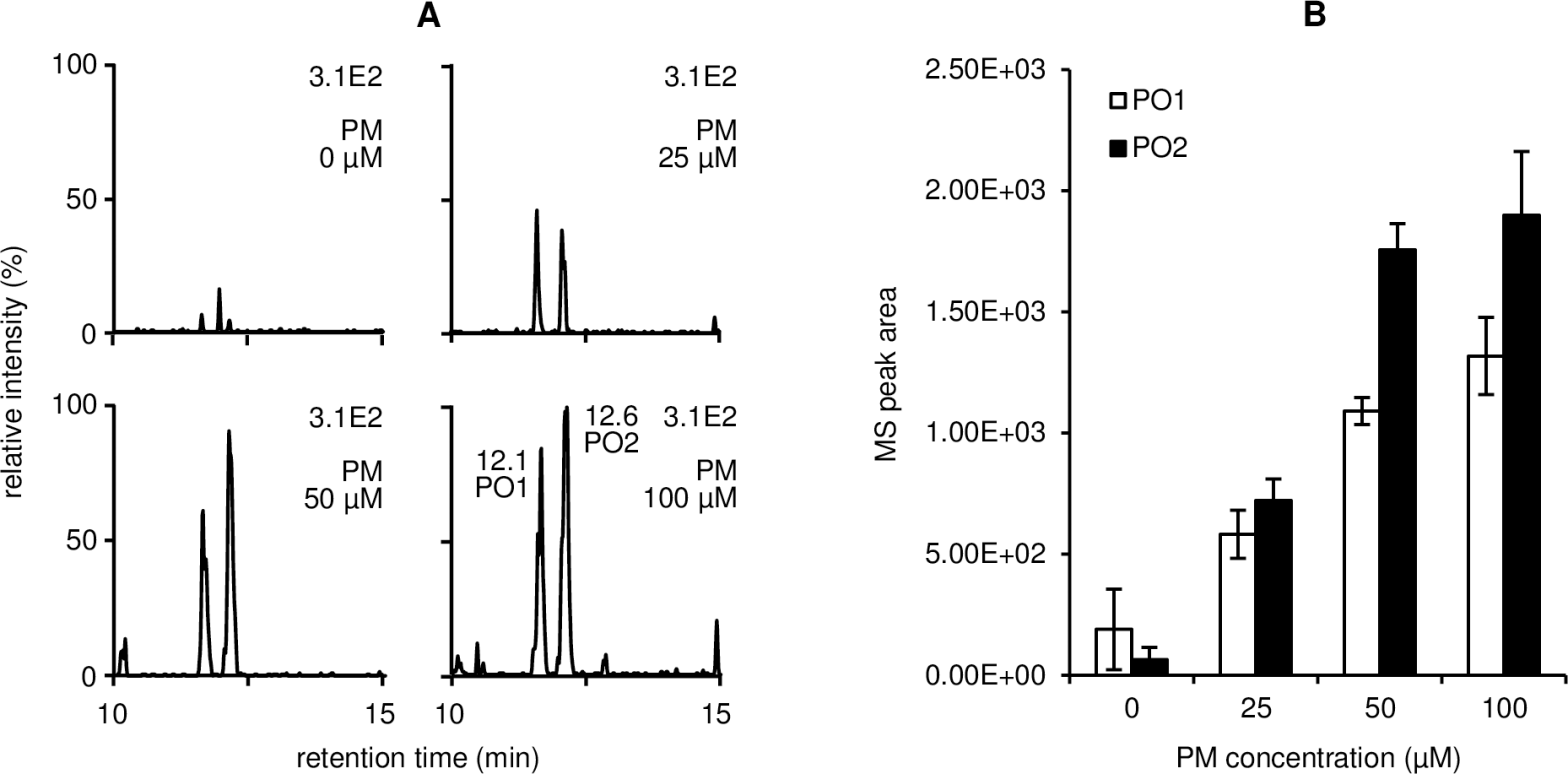
(A) LC/ESI-SRM/MS analysis of PO1 and PO2 extracted from cell culture medium after incubation with Ang II in the presence of AscA and CuSO_4_. (B) Changes in levels of PO1 and PO2. Data are presented as means ± SEM (error bars) from triplicate samples.

SRM conditions such as mass transition, collision energy (eV), and *S*-lens RF amplitude (V), were optimized for PM adducts as follows: PH1, *m/z* 325.1 → 152.1, 24, 107; PO1/PO2, *m/z* 305.2 → 152.1, 25, 110; PO7/PH2, *m/z* 289.2 → 152.1, 23, 110; PO8, *m/z* 287.2 → 138.1, 14, 90. After the pretreatment of PM (0–100 μM), the cell were incubated with Ang II in the presence of AscA and CuSO_4_ to induce lipid peroxidation followed by decomposition of lipid hydroperoxides to reactive aldehydes. LC/ESI-SRM/MS analysis revealed the presence of PO1 and PO2 even without PM treatment (Fig 10A), indicating the formation of PM in the cell. By the addition of PM, levels of PO1 and PO2 were increased in a dose-dependent manner (Fig 10B).

## Discussion

Upon reaction with proteins, ONE can form Michael addition products with Cys, His, and Lys that undergo ring-closure to furan or condensation with another Lys to form pyrrole crosslinks [6]. However, the preferential reaction of ONE is Schiff base formation, which modifies Arg [8, 29] and Lys [9] to produce a substituted imidazole and a stable 4-ketoamide (an apparent ONE-Lys Michael addition product), respectively. Moreover, ONE mediates conversion of the N-terminal amino acid to α-ketoamide through a Schiff base intermediate [7, 30]. Similarly, the reaction between PM and ONE is proposed to begin with nucleophilic attack of the primary amine of PM on the C-1 aldehyde of ONE, yielding a Schiff base intermediate after the loss of water (Fig 6A). This imine undergoes phenolic addition to form pyrido-1,3-oxazine (PO1/PO2). Isomerization of the double bond to the *cis*-orientation, followed by ring closure and dehydration, yields the multicyclic pyrrolo[2,1-*b*][1,3]oxazine (PO8) [28]. On the other hand, the acidic phenol can protonate the carbonyl and hold it in position to facilitate the attack of amine, as proposed for the reaction between PM and 1,4-dicarbonyls [26]. Aromatization of the resulting hydroxy pyrroline produces a pyrrole (PO7). The formation of both PO7 and PO8 was observed after 24 h and their concentrations gradually increased during the 5 day incubation (Fig 1). Although the MS intensity of PO7 was higher than that of the other adducts, PO1, PO7, and PO8 are major stable products of the reaction between PM and ONE. In contrast to ONE, Michael addition is the predominant reaction of HNE with Cys, His, and Lys, although there is mass spectrometric evidence for the formation of an HNE-derived Schiff base [31] and a dehydrated Michael addition product [29] that are stabilized by specific protein microenvironments. It has been reported that 5'-*O*-pentyl-PM produces isomers of the Schiff base upon reaction with HNE for 2 h [28]. In our reaction between PM and HNE (Fig 3), PH1 was the major product at the beginning of the incubation and underwent dehydration to form PH2 (PM + HNE − 2H_2_O), which has identical LC-MS and MS/MS properties to ONE-derived PO7 (PM + ONE − 2H_2_O + 2H). Based on the previous study and our present results, PH1 is expected to be a hydrated Schiff base (PM + HNE − H_2_O + H_2_O) (Fig 6B). We reasoned that hydration occurred because our reaction system used only phosphate buffer (pH 7.4), whereas Amarnath [28] used a 3:2 mixture of phosphate buffer (pH 7.4) and ACN. NMR analysis of PO7 (PH2) supported the presence of a pyrrole ring (Fig 5). Comparison of the amounts of PO7 (PH2) formed in the ONE and HNE reactions showed that ONE produced approximately twice the PO7 (data not shown).

HSA is the most abundant protein in human plasma (66.5 kDa, *t*_1/2_ = 19 d) and constitutes about 60% of the total blood protein. HSA plays important roles in maintaining osmotic pressure and carrying hydrophobic/lipophilic molecules through the circulatory system. In addition, HSA is the main target of chemical stresses because it is highly abundant, circulates throughout the body, and contains many reactive (nucleophilic) amino acids such as 59 Lys, 16 His, and one free Cys residue. Therefore, HSA was employed as a model protein in the present study to investigate the inhibition effect of PM on protein modification induced by ONE and HNE. Complete amino acid sequencing of HSA has been achieved using two different complementary proteases (trypsin and Glu-C) and MS in both positive and negative ionization mode [32]. Several research groups have identified HNE-derived modification sites on HSA; of these, the following sites were identified more than twice from four independent studies [32–35]: C^34^, H^67^, H^146^, K^199^, H^242^, H^288^, H^338^, H^510^, and K^525^. However, the preferential ONE modification sites on HSA have not been reported. In the present study, 44 ONE modification sites were identified with a FDR of 0.01 from the reaction of HSA and ONE (concentration ratio = 1:10) (S1 Table). Using the same reaction conditions and database search criteria, 59 HNE modification sites were identified (S2 Table). However, the ONE and HNE modifications were reduced to 15 and 10 sites (Table 1), respectively, when 13-HPODE was used as a source of reactive aldehydes. This result can be explained in terms of competition among aldehydes for the reactive sites. In addition to ONE and HNE, modifications by other aldehydes, such as 4-hydroperxy-2(*E*)-nonenal (HPNE), 9,12-dioxo-10(*E*)-dodecenoic acid, and 9-hydroxy-12-oxo-10(*E*)-dodecenoic acid, were also observed. A substantial number of HPNE modifications (23 sites) were identified (S3 Table), as reported in our previous study, in which tryptic HSA peptides modified with a 1:1 mixture of ^12^C and ^13^C aldehyde isomers were analyzed by isotope data dependent scan/MS [36]. HSA consists of three repeating domains (I–III), each of which is divided into two subdomains (A and B). The crystal structure analyses of HSA complexed with various ligands have implicated subdomains IIA and IIIA in drug binding, and domains I and III in fatty acid binding [37, 38]. Distributions of modification sites identified in the present study are as follows: IA/2, IB/6, IIA/3, IIB/2, IIIA/1, IIIB /1 for ONE (subdomain/number of modification) and IA/2, IB/2, IIA/4, IIB/1, IIIA/0, IIIB /1 for HNE. Thus, the major modification sites for ONE and HNE are subdomains IB and IIA, respectively. The current study used HSA derived from lyophilized powder and fatty acid free, which could be different from HSA in plasma in terms of binding and physicochemical properties [37]. The previous study of HSA adduction by HNE have compared a fatty acid free HSA with purified HSA, and reported that there were no significant differences in the modification site and reactivity of modified His between two HSAs [34]. However, it is still possible that some of ONE/HNE modification sites are not available in the biological systems.

To evaluate the effect of PM on the chemical modification of HSA by ONE and HNE, changes in the levels of selected PM-ONE/HNE adducts and ONE/HNE-modified tryptic HSA peptides were monitored. When HSA was allowed to react with ONE in the presence of PM, four major PM-ONE adducts (PO1, PO2, PO7, and PO8) were detected and increased dose-dependently upon treatment with PM (S2 and S3 Figs). Concomitantly, the formation of selected HSA peptides ONE-modified at Lys residues decreased in a dose-dependent manner (S4 and S5 Figs). Reaction between HSA, HNE, and PM resulted in the detection of PH1 and PH2 together with HSA peptides HNE-modified at His or Lys residues. Although the concentrations of PM-HNE adducts increased in a PM dose-dependent manner (S7 Fig), a dose-dependent decrease was observed for only one peptide, HNE-modified at a Lys residue (S9 Fig). The 13-HPODE and AscA system is known to generate ONE and HNE with other reactive aldehydes [5]. The addition of HSA and PM resulted in simultaneous formation of the PM-ONE/HNE adducts (Fig 7), and the concentrations of these adducts increased dose-dependently in response to PM (Fig 9A). This increase in adduct concentration coincided with a dose-dependent decrease in ONE-modified HSA peptides but not with HNE-modified peptides (Figs 9B and 9C). PM (500 μM) inhibited the formation of ONE-modified HSA peptides derived from ONE (300 μM) and 13-HPODE (3 mM) by a maximum of 75% and 67%, respectively. The biological concentration of ONE is in the range of nanomolar to lower micromolar [29]. PM is present in the nanomolar range in human plasma, and is increased to about 100 μM upon pharmacological supplementation [30]. Thus, plasma PM concentrations could be sufficient to scavenge the ambient concentration of ONE. In addition, it has been reported that PM forms pyrroles with lipid-derived 4-ketoaldehydes 3 orders of magnitude faster than the ε-amino group of *N*-α-acetyl-Lys [27]. PM was also shown to react with ONE much more rapidly than Lys [28], suggesting the possible use of PM-ONE adducts as a dosimeter for ONE production. To evaluate the inhibition effect of PM in cell culture subjected to oxidative stress, the cell were treated with Ang II which activates phospholipase A2 and NADPH oxidase that results in the induction of lipid peroxidation. AscA and Cu ions can then decompose lipid hydroperoxides to generate reactive aldehydes ONE and HNE. PM-ONE adducts PO1 and PO2 were detected in the cell system (Fig 10A) and further increased upon the treatment with increasing concentrations of PM (Fig 10B).

In summary, our results indicate that PM can inhibit lipid hydroperoxide-derived damage to proteins by trapping ONE preferentially. We characterized several PM adducts generated by the reaction of PM with ONE or HNE, and found that PO7 (a PM-ONE adduct) and PH2 (a PM-HNE adduct) share an identical structure containing a pyrrole ring. Upon incubation with HSA, Lys residues were the most favorable sites for both ONE and HNE modification, and the number of HNE-modified peptides was higher than that of ONE-modified peptides. However, when HSA was incubated with 13-HPODE and AscA, ONE modified Lys residues more than did HNE. HNE mainly reacted with His residues, thereby reflecting the relative reactivity of aldehydes towards amino acid residues. In the presence of PM, only the concentrations of ONE-modified HSA peptides were reduced significantly and dose-dependently, and the levels of PM-ONE adducts increased concomitantly. The inhibition effect of PM was then confirmed in cell culture subjected to oxidative stress by measuring PM-ONE adducts, possible dosimeters for ONE production to determine the levels of lipid peroxidation.

## Supporting information

S1 Fig Total ion current chromatogram (TICC) of MS for (A) ONE- and (B) HNE-modified HSA peptides.
(TIF)

S2 Fig LC/ESI-MS analysis of PM-ONE adducts in the reaction between HSA and ONE in the presence of PM. (TIF)

S3 Fig Changes in levels of PM-ONE adducts in the reactions between HSA and ONE (A, 300 μM; B, 3 μM) in the presence of increasing concentrations of PM.
(TIF)

S4 Fig LC/ESI-MS analysis of IS and selected ONE-modified HSA peptides in the reaction between HSA and ONE. (PDF)

S5 Fig Changes in levels of ONE-modified HSA peptides in the reactions between HSA and ONE (A, 300 μM; B, 3 μM) in the presence of increasing concentrations of PM. The information in parentheses indicates modification site.
(TIF)

S6 Fig LC/ESI-MS analysis of PM-HNE adducts in the reaction between HSA and HNE in the presence of PM. (TIF)

S7 Fig Changes in levels of PM-HNE adducts in the reactions between HSA and HNE (A, 300 μM; B, 3 μM) in the presence of increasing concentrations of PM.
(TIF)

S8 Fig LC/ESI-MS analysis of IS and selected HNE-modified HSA peptides in the reaction between HSA and HNE. (PDF)

S9 Fig Changes in levels of HNE-modified HSA peptides in the reactions between HSA and HNE (A, 300 μM; B, 3 μM) in the presence of increasing concentrations of PM. The information in parentheses indicates modification site.
(TIF)

S10 Fig MS/MS spectra of ONE- and HNE-modified HSA peptides listed in Table 1. (PDF)

S11 Fig Changes in levels (as a relative intensity to corresponding intact peptide) of (A) ONE-modified HSA peptides and (B) HNE-modified HSA peptides in the reactions between HSA and 13-HOPDE in the presence of AscA and increasing concentrations of PM. The information in parentheses indicates modification site. Data are presented as means ± SEM (error bars) from triplicate samples except for HO1 at 50 μM PM (from single sample).
(TIF)

S1 Table List of ONE-modified HSA peptides identified from the reaction between HSA (30 μM) and ONE (300 μM). * indicates a modification site. O indicates a peptide identified automatically. Δ indicates a peptide identified manually.
(PDF)

S2 Table List of HNE-modified HSA peptides identified from the reaction between HSA (30 μM) and HNE (300 μM). * indicates a modification site. O indicates a peptide identified automatically. Δ indicates a peptide identified manually.
(PDF)

S3 Table List of ONE-, HNE-, and HPNE-modified HSA peptides identified from the reaction of HSA with 13-HPODE in the presence of AscA. * indicates a modification site.
(PDF)

## References

[1] Stadtman ER. Protein oxidation in aging and age-related diseases. Ann New York Acad Sci. 2001; 928: 22–38. 11795513 10.1111/j.1749-6632.2001.tb05632.x

[2] Finkel T. Signal transduction by reactive oxygen species. J cell Biol. 2011; 194: 7–15. doi: 10.1083/jcb.201102095 21746850 10.1083/jcb.201102095 PMC3135394

[3] Stadtman ER, Berlett BS (1991) Fenton chemistry. Amino acid oxidation. J Biol Chem 266: 17201–17211. 1894614

[4] Speed N, Blair IA. Cyclooxygenase- and lipoxygenase-mediated DNA damage. Cancer Metastas- Rev. 2011; 30: 437–447. doi: 10.1007/s10555-011-9298-8 22009064 10.1007/s10555-011-9298-8 PMC3237763

[5] Lee SH, Oe T, Blair IA. Vitamin C-induced decomposition of lipid hydroperoxides to endogenous genotoxins. Science. 2001; 292: 2083–2086. doi: 10.1126/science.1059501 11408659 10.1126/science.1059501

[6] Sayre LM, Lin D, Yuan Q, Zhu X, Tang X. Protein adducts generated from products of lipid oxidation: focus on HNE and one. Drug Metab Rev. 2006; 38: 651–675. doi: 10.1080/03602530600959508 17145694 10.1080/03602530600959508

[7] Lee SH, Goto T, Oe T. A novel 4-oxo-2(*E*)-nonenal-derived modification to angiotensin II: oxidative decarboxylation of N-terminal aspartic acid. Chem Res Toxicol. 2008; 21: 2237–2244. doi: 10.1021/tx800316v 19548347 10.1021/tx800316v

[8] Oe T, Lee SH, Silva Elipe MV, Arison BH, Blair IA. A novel lipid hydroperoxide-derived modification to arginine. Chem Res Toxicol. 2003; 16: 1598–1605. doi: 10.1021/tx034178l 14680374 10.1021/tx034178l

[9] Zhu X, Sayre LM. Long-lived 4-oxo-2-enal-derived apparent lysine michael adducts are actually the isomeric 4-ketoamides. Chem Res Toxicol. 2007; 20: 165–170. doi: 10.1021/tx600295j 17305402 10.1021/tx600295j

[10] Baynes JW, Thorpe SR. Role of oxidative stress in diabetic complications: a new perspective on an old paradigm. Diabetes. 1999; 48: 1–9. 9892215 10.2337/diabetes.48.1.1

[11] Vistoli G, De Maddis D, Cipak A, Zarkovic N, Carini M, Aldini G. Advanced glycoxidation and lipoxidation end products (AGEs and ALEs): an overview of their mechanisms of formation. Free Radic Res. 2013; 47 Suppl 1: 3–27. doi: 10.3109/10715762.2013.815348 23767955 10.3109/10715762.2013.815348

[12] Glomb MA, Monnier VM. Mechanism of protein modification by glyoxal and glycolaldehyde, reactive intermediates of the Maillard reaction. J Biol Chem. 1995; 270: 10017–10026. 7730303 10.1074/jbc.270.17.10017

[13] Ahmed MU, Brinkmann Frye E, Degenhardt TP, Thorpe SR, Baynes JW. N-epsilon-(carboxyethyl)lysine, a product of the chemical modification of proteins by methylglyoxal, increases with age in human lens proteins. Biochem J. 1997; 324 (Pt 2): 565–570. 9182719 10.1042/bj3240565 PMC1218467

[14] Shipanova IN, Glomb MA, Nagaraj RH. Protein modification by methylglyoxal: chemical nature and synthetic mechanism of a major fluorescent adduct. Arch Biochem Biophys. 1997; 344: 29–36. doi: 10.1006/abbi.1997.0195 9244378 10.1006/abbi.1997.0195

[15] Osicka TM, Yu Y, Panagiotopoulos S, Clavant SP, Kiriazis Z, Pike RN, et al Prevention of albuminuria by aminoguanidine or ramipril in streptozotocin-induced diabetic rats is associated with the normalization of glomerular protein kinase C. Diabetes. 2000; 49: 87–93. 10615954 10.2337/diabetes.49.1.87

[16] Requena JR, Vidal P, Cabezas-Cerrato J. Aminoguanidine inhibits the modification of proteins by lipid peroxidation derived aldehydes: a possible antiatherogenic agent. Diabetes Res. 1992; 20: 43–49. 1345001

[17] Picard S, Parthasarathy S, Fruebis J, Witztum JL. Aminoguanidine inhibits oxidative modification of low density lipoprotein protein and the subsequent increase in uptake by macrophage scavenger receptors. Proc Natl Acad Sci United States Am. 1992; 89: 6876–6880. 1495978 10.1073/pnas.89.15.6876 PMC49607

[18] Voziyan PA, Hudson BG. Pyridoxamine as a multifunctional pharmaceutical: targeting pathogenic glycation and oxidative damage. Cell Mol life Sci: CMLS. 2005; 62: 1671–1681. doi: 10.1007/s00018-005-5082-7 15905958 10.1007/s00018-005-5082-7 PMC11139091

[19] Booth AA, Khalifah RG, Todd P, Hudson BG. In vitro kinetic studies of formation of antigenic advanced glycation end products (AGEs). Novel inhibition of post-Amadori glycation pathways. J Biol Chem. 1997; 272: 5430–5437. 9038143 10.1074/jbc.272.9.5430

[20] Degenhardt TP, Alderson NL, Arrington DD, Beattie RJ, Basgen JM, Steffes MW, et al Pyridoxamine inhibits early renal disease and dyslipidemia in the streptozotocin-diabetic rat. Kidney Int. 2002; 61: 939–950. doi: 10.1046/j.1523-1755.2002.00207.x 11849448 10.1046/j.1523-1755.2002.00207.x

[21] Onorato JM, Jenkins AJ, Thorpe SR, Baynes JW. Pyridoxamine, an inhibitor of advanced glycation reactions, also inhibits advanced lipoxidation reactions. Mechanism of action of pyridoxamine. J Biol Chem. 2000; 275: 21177–21184. doi: 10.1074/jbc.M003263200 10801874 10.1074/jbc.M003263200

[22] Metz TO, Alderson NL, Chachich ME, Thorpe SR, Baynes JW. Pyridoxamine traps intermediates in lipid peroxidation reactions in vivo: evidence on the role of lipids in chemical modification of protein and development of diabetic complications. J Biol Chem. 2003; 278: 42012–42019. doi: 10.1074/jbc.M304292200 12923193 10.1074/jbc.M304292200

[23] Nagaraj RH, Sarkar P, Mally A, Biemel KM, Lederer MO, Padayatti PS. Effect of pyridoxamine on chemical modification of proteins by carbonyls in diabetic rats: characterization of a major product from the reaction of pyridoxamine and methylglyoxal. Arch Biochem Biophys. 2002; 402: 110–119. doi: 10.1016/S0003-9861(02)00067-X 12051689 10.1016/S0003-9861(02)00067-X

[24] Voziyan PA, Metz TO, Baynes JW, Hudson BG. A post-Amadori inhibitor pyridoxamine also inhibits chemical modification of proteins by scavenging carbonyl intermediates of carbohydrate and lipid degradation. J Biol Chem. 2002; 277: 3397–3403. doi: 10.1074/jbc.M109935200 11729198 10.1074/jbc.M109935200

[25] Kang Z, Li H, Li G, Yin D. Reaction of pyridoxamine with malondialdehyde: mechanism of inhibition of formation of advanced lipoxidation end-products. Amino acids. 2006; 30: 55–61. doi: 10.1007/s00726-005-0209-6 15990947 10.1007/s00726-005-0209-6

[26] Amarnath V, Amarnath K, Amarnath K, Davies S, Roberts LJ. Pyridoxamine: an extremely potent scavenger of 1,4-dicarbonyls. Chem Res Toxicol. 2004; 17: 410–415. doi: 10.1021/tx0300535 15025512 10.1021/tx0300535

[27] Davies SS, Brantley EJ, Voziyan PA, Amarnath V, Zagol-Ikapitte I, Boutaud O, et al Pyridoxamine analogues scavenge lipid-derived gamma-ketoaldehydes and protect against H_2_O_2_-mediated cytotoxicity. Biochemistry. 2006; 45: 15756–15767. doi: 10.1021/bi061860g 17176098 10.1021/bi061860g PMC2597444

[28] Amarnath V, Amarnath K. Scavenging 4-oxo-2-nonenal. Chem Res Toxicol. 2015; 28: 1888–1890. doi: 10.1021/acs.chemrestox.5b00301 26355561 10.1021/acs.chemrestox.5b00301 PMC6579115

[29] Lee SH, Takahashi R, Goto T, Oe T. Mass spectrometric characterization of modifications to angiotensin II by lipid peroxidation products, 4-oxo-2(*E*)-nonenal and 4-hydroxy-2(*E*)-nonenal. Chem Res Toxicol. 2010; 23: 1771–1785. doi: 10.1021/tx100228q 20977208 10.1021/tx100228q

[30] Lee SH, Kyung H, Yokota R, Goto T, Oe T. N-Terminal α-ketoamide peptides: Formation and transamination. Chem Res Toxicol. 2014; 27: 637–648. doi: 10.1021/tx400469x 24568234 10.1021/tx400469x

[31] Rauniyar N, Prokai L. Detection and identification of 4-hydroxy-2-nonenal Schiff-base adducts along with products of Michael addition using data-dependent neutral loss-driven MS3 acquisition: method evaluation through an in vitro study on cytochrome c oxidase modifications. Proteomics. 2009; 9: 5188–5193. doi: 10.1002/pmic.200900116 19771555 10.1002/pmic.200900116 PMC3065305

[32] Goto T, Murata K, Lee SH, Oe T. Complete amino acid sequencing and immunoaffinity clean-up can facilitate screening of various chemical modifications on human serum albumin. Anal Bioanal Chem. 2013; 405: 7383–7395. doi: 10.1007/s00216-013-7146-0 23846590 10.1007/s00216-013-7146-0

[33] Aldini G, Gamberoni L, Orioli M, Beretta G, Regazzoni L, Facino RM, et al Mass spectrometric characterization of covalent modification of human serum albumin by 4-hydroxytrans-2-nonenal. J mass Spectrom. 2006; 41: 1149–1161. http://onlinelibrary.wiley.com/doi/10.1002/jms.1067/full doi: 10.1002/jms.1067 16888752 10.1002/jms.1067

[34] Szapacs ME, Riggins JN, Zimmerman LJ, Liebler DC. Covalent adduction of human serum albumin by 4-hydroxy-2-nonenal: kinetic analysis of competing alkylation reactions. Biochemistry. 2006; 45: 10521–10528. http://pubs.acs.org/doi/abs/10.1021/bi060535q doi: 10.1021/bi060535q 16939204 10.1021/bi060535q

[35] Liu Q, Simpson DC, Gronert S. The reactivity of human serum albumin toward *trans*-4-hydroxy-2-nonenal. J Mass Spectrom. 2012; 47: 411–424. http://onlinelibrary.wiley.com/doi/10.1002/jms.2037/full doi: 10.1002/jms.2037 22689617 10.1002/jms.2037 PMC3531918

[36] Takahashi R, Fujioka S, Oe T, Lee SH. Stable isotope labeling by fatty acids in cell culture (SILFAC) coupled with isotope pattern dependent mass spectrometry for global screening of lipid hydroperoxide-mediated protein modifications. J proteomics. 2017; 166: 101–114. https://www.ncbi.nlm.nih.gov/pubmed/28735093 doi: 10.1016/j.jprot.2017.07.006 28735093 10.1016/j.jprot.2017.07.006

[37] Ashrafi-Kooshk MR, Ebrahimi F, Ranjbar S, Ghobadi S, Moradi N, Khodarahmi R. Comparative studies on drug binding to the purified and pharmaceutical-grade human serum albumins: Bridging between basic research and clinical applications of albumin. Biologicals. 2015; 43: 333–343. doi: 10.1016/j.biologicals.2015.07.003 26254507 10.1016/j.biologicals.2015.07.003

[38] Curry S, Mandelkow H, Brick P, Franks N. Crystal structure of human serum albumin complexed with fatty acid reveals an asymmetric distribution of binding sites. Nat Struct Biol. 1998; 5: 827–835. doi: 10.1038/1869 9731778 10.1038/1869

