# Supplementary figures and images for: Inhibition effect of pyridoxamine on lipid hydroperoxide-derived modifications to human serum albumin
Source: PLoS One. 2018 Apr 19;13(4):e0196050. doi: 10.1371/journal.pone.0196050 (PMC5908094; doi:10.1371/journal.pone.0196050)

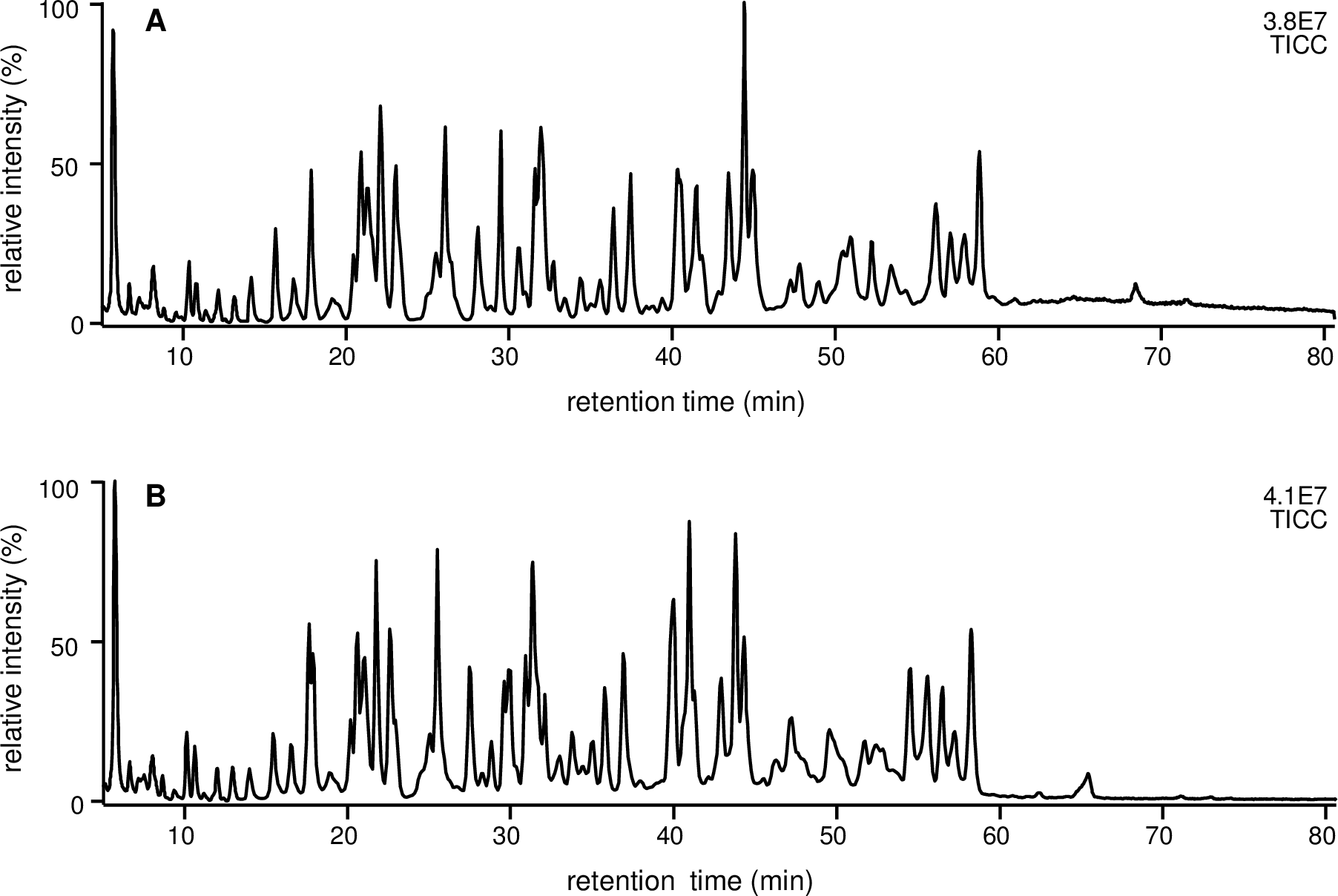

Supplement: S1 Fig — Total ion current chromatogram (TICC) of MS for (A) ONE- and (B) HNE-modified HSA peptides. (TIF) [file pone.0196050.s001.tif]

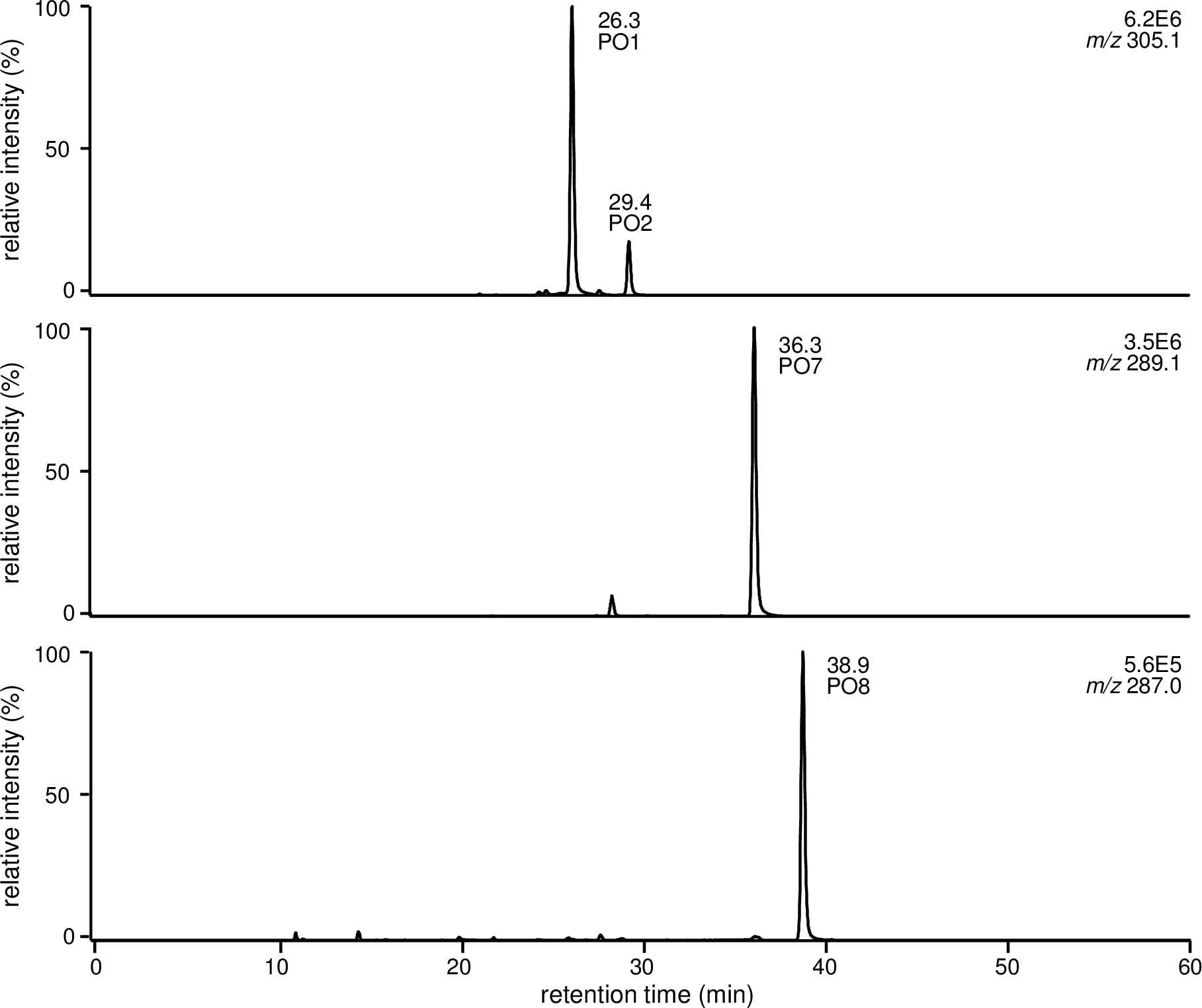

Supplement: S2 Fig — (TIF) [file pone.0196050.s002.tif]

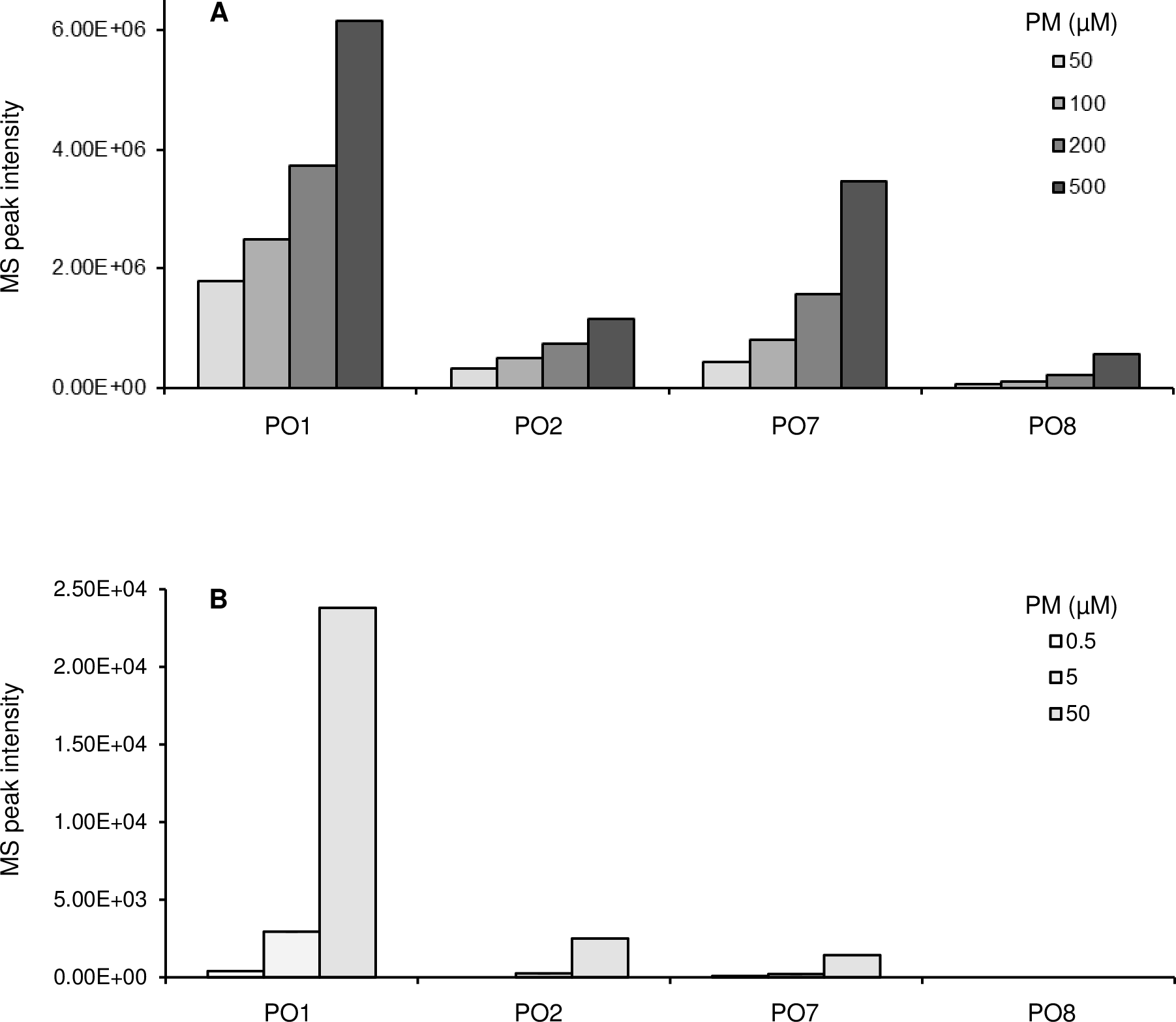

Supplement: S3 Fig — Changes in levels of PM-ONE adducts in the reactions between HSA and ONE (A, 300 μM; B, 3 μM) in the presence of increasing concentrations of PM. (TIF) [file pone.0196050.s003.tif]

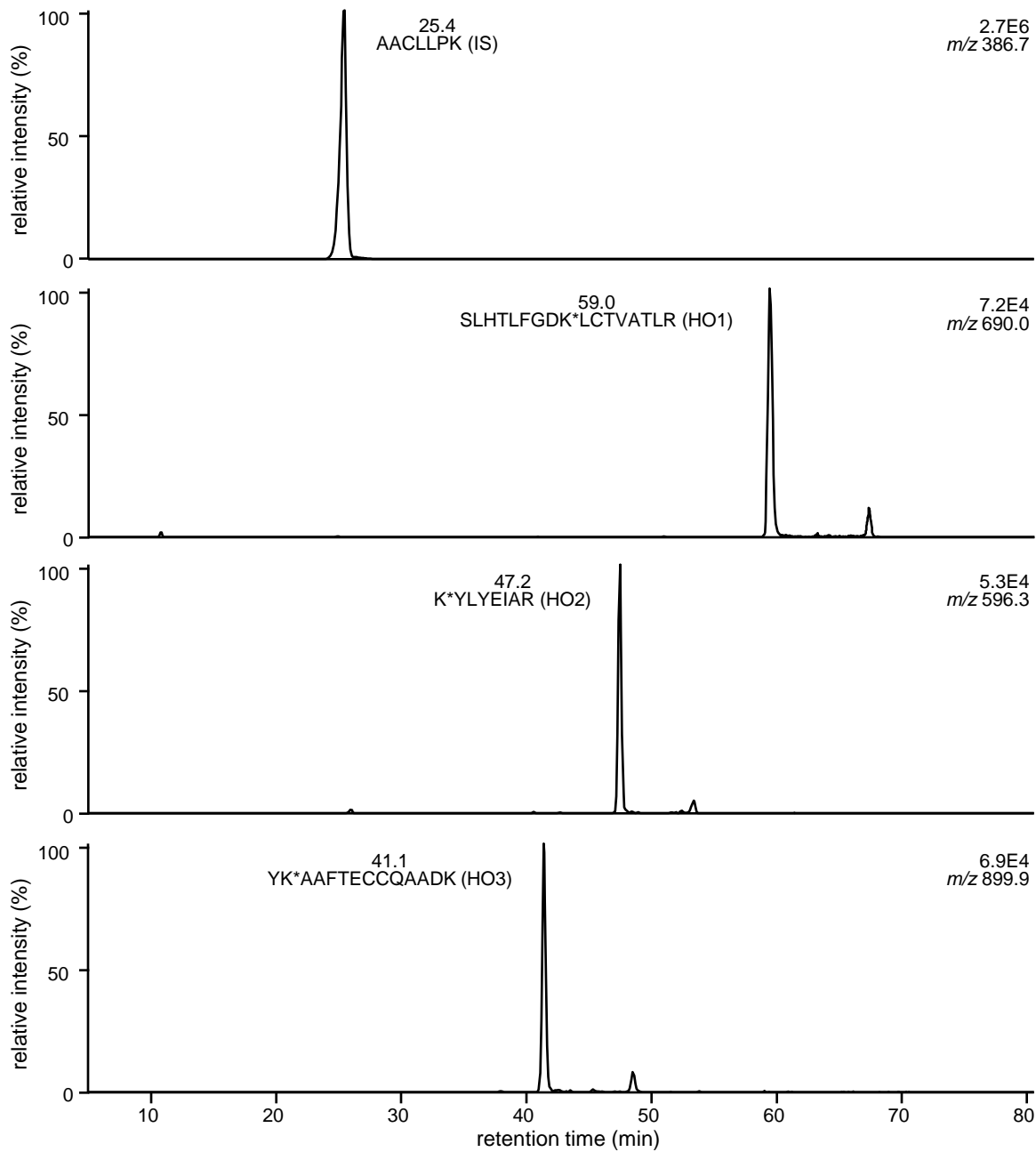

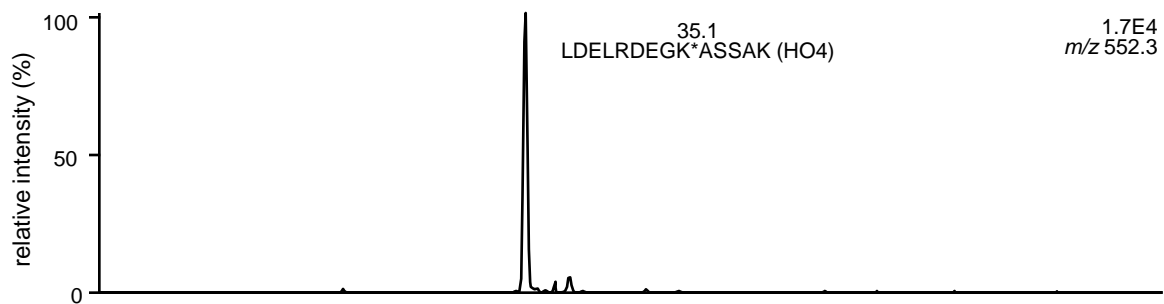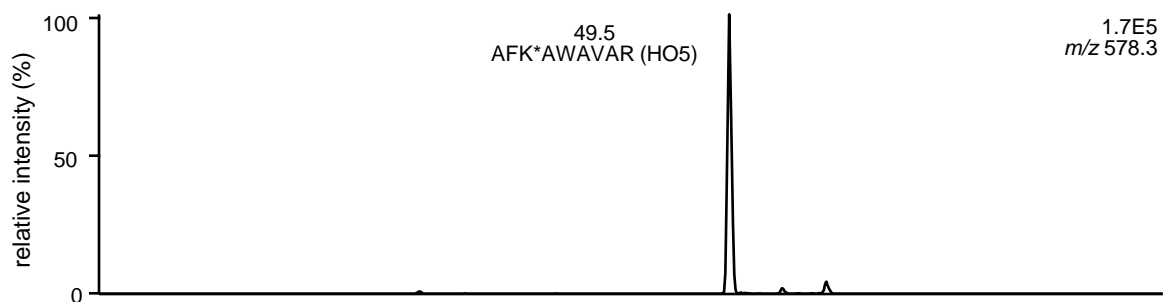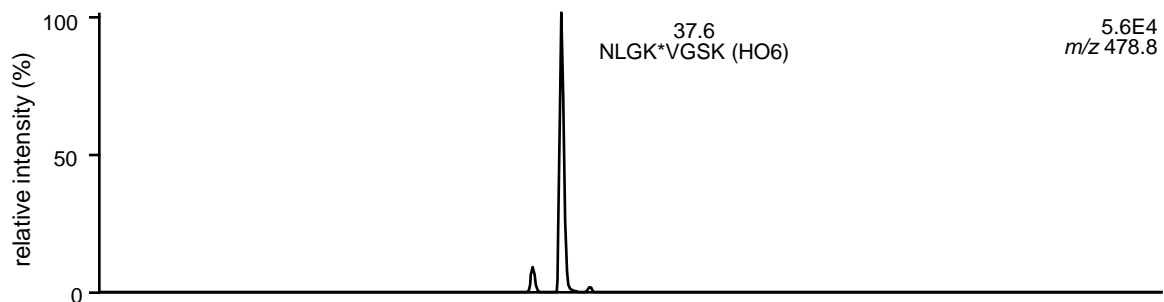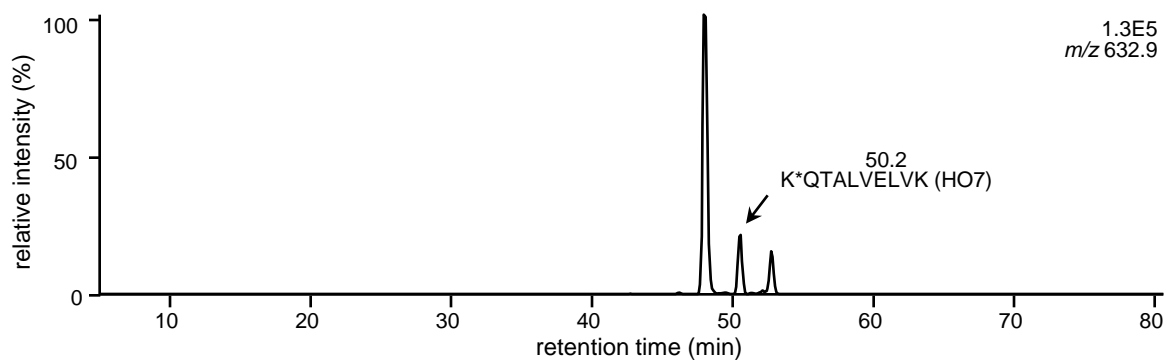

Supplement: S4 Fig — (PDF) [file pone.0196050.s004.pdf]

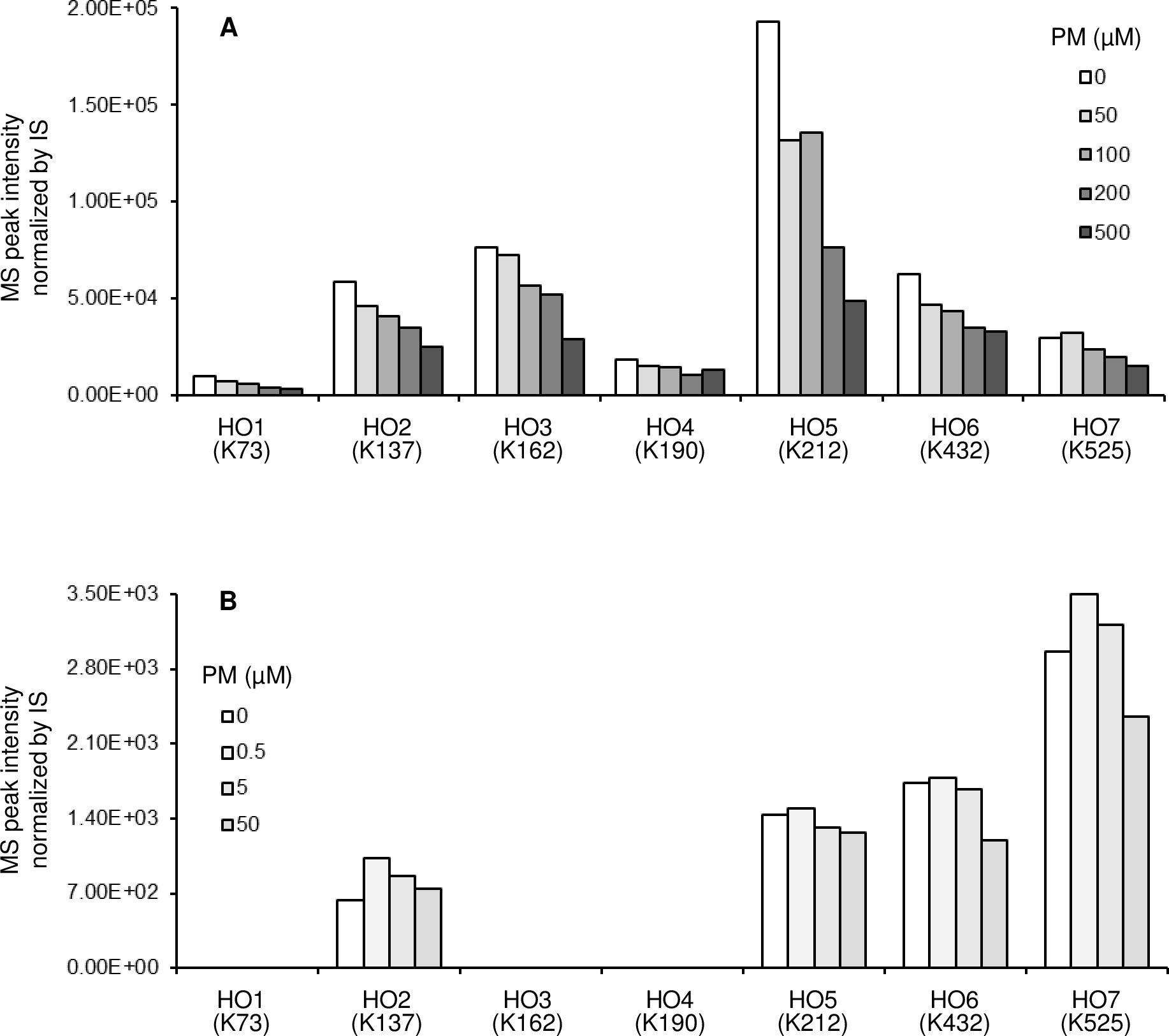

Supplement: S5 Fig — Changes in levels of ONE-modified HSA peptides in the reactions between HSA and ONE (A, 300 μM; B, 3 μM) in the presence of increasing concentrations of PM. The information in parentheses indicates modification site. (TIF) [file pone.0196050.s005.tif]

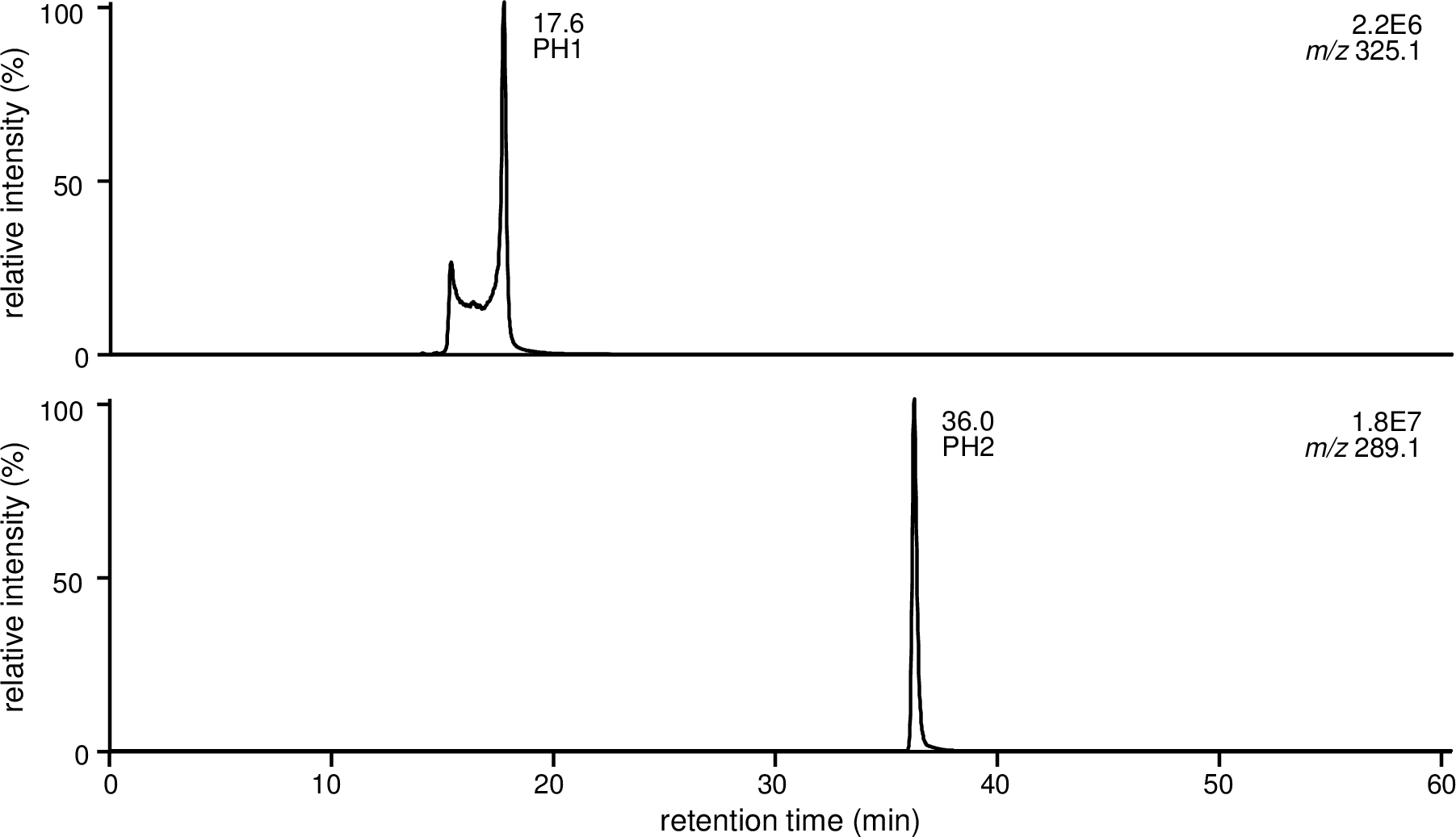

Supplement: S6 Fig — (TIF) [file pone.0196050.s006.tif]

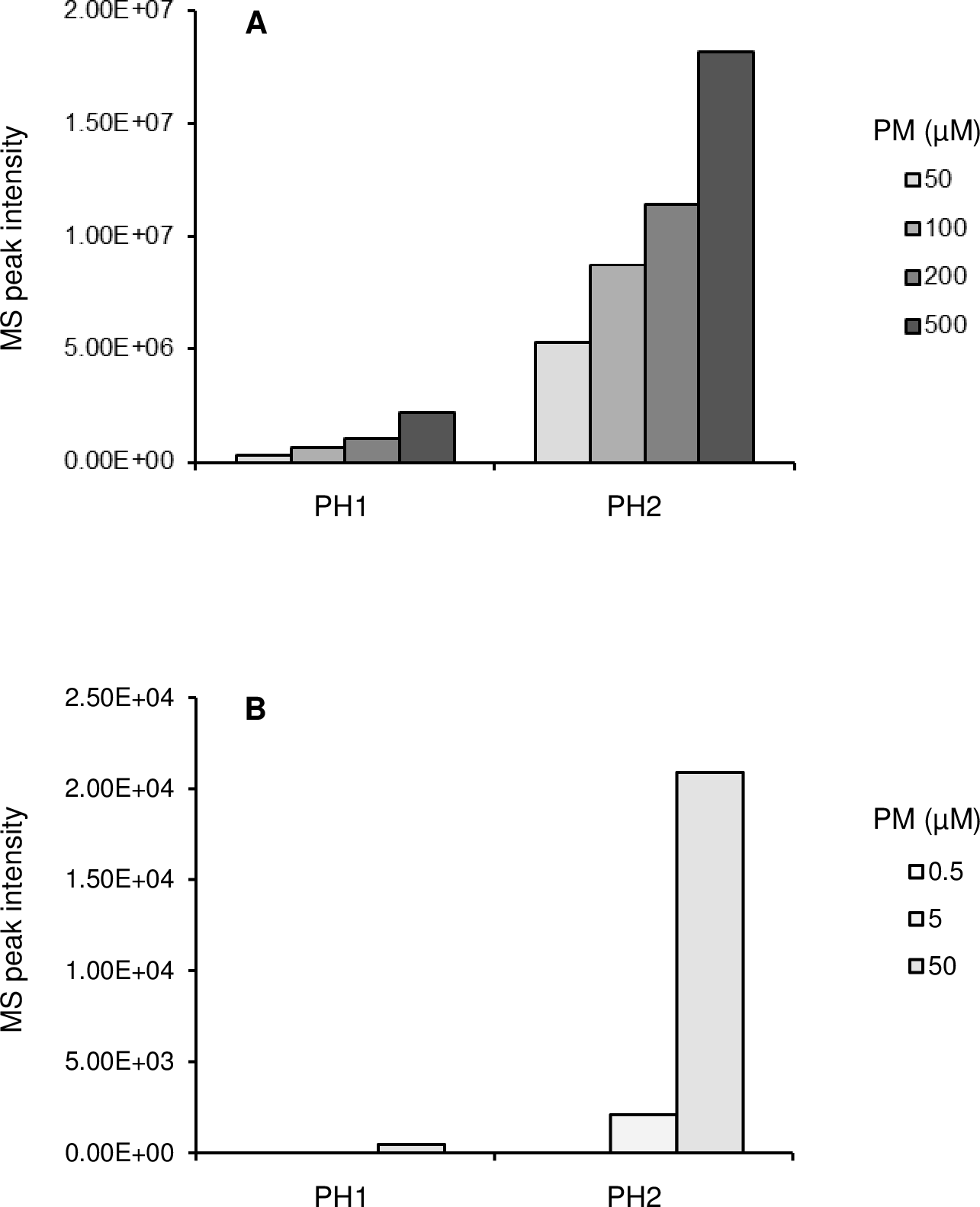

Supplement: S7 Fig — Changes in levels of PM-HNE adducts in the reactions between HSA and HNE (A, 300 μM; B, 3 μM) in the presence of increasing concentrations of PM. (TIF) [file pone.0196050.s007.tif]

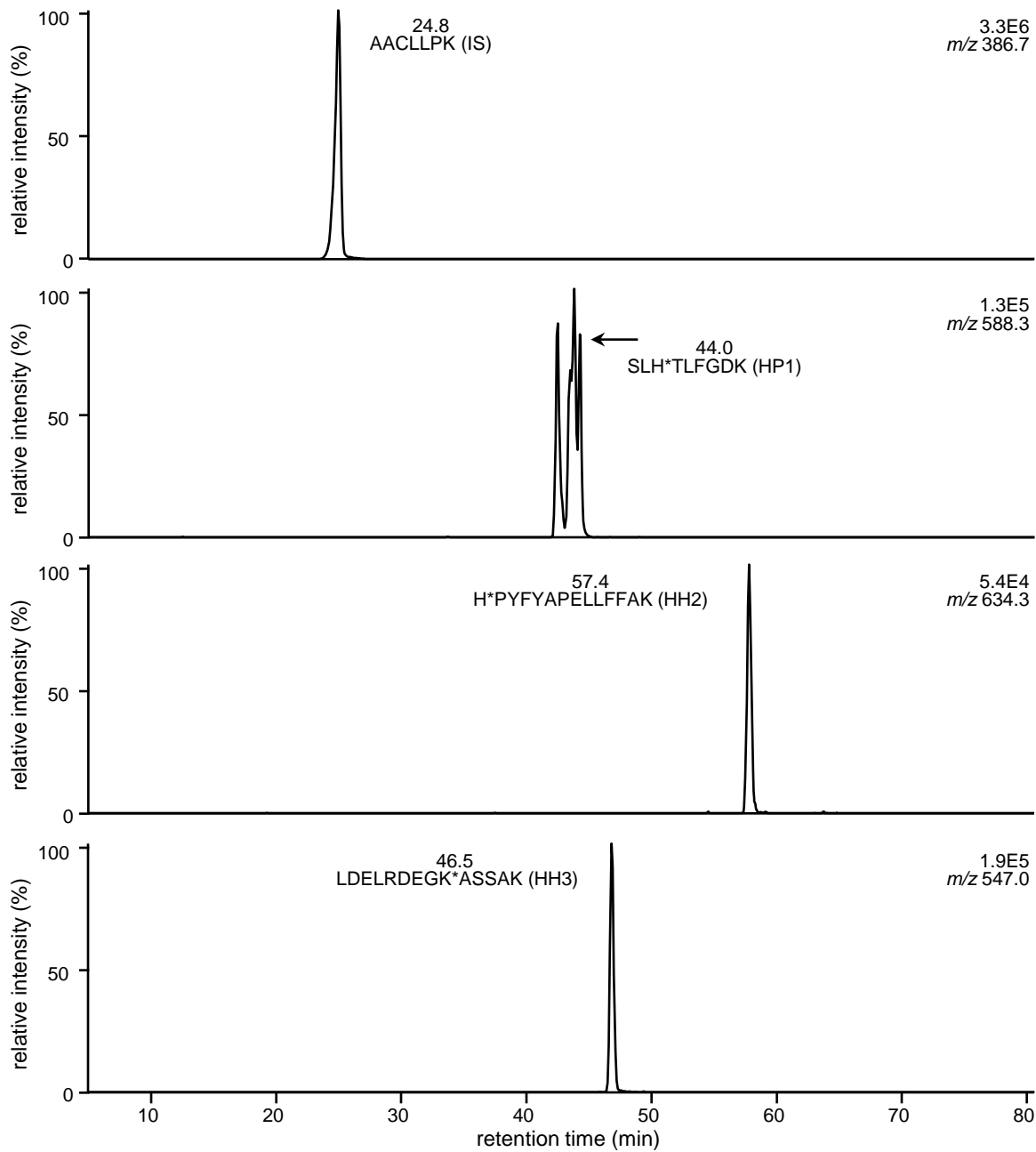

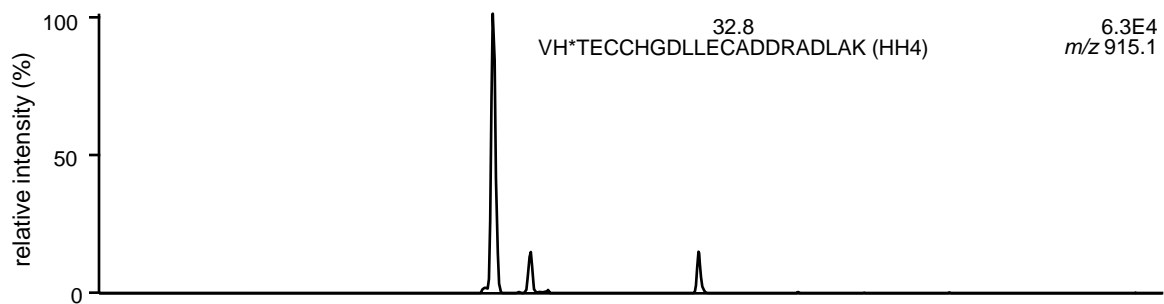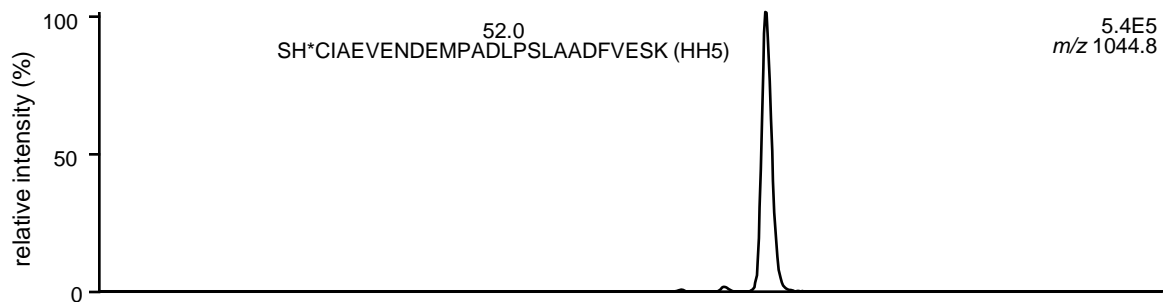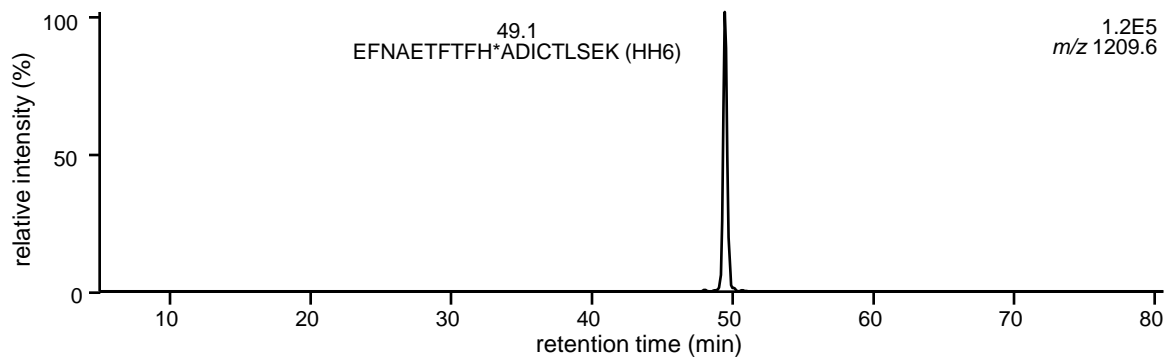

Supplement: S8 Fig — (PDF) [file pone.0196050.s008.pdf]

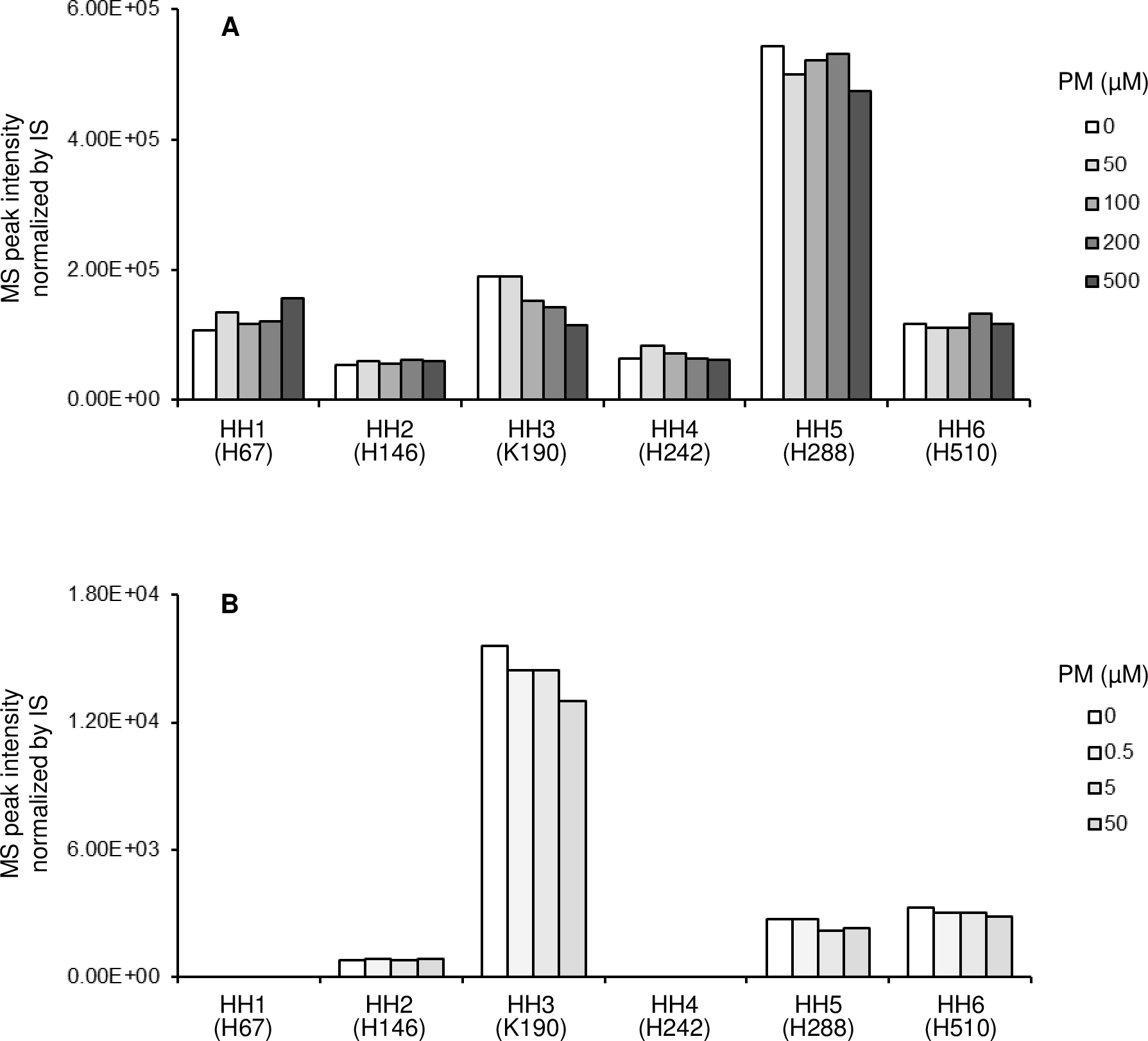

Supplement: S9 Fig — Changes in levels of HNE-modified HSA peptides in the reactions between HSA and HNE (A, 300 μM; B, 3 μM) in the presence of increasing concentrations of PM. The information in parentheses indicates modification site. (TIF) [file pone.0196050.s009.tif]

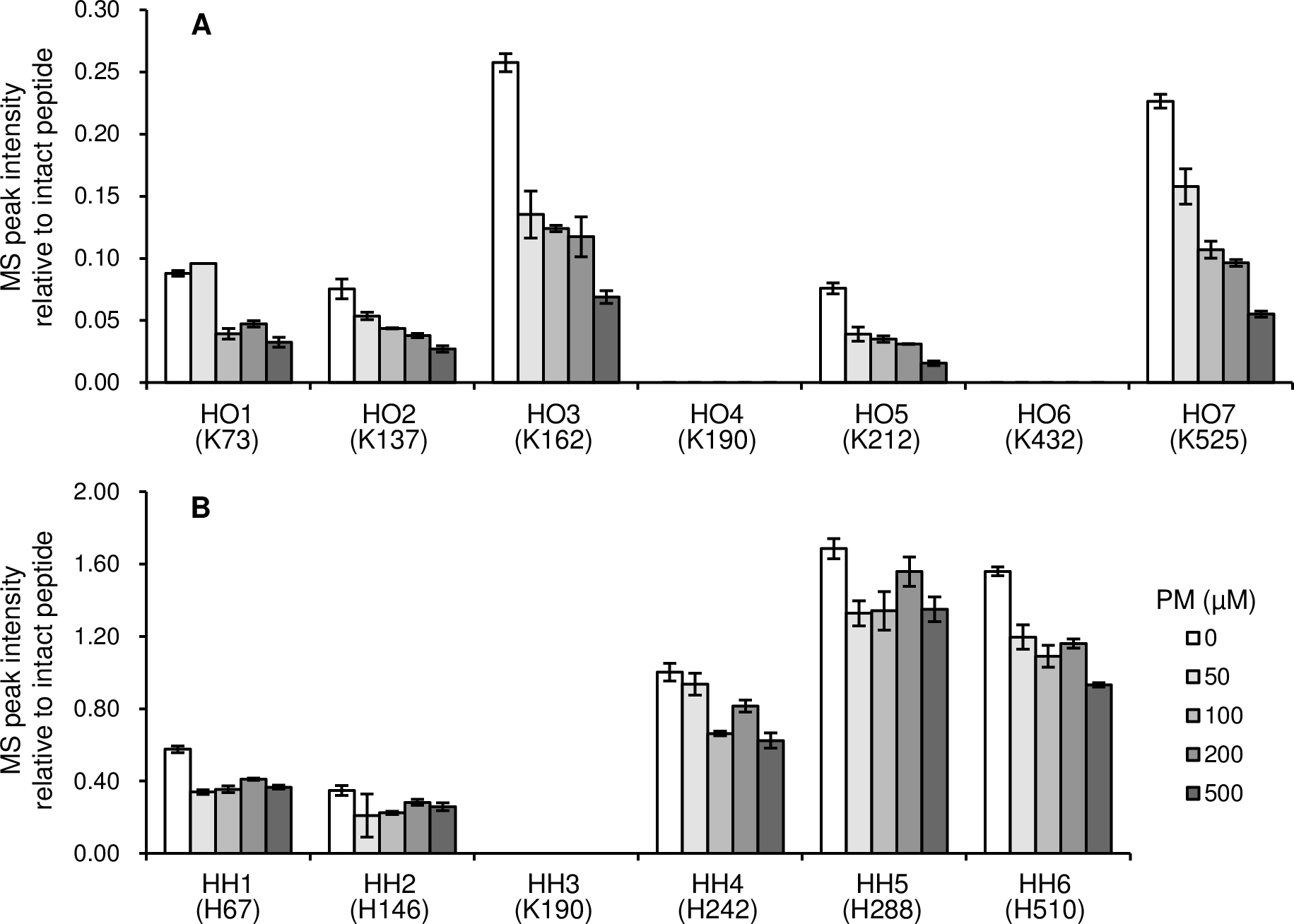

Supplement: S11 Fig — Changes in levels (as a relative intensity to corresponding intact peptide) of (A) ONE-modified HSA peptides and (B) HNE-modified HSA peptides in the reactions between HSA and 13-HOPDE in the presence of AscA and increasing concentrations of PM. The information in parentheses indicates modification site. Data are presented as means ± SEM (error bars) from triplicate samples except for HO1 at 50 μM PM (from single sample). (TIF) [file pone.0196050.s011.tif]
