# Supplementary material for: Inhibition effect of pyridoxamine on lipid hydroperoxide-derived modifications to human serum albumin
Source: PLoS One. 2018 Apr 19;13(4):e0196050. doi: 10.1371/journal.pone.0196050 (PMC5908094; doi:10.1371/journal.pone.0196050)
Supplement: S10 Fig — (PDF) [file pone.0196050.s010.pdf]

## A$^{21}$LVLIAFAQYLQQCPFEDH$^{39}$ (ONE) VK$^{41}$

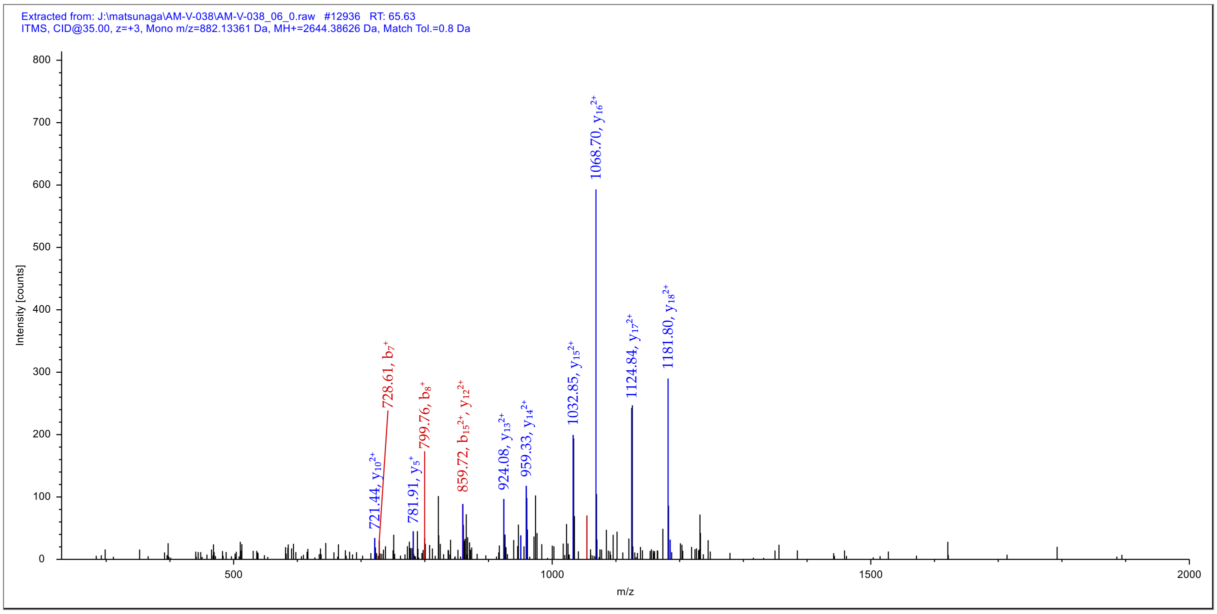


## A$^{21}$LVLIAFAQYLQQCPFEDH$^{39}$ (HNE) VK$^{41}$

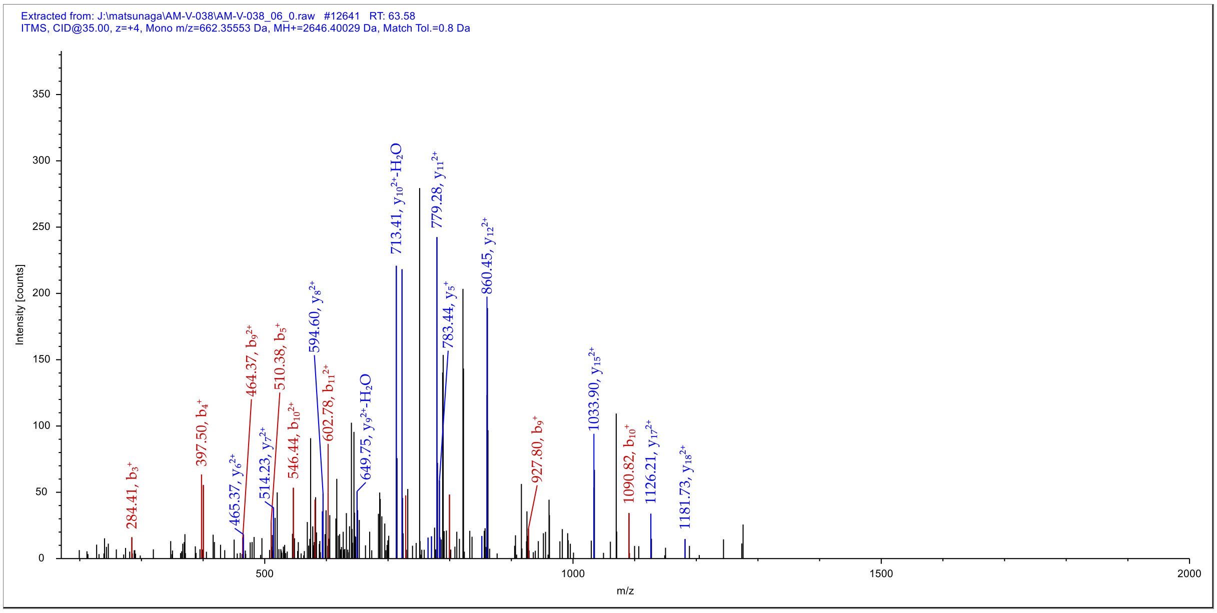


**S10 Fig. MS/MS spectra of ONE- and HNE-modified HSA peptides listed in Table 1.**

## $S^{65}LH^{67}$ (HNE) $TLFGDK^{73}$

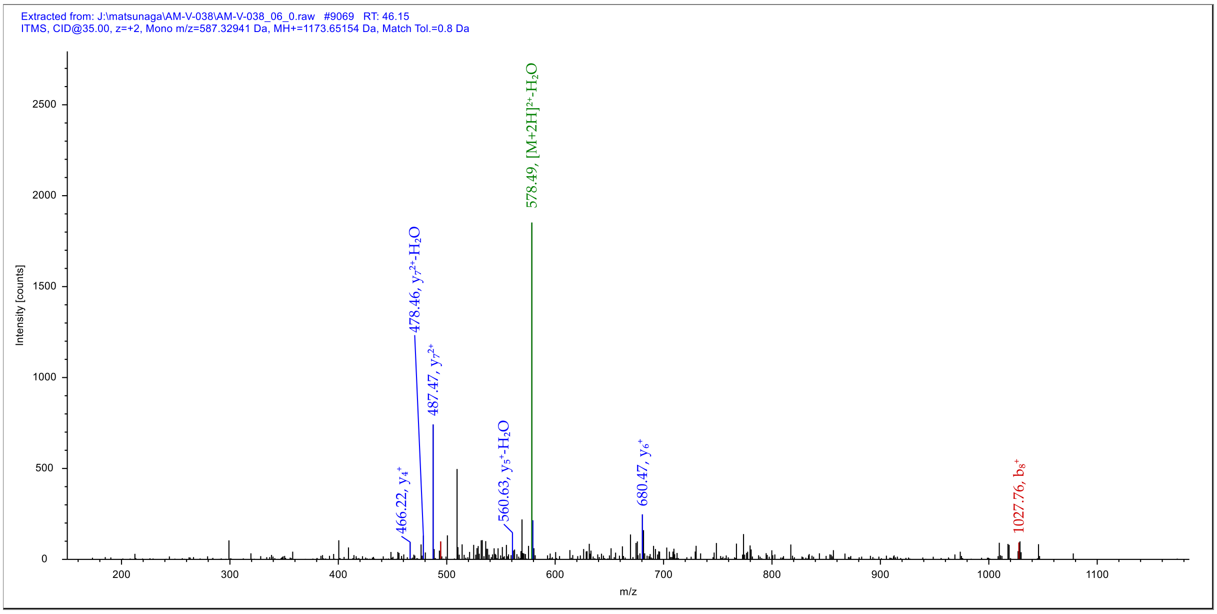


## $S^{65}LH^{67}$ (HNE) $TLFGDKLCTVATLR^{81}$

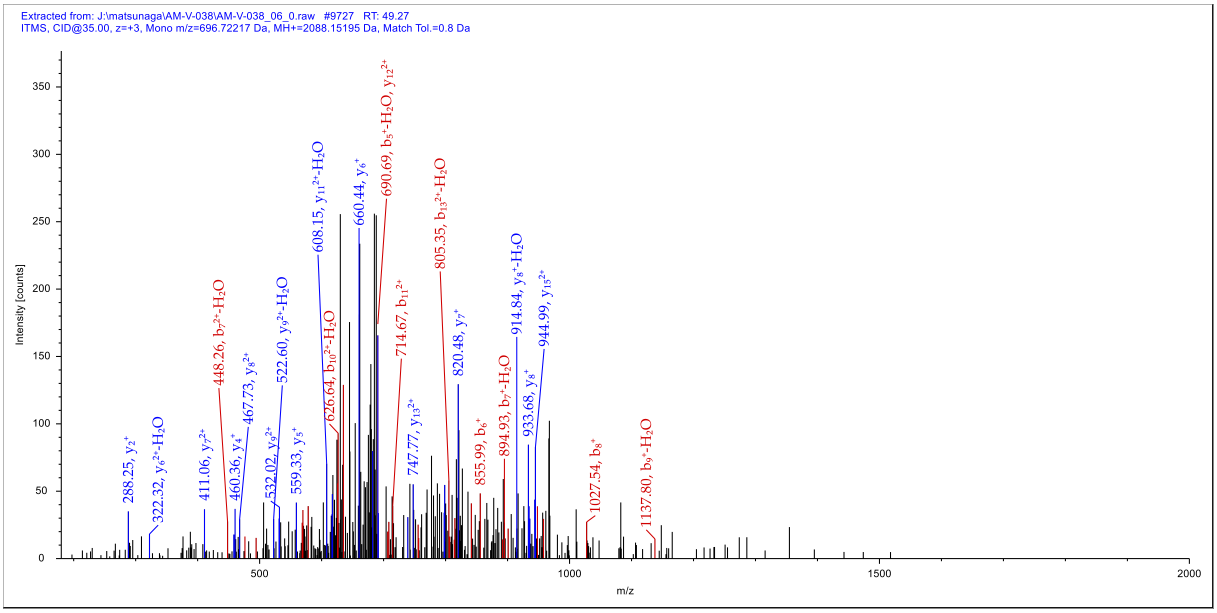


**S10 Fig. Continued.**

S[65]LHTLFGDK[73] (ONE) LCTVATLR[81]

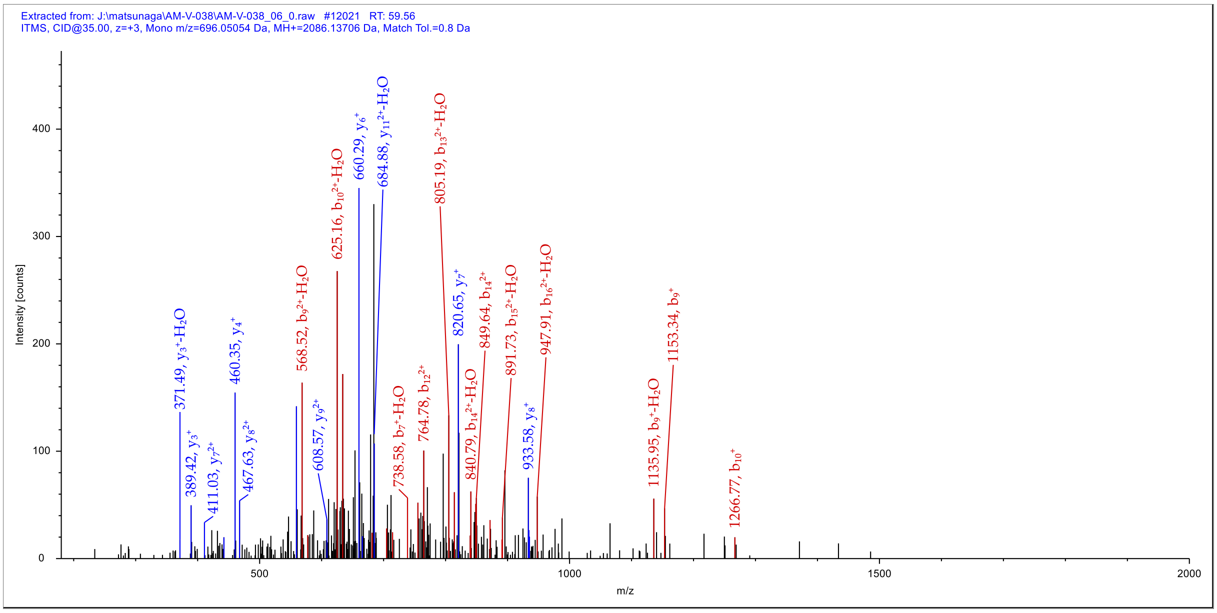


S[65]LHTLFGDK[73] (ONE−$H_2O$) LCTVATLR[81]

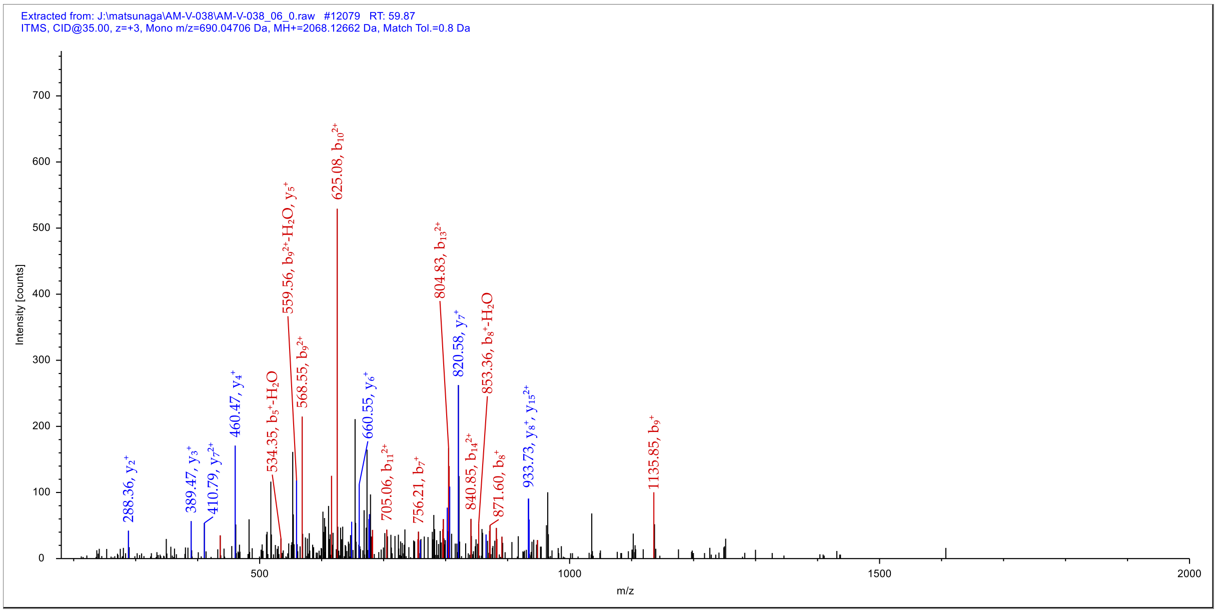


**S10 Fig. Continued.**

## L$^{115}$VRPEVDVMCTAFHDNEETFLK$^{136}$ (ONE−$H_2O$) K$^{137}$

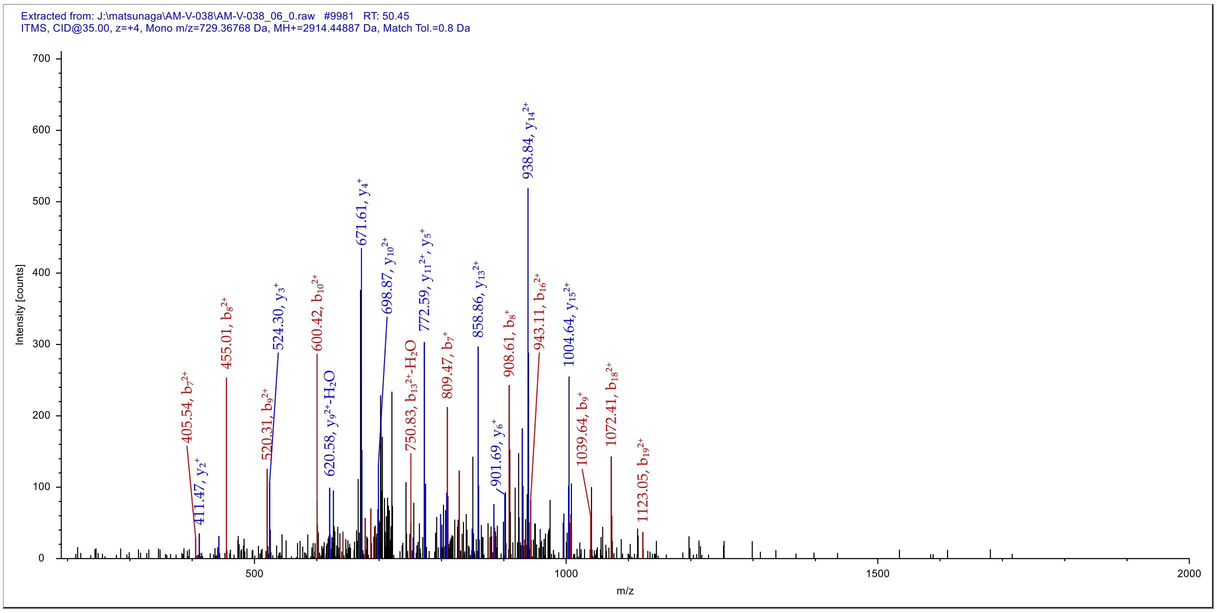


## K$^{137}$ (ONE−$H_2O$) YLYEIAR$^{144}$

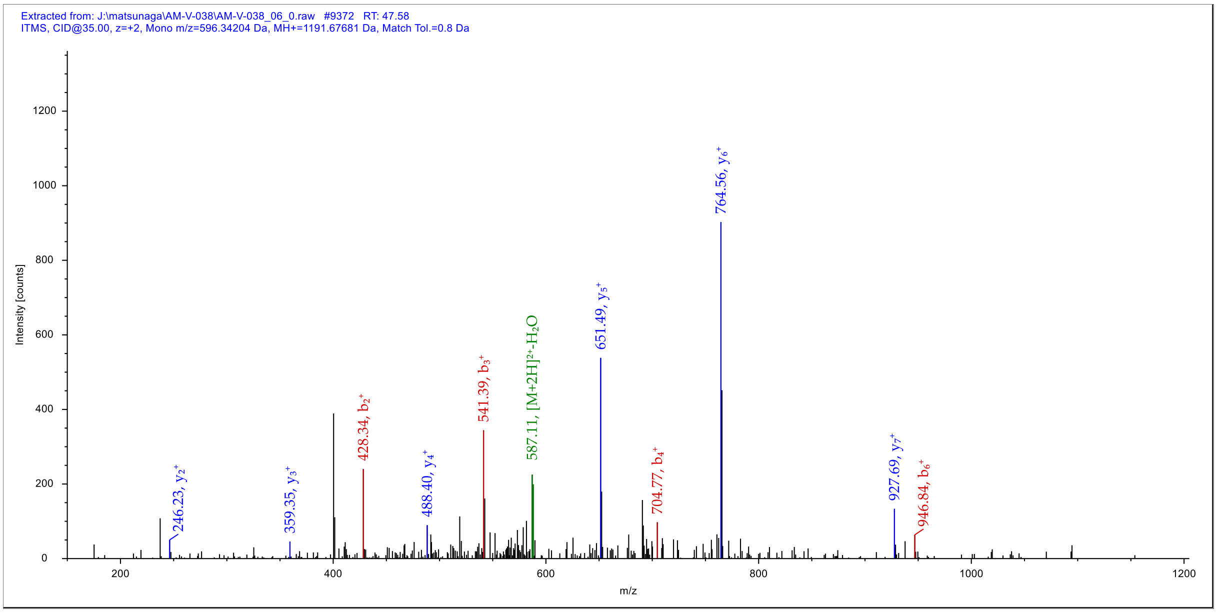


**S10 Fig. Continued.**

## $R^{145}$ (ONE) $H^{146}$ (HNE) $PYFYAPELLFFAK^{159}$

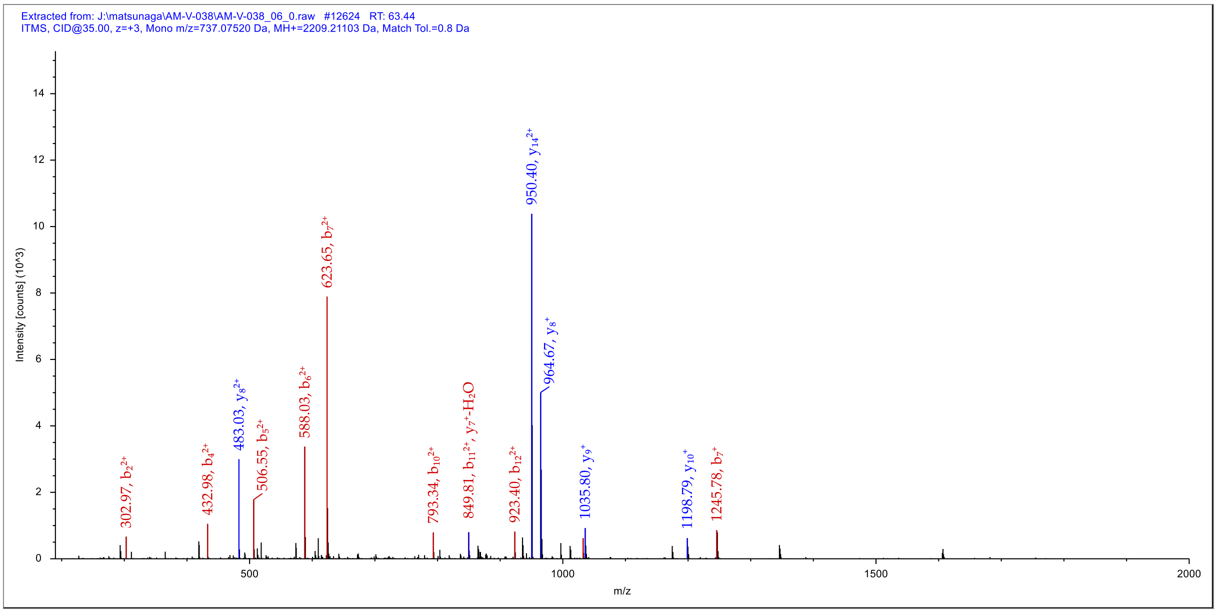


## $H^{146}$ (HNE) $PYFYAPELLFFAK^{159}$

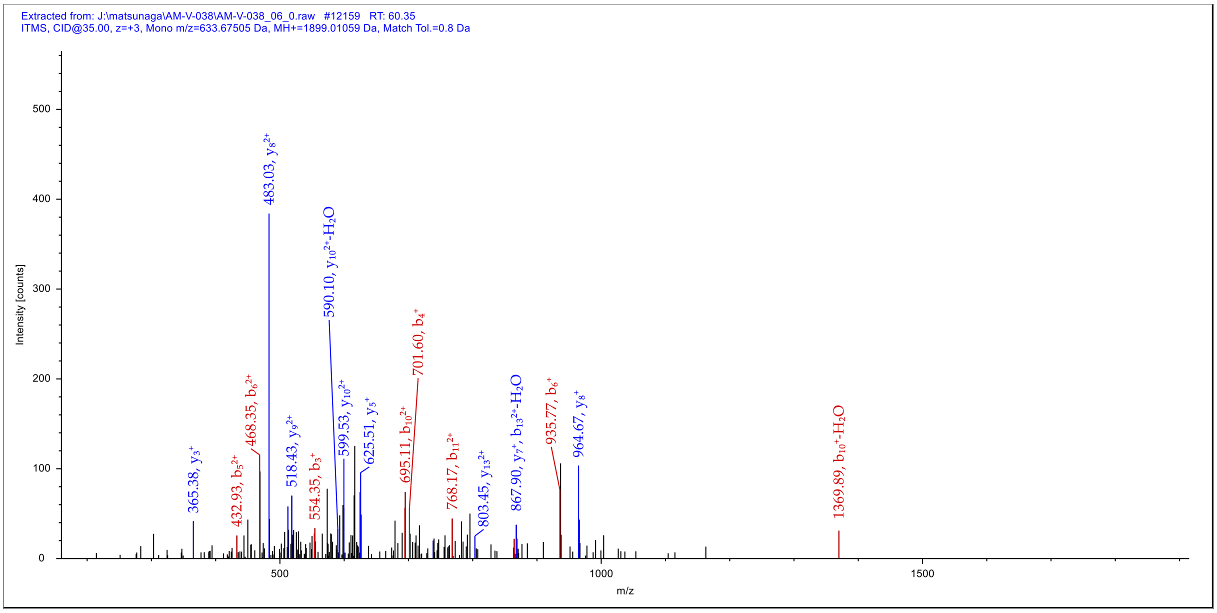


**S10 Fig. Continued.**

## $R^{145}H^{146}$ (ONE) PYFYAPELLFFAK$^{159}$

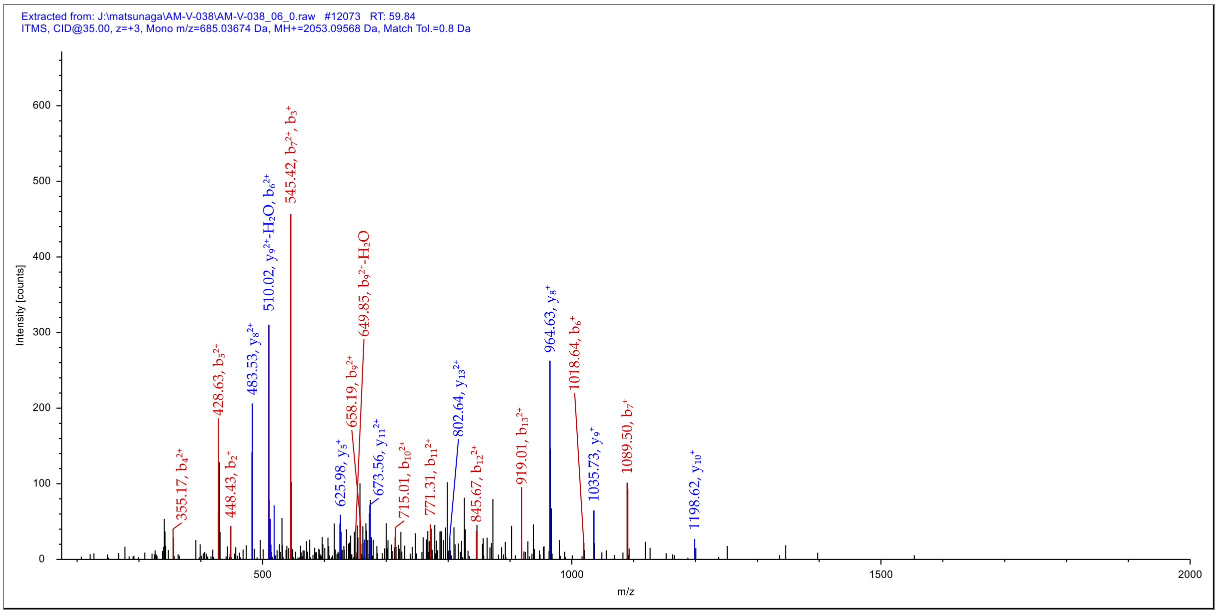


## $R^{145}H^{146}$ (HNE) PYFYAPELLFFAK$^{159}$

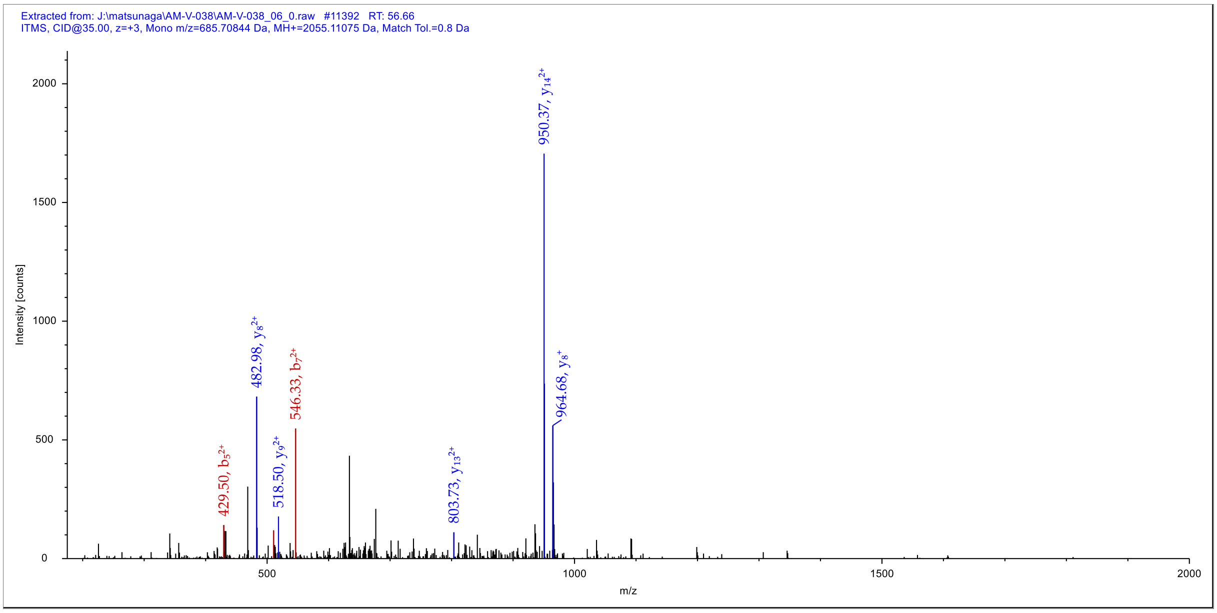


**S10 Fig. Continued.**

## R$^{160}$YK$^{162}$ (ONE) AAFTECCQAADK$^{174}$

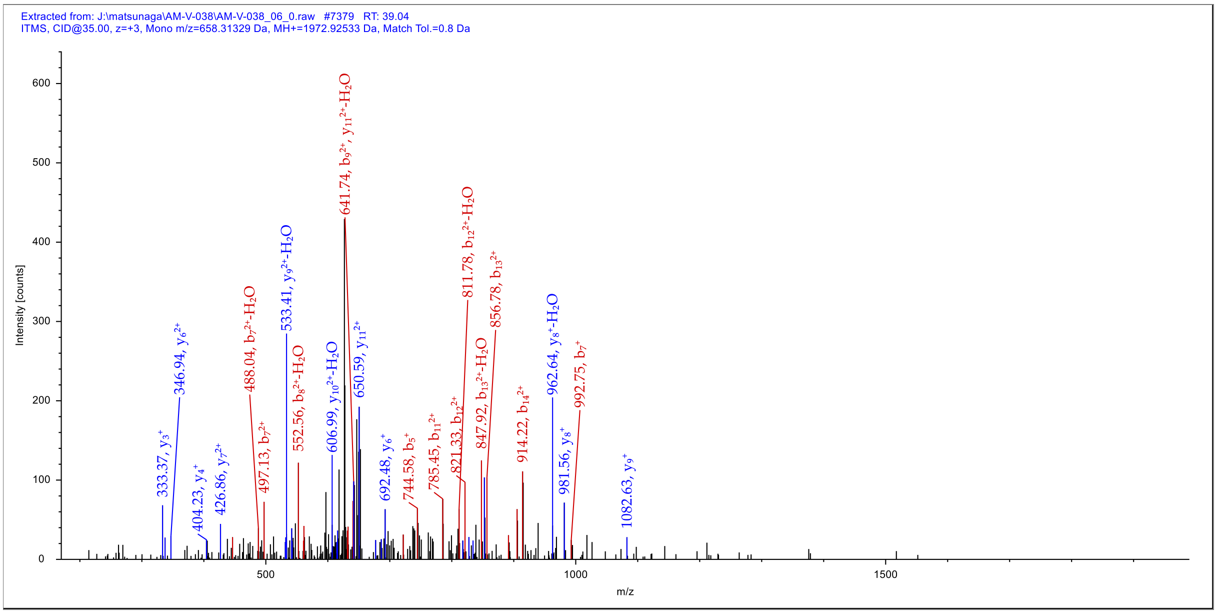


## R$^{160}$YK$^{162}$ (ONE−$H_2O$) AAFTECCQAADK$^{174}$

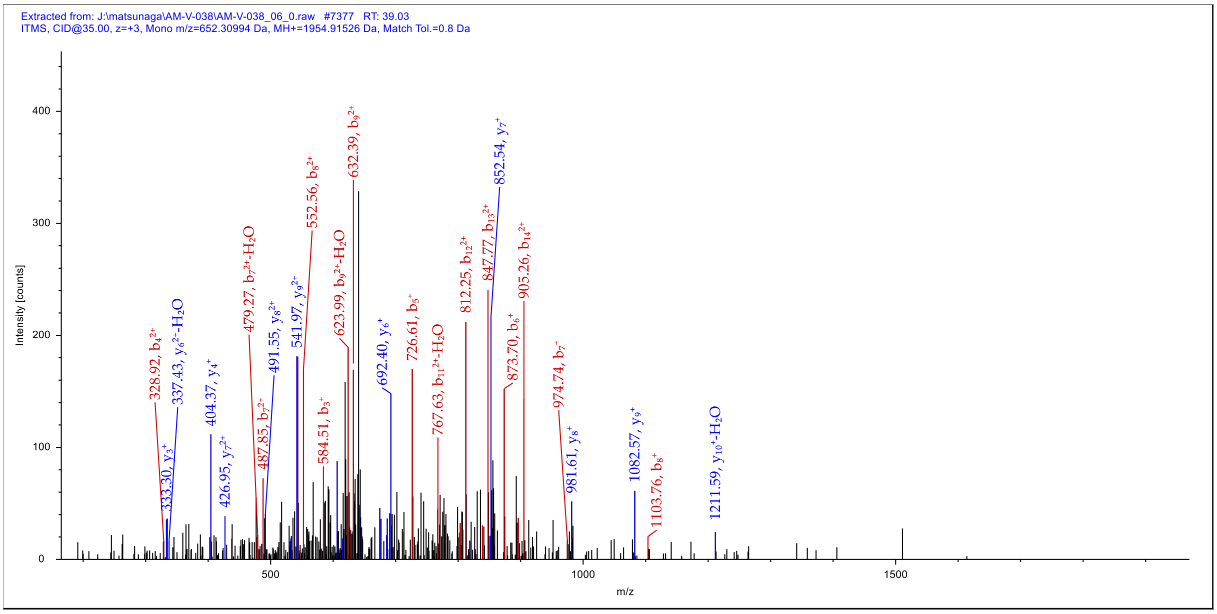


**S10 Fig. Continued.**

## $Y^{161}K^{162}$ (ONE) AAFTECCQAADK$^{174}$

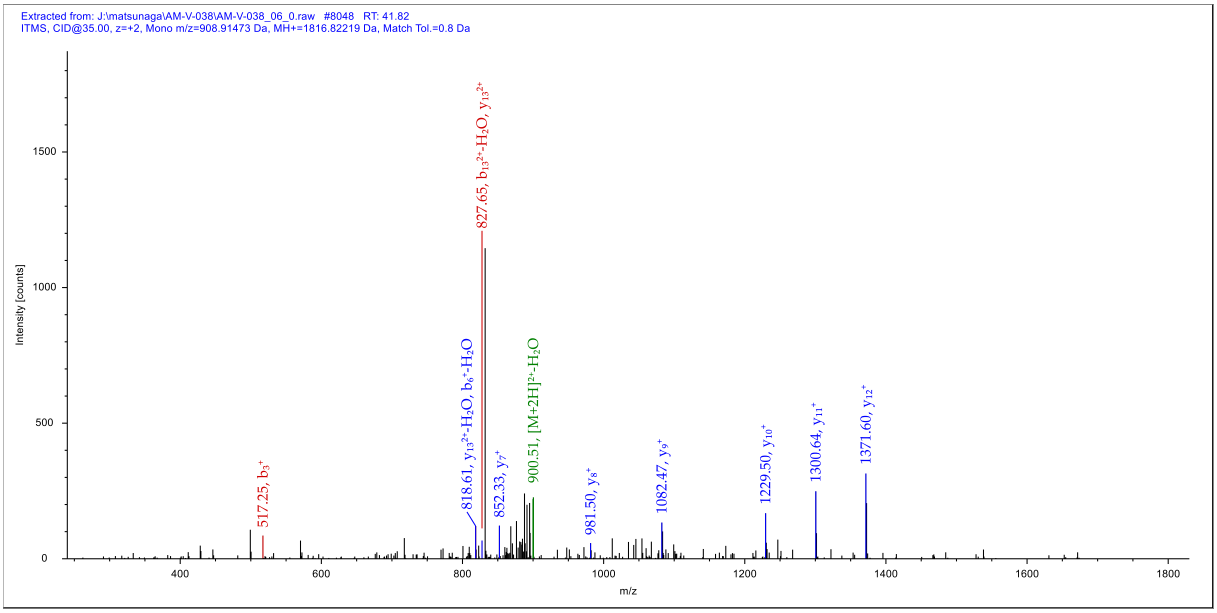


## $Y^{161}K^{162}$ (ONE−$H_2O$) AAFTECCQAADK$^{174}$

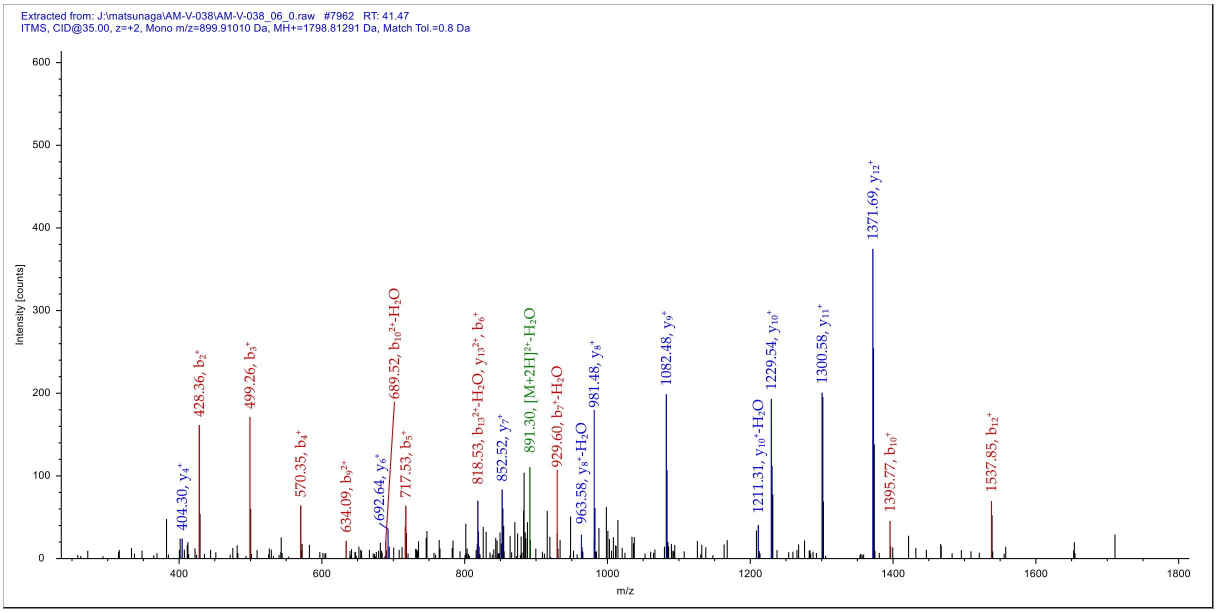


**S10 Fig. Continued.**

## L[182]DELRDEGK[190] (ONE−$H_2O$) ASSAK[195]

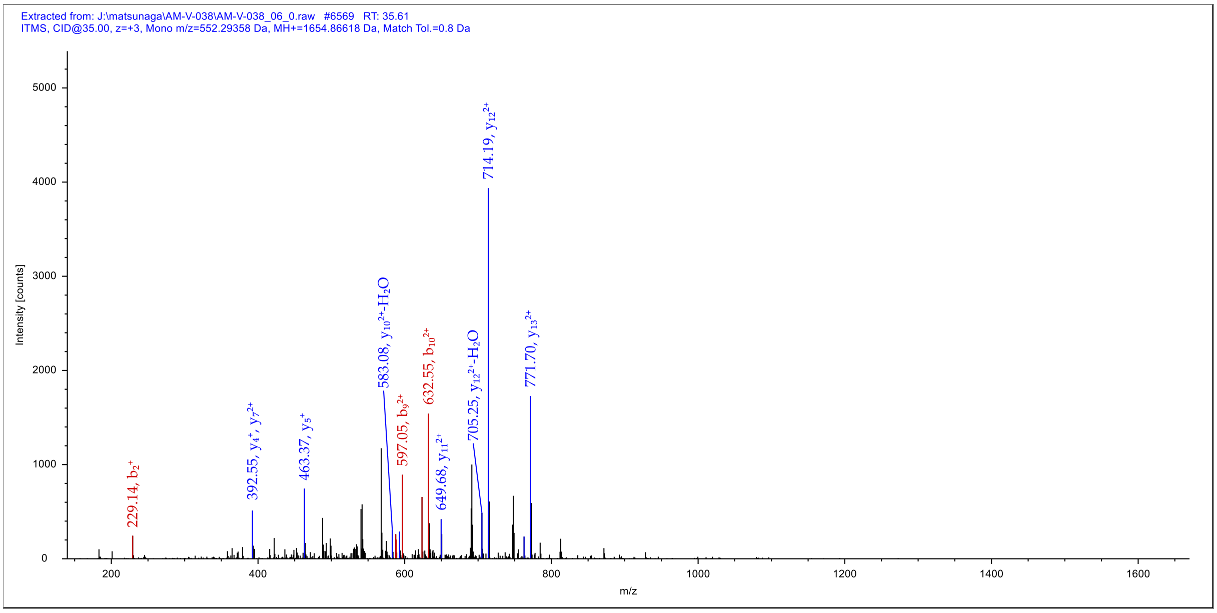


## L[182]DELRDEGK[190] (HNE−2$H_2O$) ASSAK[195]

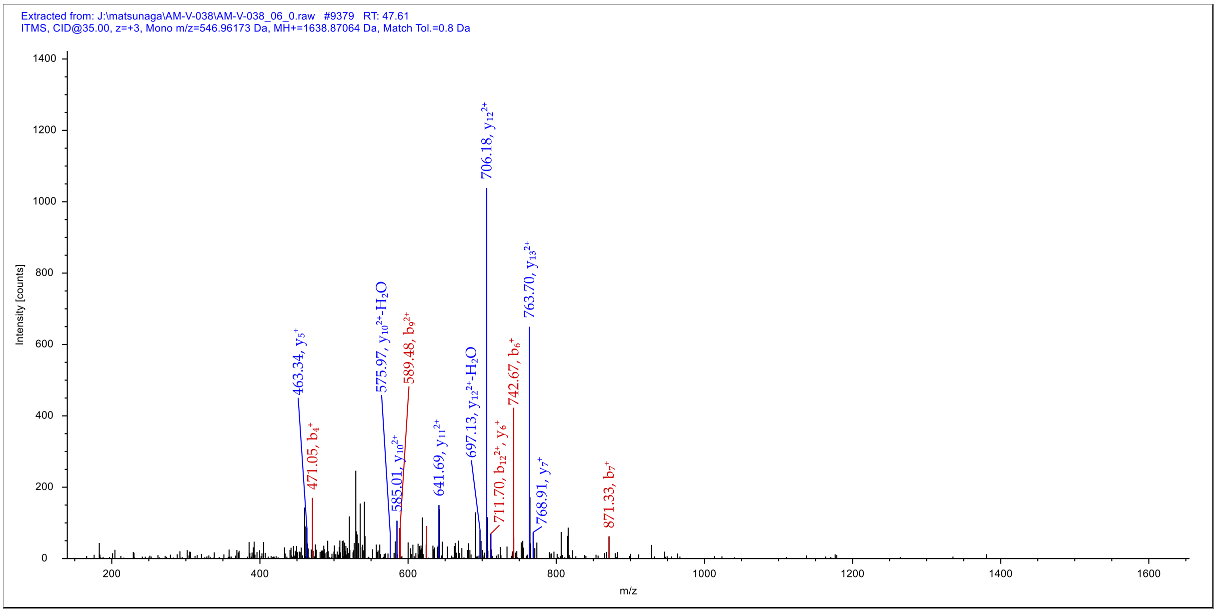


**S10 Fig. Continued.**

## C[200]ASLQK[205] (ONE−$H_2O$) FGER[209]

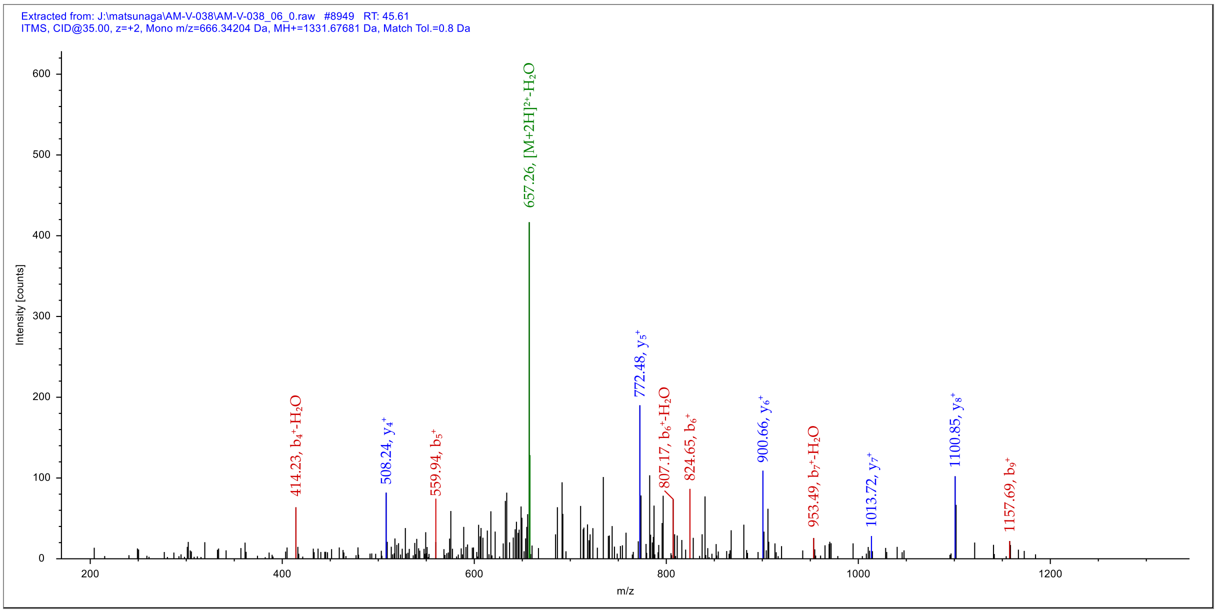


## C[200]ASLQK[205] (HNE−2$H_2O$) FGER[209]

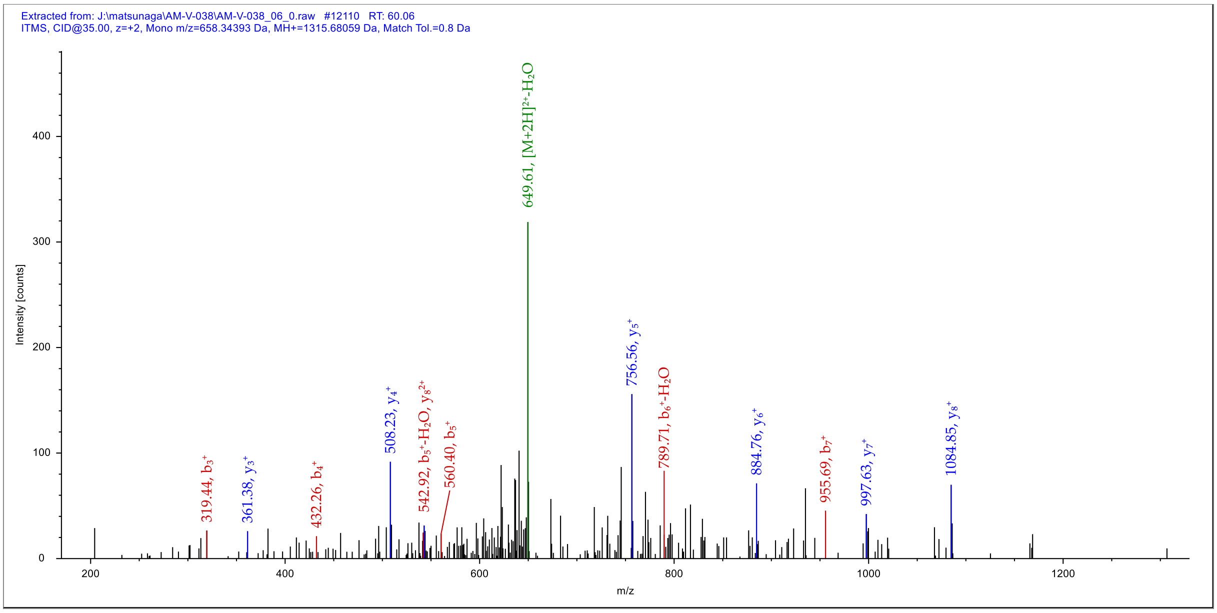


**S10 Fig. Continued.**

## $A^{210}FK^{212}$ (ONE−$H_2O$) $AWAVAR^{218}$

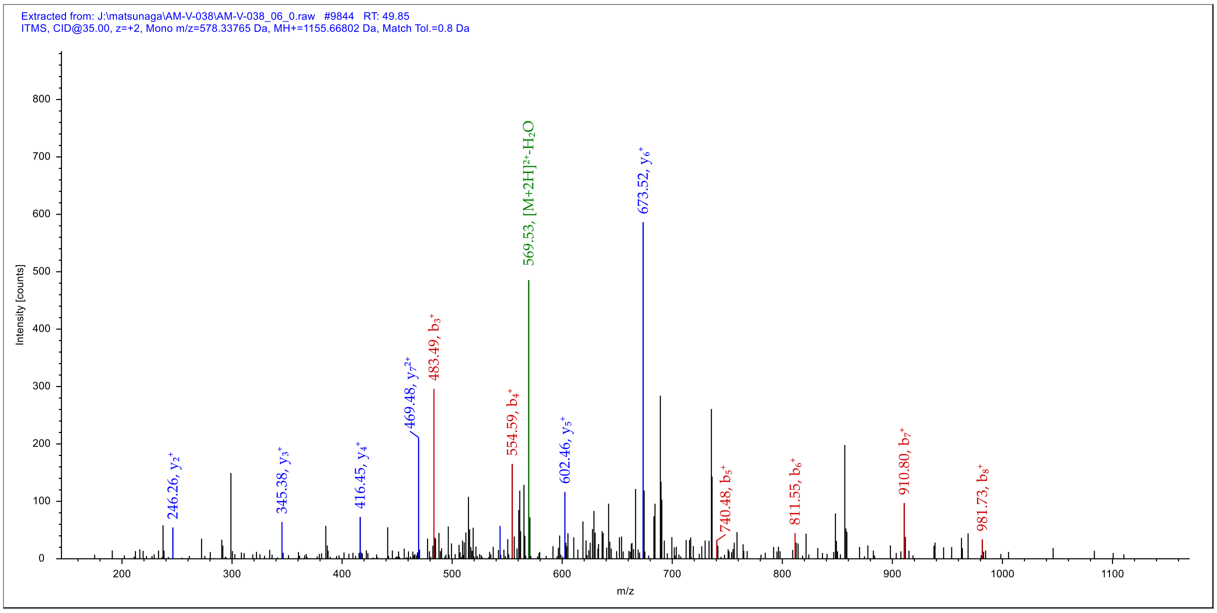


## $V^{241}H^{242}$ (HNE) $TECCHGDLLECADDR^{257}$

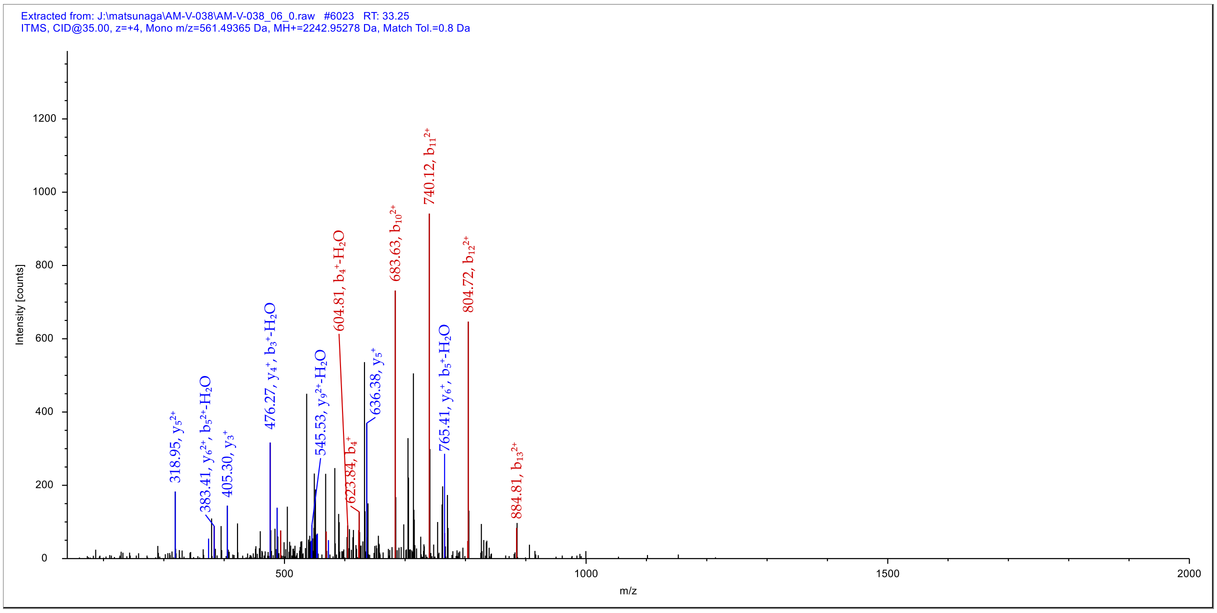


**S10 Fig. Continued.**

## V[241]H[242] (HNE) TECCHGDLLECADDRADLAK[262]

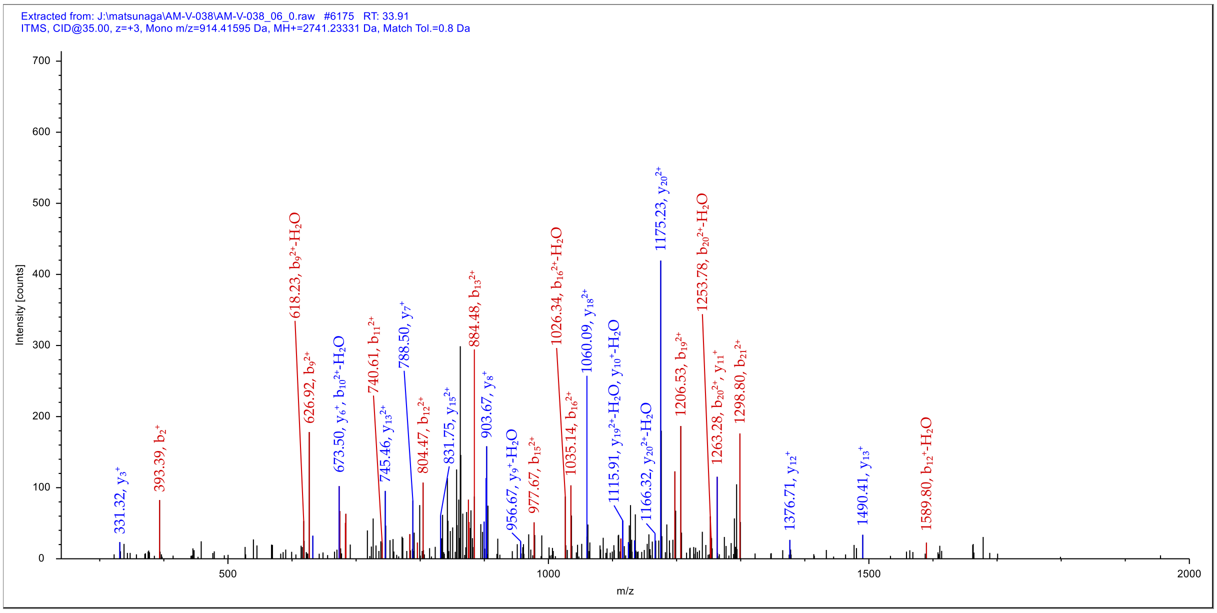


## V[241]H[242] (HNE) TECCH[247] (ONE) GDLLECADDR[257]

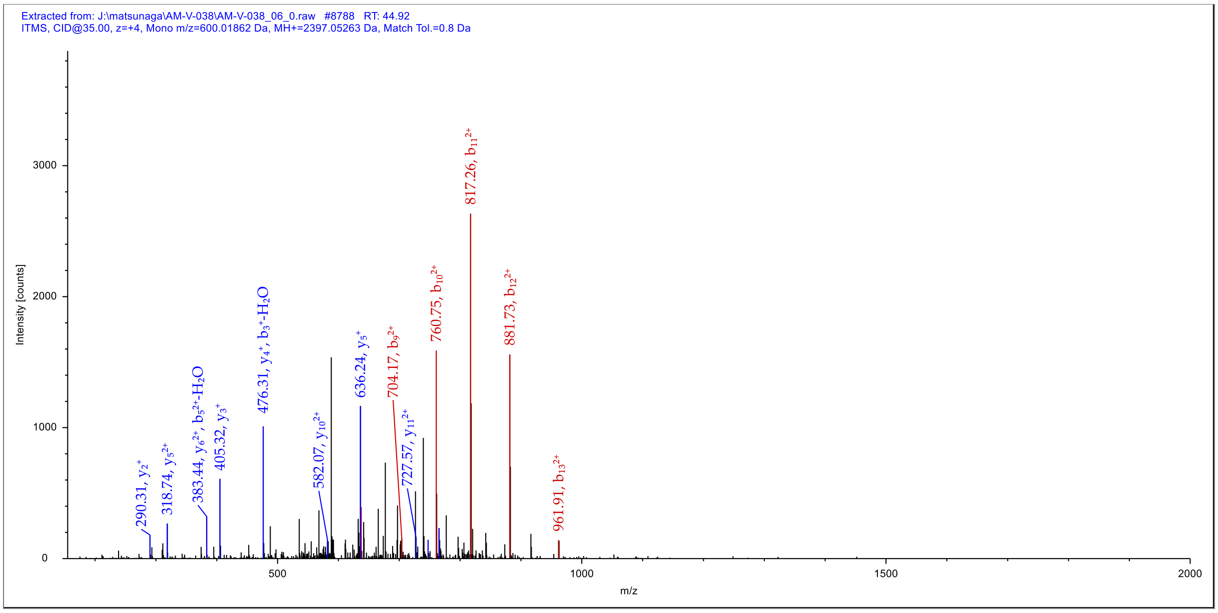


**S10 Fig. Continued.**

## $V^{241}H^{242}$ (HNE) $TECCH^{247}$ (HNE) $GDLLECADDR^{257}$

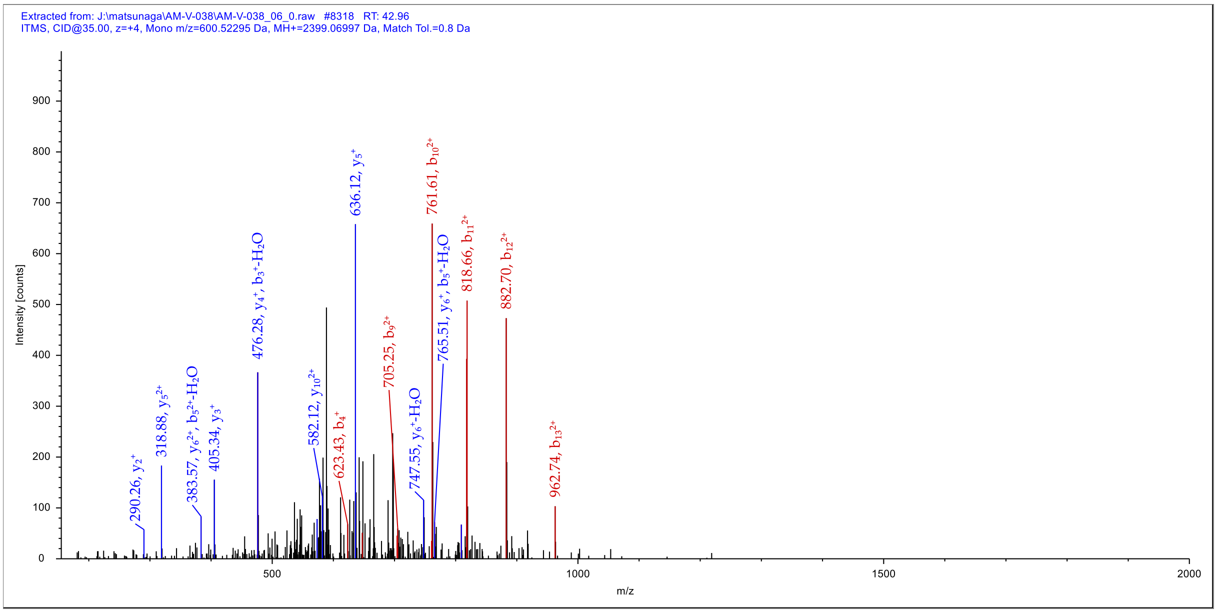


## $V^{241}H^{242}$ (HNE) $TECCH^{247}$ (HNE) $GDLLECADDRADLAK^{262}$

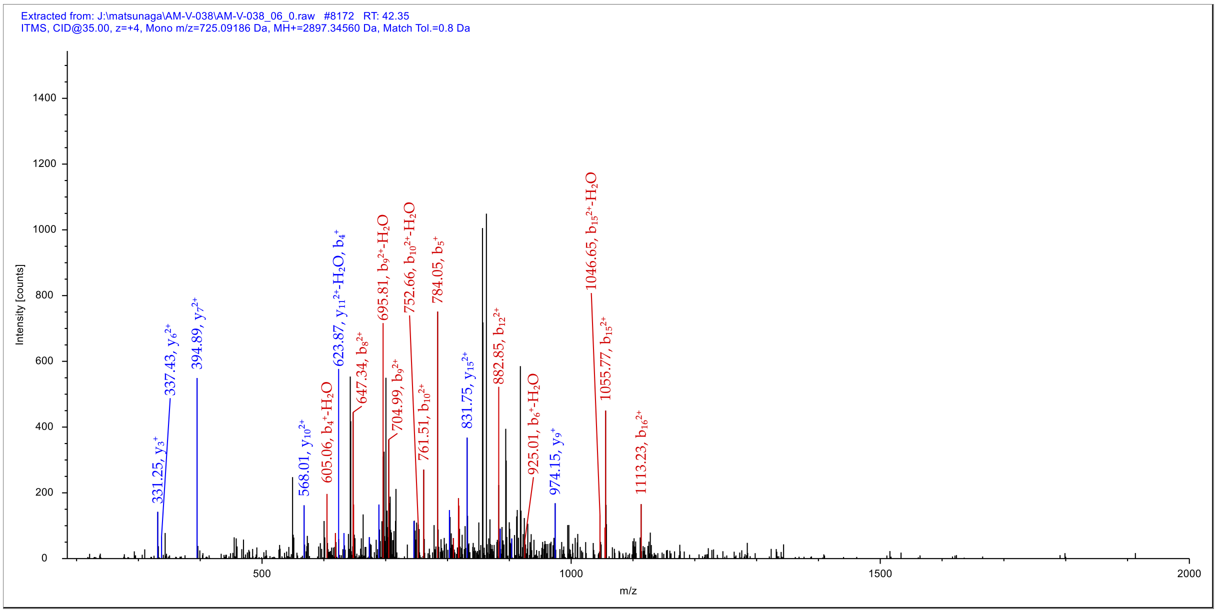


**S10 Fig. Continued.**

## $S^{287}H^{288}$ (HNE) CIAEVENDEMPADLPSLAADFVESK$^{313}$

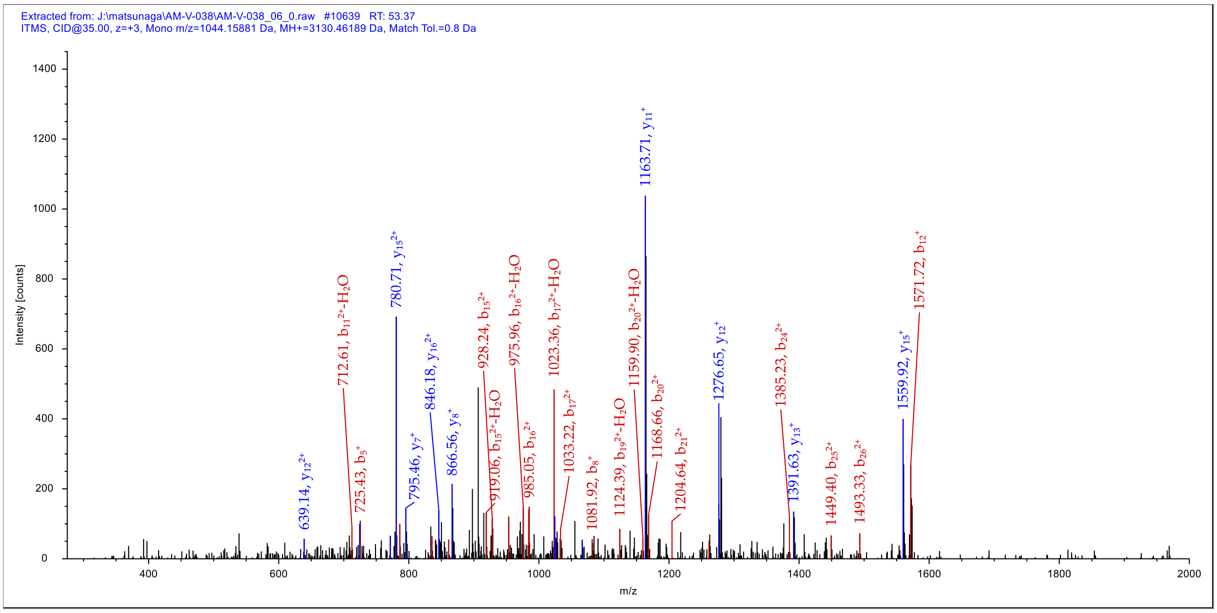


## $R^{337}$ (ONE) $H^{338}$ (HNE) PDYSVVLLLR$^{348}$

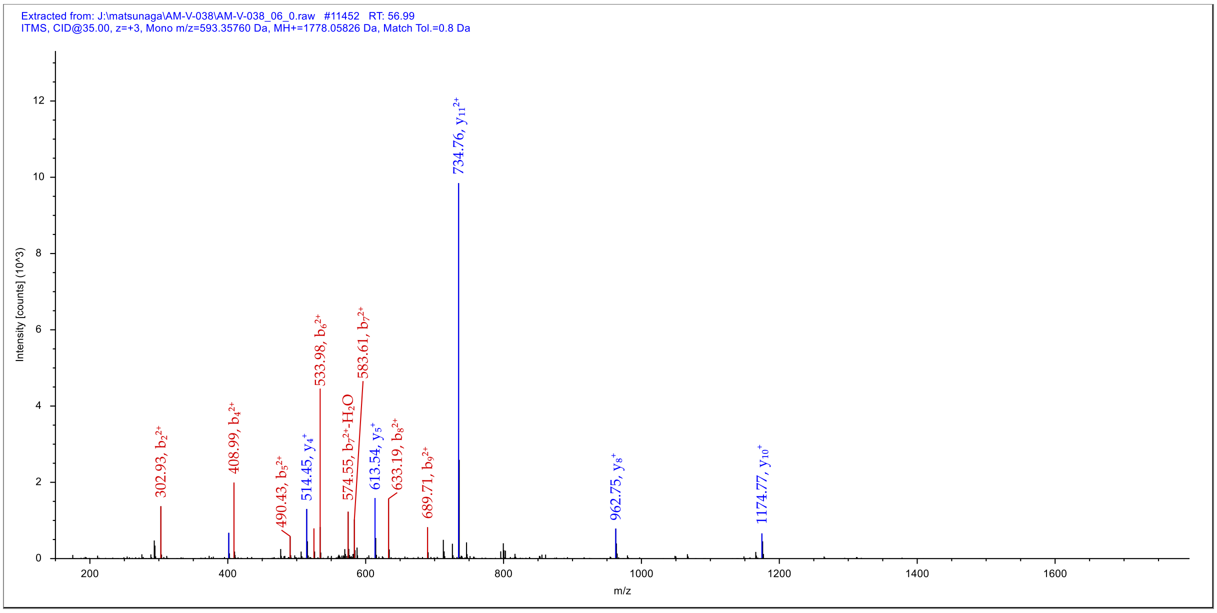


**S10 Fig. Continued.**

## $R^{337}H^{338}$ (HNE) $PDYSVVLLLR^{348}$

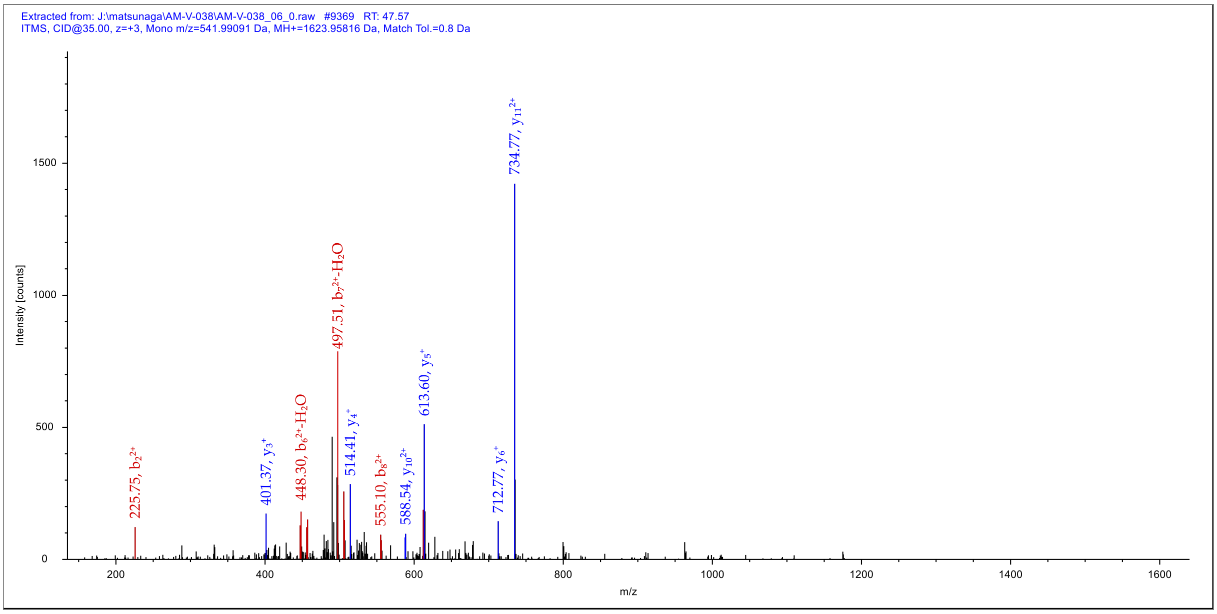


## $V^{373}FDEFK^{378}$ (ONE) $PLVEEPQNLIK^{389}$

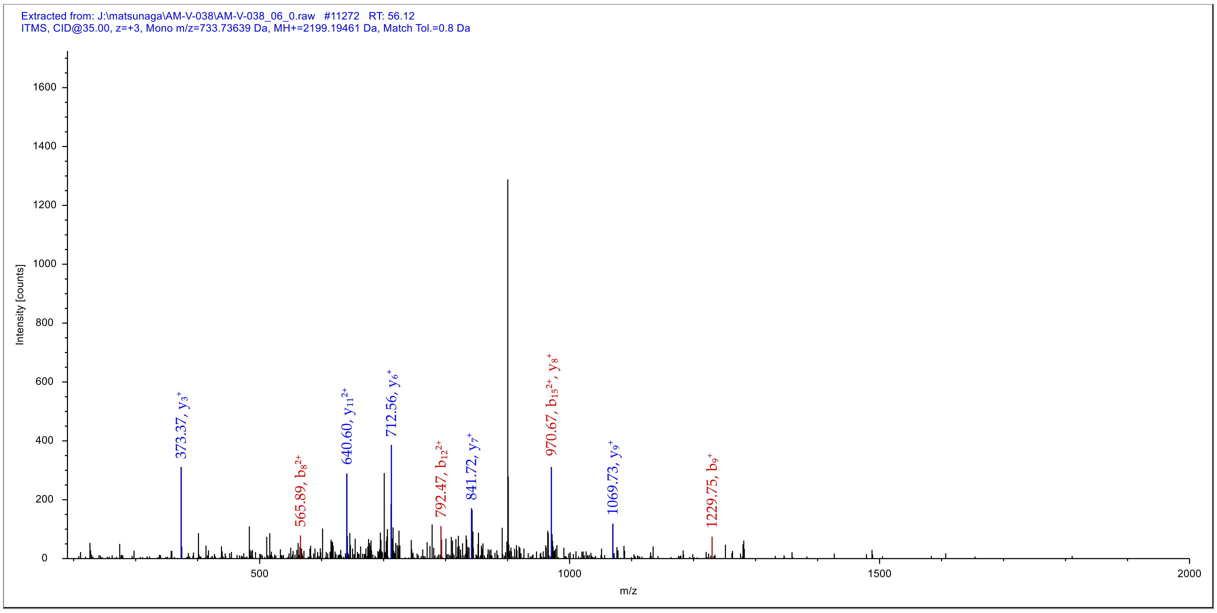


**S10 Fig. Continued.**

N[429]LGK[432] (ONE) VGSK[436]

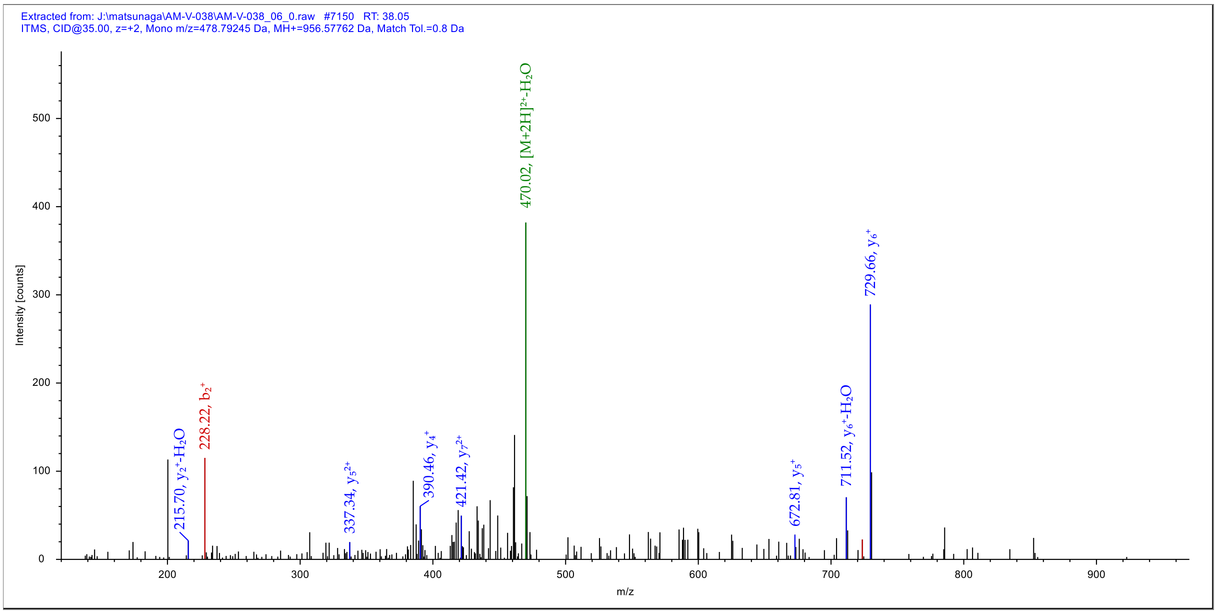


E[501]FNAETFTFH[510] (HNE) ADICTLSEK[519]

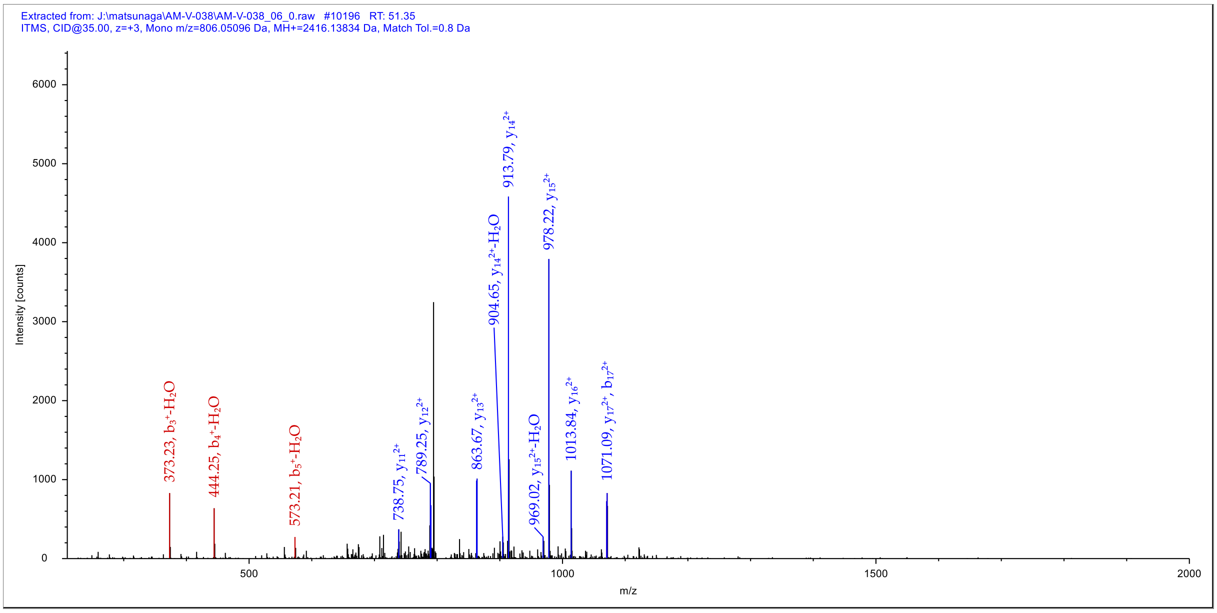


**S10 Fig. Continued.**

$K^{525}$ (ONE−$H_2O$) QTALVELVK$^{534}$

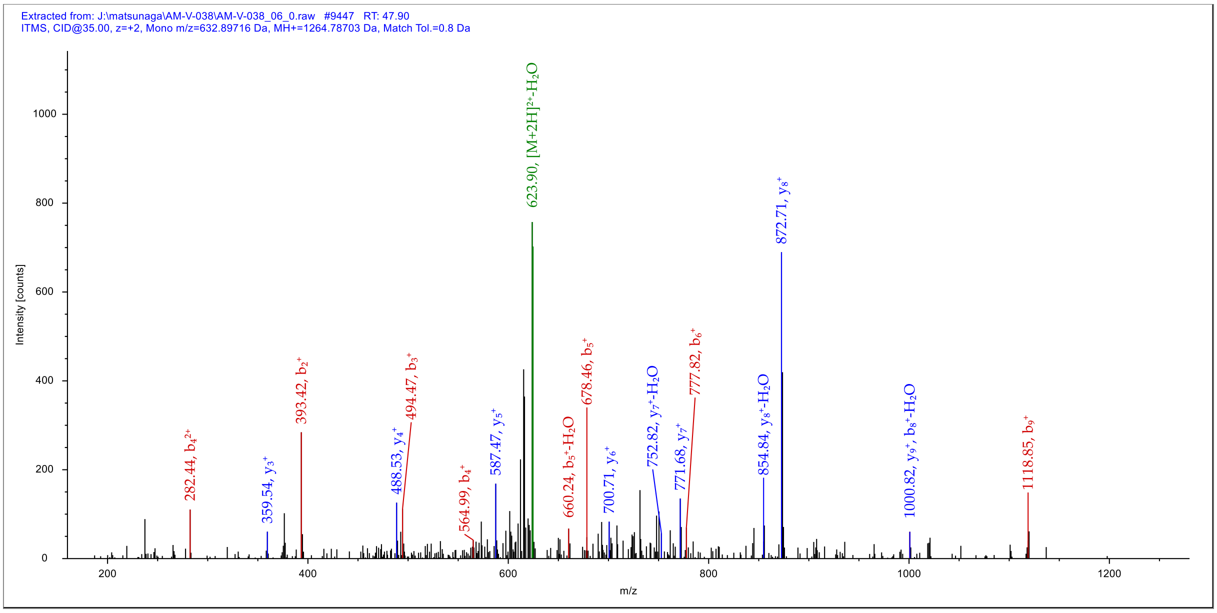


**S10 Fig. Continued.**
