# Supplementary material for: Inhibition effect of pyridoxamine on lipid hydroperoxide-derived modifications to human serum albumin
Source: PLoS One. 2018 Apr 19;13(4):e0196050. doi: 10.1371/journal.pone.0196050 (PMC5908094; doi:10.1371/journal.pone.0196050)
Supplement: S1 Table — * indicates a modification site. O indicates a peptide identified automatically. Δ indicates a peptide identified manually. (PDF) [file pone.0196050.s012.pdf]

S1 Table. List of ONE-modified HSA peptides identified from the reaction between HSA (30 μM) and ONE (300 μM).

Peptide Sequence	Modification		ONE 30 μM	ONE 3 μM
	Site	Type		
DAH*KSEVAHR	H3	ONE–H_2O		
DLGEENFK*ALVLIAFAQYLQQCPFEDHVK	K20	ONE–H_2O		
FKDLGEENFK*ALVLIAFAQYLQQCPFEDHVK	K20	ONE, ONE–H_2O		
ALVLIAFAQYLQQCPFEDHVK*LVNEVTEFAK	K41	ONE, ONE–H_2O		
LVNEVTEFAK*TCVADESAENCDK	K51	ONE–H_2O		
TCVADESAENCDK*SLHTLFGDK	K64	ONE, ONE–H_2O		
TCVADESAENCDKSLH*TLFGDKLCTVATLR	H67	ONE–H_2O		
SLHTLFGDK*LCTVATLR	K73	ONE, ONE–H_2O	O	
NECFLQH*KDDNPNLPR	H105	ONE–H_2O		
LVR*PEVDVMCTAFHDNEETFLK	R117	ONE–H_2O	O	
LVR*PEVDVMCTAFHDNEETFLKK	R117	ONE–H_2O	O	
LVR*PEVDVMCTAFHDNEETFLK*K	R117/K136	ONE–H_2O/ONE, ONE–H_2O		
LVRPEVDVMCTAFHDNEETFLK*K	K136	ONE		
K*YLYEIAR	K137	ONE–H_2O	O	Δ
R*HPYFYAPELLFFAK	R145	ONE–H_2O		
R*H*PYFYAPELLFFAK	R145/H146	ONE/ONE		
HPYFYAPELLFFAK*R	K159	ONE, ONE–H_2O		
RHPYFYAPELLFFAK*R	K159	ONE, ONE–H_2O		
R*YK*AAFTECCQAADK	R159/K162	ONE–H_2O/ONE, ONE–H_2O		
YK*AAFTECCQAADK	K162	ONE, ONE–H_2O	O	
RYK*AAFTECCQAADK	K162	ONE, ONE–H_2O	O	
LDELR*DEGK	R186	ONE–H_2O	O	O
LDELRDEGK*ASSAK	K190	ONE–H_2O		
ASSAK*QR	K195	ONE, ONE–H_2O	O	O
LK*CASLQK	K199	ONE–H_2O	O	O
FGER*AFK	R209	ONE–H_2O		
AFK*AWAVAR	K212	ONE, ONE–H_2O	O	O
AWAVAR*LSQR	R218	ONE–H_2O		
AEFAEVSK*LVTDLTK	K233	ONE, ONE–H_2O		
VH*TECCHGDLLECADDR	H242	ONE–H_2O		
VH*TECCHGDLLECADDRADLAK	H242	ONE–H_2O	O	
VH*TECCH*GDLLECADDRADLAK	H242/H247	ONE/ONE		
VHTECCHGDLLECADDR*ADLAK	R257	ONE		
LKECCEK*PLLEK	K281	ONE–H_2O		
DVCK*NYAEAK	K317	ONE–H_2O		
NYAEAK*DVFLGMFLYEYAR	K323	ONE		
LAK*TYETTLEK	K351	ONE, ONE–H_2O	O	Δ
TYETTLEK*CCAAADPHECYAK	K359	ONE–H_2O		

S1 Table. Continued.

Peptide Sequence	Modification		ONE 30 μM	ONE 3 μM
	Site	Type		
VFDEFK*PLVEEPQNLIK	K378	ONE–H_2O		
QNCELFEQLGEYK*FQNALLVR	K402	ONE–H_2O	O	
QNCELFEQLGEYK*FQNALLVR*YTK	K402/R410	ONE–H_2O/ONE–H_2O	O	Δ
FQNALLVR*YTK	R410	ONE–H_2O	O	O
QNCELFEQLGEYKFQNALLVR*YTK	R410	ONE–H_2O		
K*VPQVSTPTLVEVSR	K414	ONE, ONE–H_2O	O	Δ
NLGK*VGSK	K432	ONE, ONE–H_2O	O	O
VGSK*CCK	K436	ONE, ONE–H_2O	O	Δ
VGSK*CCKHPEAK	K436	ONE–H_2O		
VTK*CCTESLVNR	K475	ONE, ONE–H_2O		
R*PCFSALEVDETYVPK	R485	ONE–H_2O		
EFNAETFTFHADICTLSEK*ER	K519	ONE, ONE–H_2O		
K*QTALVELVK	K525	ONE, ONE–H_2O	O	O
ATK*EQLK	K541	ONE–H_2O	O	Δ
ATK*EQLKAVMDDFAAFVEK	K541	ONE–H_2O		
ATK*EQLK*AVMDDFAAFVEK	K541/K545	ONE–H_2O/ONE–H_2O		
EQLK*AVMDDFAAFVEK	K545	ONE, ONE–H_2O	O	
AVMDDFAAFVEK*CCK	K557	ONE–H_2O		
ADDK*ETCFAEEGKK	K564	ONE–H_2O		

* indicates a modification site.

O indicates a peptide identified automatically.

Δ indicates a peptide identified manually.
