# Supplementary material for: Inhibition effect of pyridoxamine on lipid hydroperoxide-derived modifications to human serum albumin
Source: PLoS One. 2018 Apr 19;13(4):e0196050. doi: 10.1371/journal.pone.0196050 (PMC5908094; doi:10.1371/journal.pone.0196050)
Supplement: S2 Table — * indicates a modification site. O indicates a peptide identified automatically. Δ indicates a peptide identified manually. (PDF) [file pone.0196050.s013.pdf]

S2 Table. List of HNE-modified HSA peptides identified from the reaction between HSA (30 μM) and HNE (300 μM).

Peptide Sequence	Modification		HNE 30 μM	HNE 3 μM
	Site	Type		
DAH*KSEVAHR	H3	HNE−H_2O		
DAH*KSEVAH*R	H3/H9	HNE, HNE−H_2O/HNE		
DAHK*SEVAHR	K4	HNE		
SEVAH*RFKDLGEENFK	H9	HNE, HNE−H_2O		
FK*DLGEENFK	K12	HNE, HNE−2H_2O		
FK*DLGEENFKALVLIAFAQYLQQCPFEDHVK	K12	HNE−H_2O		
FKDLGEENFK*ALVLIAFAQYLQQCPFEDHVK	K20	HNE		
ALVLIAFAQYLQQCPFEDH*VK	H39	HNE	O	
ALVLIAFAQYLQQCPFEDH*VKLVNEVTEFAK	H39	HNE−H_2O		
ALVLIAFAQYLQQCPFEDHVK*LVNEVTEFAK	K41	HNE−2H_2O		
TCVADESAENCDK*SLHTLFGDK	K64	HNE−H_2O		
TCVADESAENCDK*SLHTLFGDKLCTVATLR	K64	HNE		
SLH*TLFGDK	H67	HNE	O	Δ
SLH*TLFGDKLCTVATLR	H67	HNE, HNE−H_2O	O	
TCVADESAENCDKSLH*TLFGDK	H67	HNE		
TCVADESAENCDKSLH*TLFGDKLCTVATLR	H67	HNE−H_2O		
SLHTLFGDK*LCTVATLR	K73	HNE−2H_2O		
NECFLQH*KDDNPNLPR	H105	HNE		
QEPERNECFLQH*KDDNPNLPR	H105	HNE, HNE−H_2O		
QEPERNECFLQH*K*DDNPNLPR	H105/K106	HNE/HNE−2H_2O		
NECFLQHK*DDNPNLPR	K106	HNE−H_2O		
QEPERNECFLQHK*DDNPNLPR	K106	HNE−2H_2O		
LVRPEVDVMCTAFH*DNEETFLK	H128	HNE		
LVRPEVDVMCTAFH*DNEETFLKK	H128	HNE, HNE−H_2O		
LVRPEVDVMCTAFHDNEETFLK*K	K136	HNE−2H_2O		
K*YLYEIAR	K137	HNE, HNE−H_2O, HNE−2H_2O		
H*PYFYAPELLFFAK	H146	HNE	O	Δ
RH*PYFYAPELLFFAK	H146	HNE	O	O
RH*PYFYAPELLFFAK*R	H146/K159	HNE/HNE−2H_2O		
RHPYFYAPELLFFAK*R	K159	HNE−2H_2O		
RYK*AAFTECCQAADK	K162	HNE, HNE−2H_2O		
YK*AAFTECCQAADK	K162	HNE, HNE−H_2O, HNE−2H_2O		
AAFTECCQAADK*AACLLPK	K174	HNE−2H_2O		
AACLLPK*LDELRDEGK	K181	HNE−2H_2O		
LDELRDEGK*ASSAK	K190	HNE−2H_2O	O	O
DEGK*ASSAK*QR	K190/K195	HNE−2H_2O/HNE−2H_2O		
ASSAK*QR	K195	HNE, HNE−2H_2O	O	Δ
LK*CASLQK	K199	HNE, HNE−H_2O, HNE−2H_2O	O	O

S2 Table. Continued.

Peptide Sequence	Modification		HNE 30 μM	HNE 3 μM
	Site	Type		
CASLQK*FGER	K205	HNE−2H_2O		
AFK*AWAVAR	K212	HNE, HNE−H_2O, HNE−2H_2O		
FPK*AEFAEVSK	K225	HNE		
AEFAEVSK*LVTDLTK	K233	HNE, HNE−H_2O		
VH*TECCHGDLLECADDR	H242	HNE		
VH*TECCHGDLLECADDRADLAK	H242	HNE	O	Δ
LVTDLTKVH*TECCHGDLLECADDRADLAK	H242	HNE−H_2O		
VH*TECCH*GDLLECADDR	H242/H247	HNE/HNE		
VH*TECCH*GDLLECADDRADLAK	H242/H247	HNE/HNE		
LK*ECCEKPLLEK	K276	HNE		
LK*ECCEKPLLEKSHCIAEVENDEMPADLPSLAADFVESK	K276	HNE		
LKECCEKPLLEK*SHCIAEVENDEMPADLPSLAADFVESK	K286	HNE−2H_2O		
SH*CIAEVENDEMPADLPSLAADFVESK	H288	HNE	O	Δ
SH*CIAEVENDEMPADLPSLAADFVESKDVCK	H288	HNE		
NYAEAK*DVFLGMFLYEYAR	K323	HNE		
DVCKNYAEAK*DVFLGMFLYEYAR	K323	HNE−H_2O		
H*PDYSVVLLLR	H338	HNE		
RH*PDYSVVLLLR	H338	HNE	O	O
LAK*TYETTLEK	K351	HNE, HNE−H_2O, HNE−2H_2O		
LAKTYETTLEK*CCAAADPHECYAK	K359	HNE−H_2O		
TYETTLEK*CCAAADPHECYAKVFDEFKPLVEEPQNLIK	K359	HNE−H_2O		
CCAAADPH*ECYAK	H367	HNE		
VFDEFK*PLVEEPQNLIK	K378	HNE, HNE−H_2O, HNE−2H_2O		
VFDEFKPLVEEPQNLIK*QNCELFEQLGEYK	K389	HNE−H_2O		
QNCELFEQLGEYK*FQNALLVR	K402	HNE, HNE−2H_2O		
K*VPQVSTPTLVEVSR	K414	HNE, HNE−H_2O, HNE−2H_2O	O	
YTKK*VPQVSTPTLVEVSR	K414	HNE−H_2O		
NLGK*VGSK	K432	HNE, HNE−2H_2O	O	Δ
NLGK*VGSK*CCK	K432/K436	HNE/HNE−2H_2O		
VGSK*CCK	K436	HNE, HNE−2H_2O		
VGSK*CCKHPEAK	K436	HNE, HNE−2H_2O		
VGSK*CCKH*PEAK	K436/H440	HNE−2H_2O/HNE, HNE−H_2O		
VGSKCCK*HPEAK	K439	HNE−H_2O		
CCKH*PEAK	H440	HNE−H_2O		
MPCAEDYLSVVLNQLCVLH*EK	H464	HNE		
MPCAEDYLSVVLNQLCVLH*EKTPVSDR	H464	HNE		
RMPCAEDYLSVVLNQLCVLH*EK	H464	HNE		
VTK*CCTESLVNR	K475	HNE		
RPCFSALEVDETYVPK*EFNAETFTFHADICTLSEK	K500	HNE, HNE−H_2O		
EFNAETFTFH*ADICTLSEK	H510	HNE	O	Δ

S2 Table. Continued.

Peptide Sequence	Modification		HNE 30 μM	HNE 3 μM
	Site	Type		
EFNAETFTFH*ADICTLSEKER	H510	HNE, HNE−H_2O		
RPCFSALEVDETYVPKEFNAETFTFH*ADICTLSEKER	H510	HNE		
EFNAETFTFHADICTLSEK*ER	K519	HNE−$2H_2O$		
RPCFSALEVDETYVPKEFNAETFTFHADICTLSEK*ER	K519	HNE−H_2O		
K*QTALVELVK	K525	HNE, HNE−H_2O, HNE−$2H_2O$	O	Δ
QIKK*QTALVELVK	K525	HNE−H_2O	O	Δ
K*QTALVELVK*HKPK	K525/K534	HNE, HNE−$2H_2O$ /HNE−H_2O		
K*QTALVELVKHK*PK	K525/K536	HNE−H_2O/HNE−H_2O	O	
QTALVELVKHK*PK	K536	HNE−H_2O	O	Δ
ATK*EQLK	K541	HNE−$2H_2O$		
ATKEQLK*AVMDDFAAFVEK	K545	HNE−H_2O		
EQLK*AVMDDFAAFVEK	K545	HNE, HNE−H_2O, HNE−$2H_2O$		
AVMDDFAAFVEK*CCK	K557	HNE−$2H_2O$		
ADDK*ETCFAEEGKK	K564	HNE		
ADDKETCFAEEGK*K	K573	HNE−$2H_2O$		
K*LVAASQAALGL	K574	HNE, HNE−$2H_2O$		

* indicates a modification site.

O indicates a peptide identified automatically.

Δ indicates a peptide identified manually.
