# Supplementary material for: Inhibition effect of pyridoxamine on lipid hydroperoxide-derived modifications to human serum albumin
Source: PLoS One. 2018 Apr 19;13(4):e0196050. doi: 10.1371/journal.pone.0196050 (PMC5908094; doi:10.1371/journal.pone.0196050)
Supplement: S3 Table — * indicates a modification site. (PDF) [file pone.0196050.s014.pdf]

S3 Table. List of ONE-, HNE-, and HPNE-modified HSA peptides identified from the reaction of HSA with 13-HPODE in the presence of AscA.

Peptide Sequence	Modification	
	Site	Type
DAH*KSEVAHR	H3	HPNE
SEVAH*R	H9	HPNE
FK*DLGEENFK	K12	HPNE
FKDLGEENFK*ALVLIAFAQYLQQCPFEDHVK	K20	HPNE
ALVLIAFAQYLQQCPFEDH*VK	H39	ONE, HNE
SLH*TLFGDK	H67	HNE, HPNE
SLH*TLFGDKLCTVATLR	H67	HNE, HPNE
SLHTLFGDK*LCTVATLR	K73	ONE, ONE−H_2O
NECFLQH*KDDNPNLPR	H105	HPNE
QEPERNECFLQH*KDDNPNLPR	H105	HPNE
LVRPEVDVMCTAFH*DNEETFLKK	H128	HPNE
LVRPEVDVMCTAFHDNEETFLK*K	K136	ONE−H_2O
K*YLYEIAR	K137	ONE−H_2O, HPNE
R*H*PYFYAPELLFFAK	R145/H146	ONE/HNE, HPNE
H*PYFYAPELLFFAK	H146	HNE, HPNE
RH*PYFYAPELLFFAK	H146	ONE, HNE, HPNE
RHPYFYAPELLFFAK*R	K159	HPNE
RYK*AAFTECCQAADK	K162	ONE, ONE−H_2O
YK*AAFTECCQAADK	K162	ONE, ONE−H_2O, HPNE
LDELRDEGK*ASSAK	K190	ONE−H_2O, HNE−2H_2O
LK*CASLQK	K199	HPNE
CASLQK*FGER	K205	ONE−H_2O, HNE−2H_2O
DLGEENFK*ALVLIAFAQYLQQCPFEDHVK	K205	HPNE
AFK*AWAVAR	K212	ONE−H_2O
VH*TECCHGDLLECADDR	H242	HNE, HPNE
VH*TECCHGDLLECADDRADLAK	H242	HNE, HPNE
LVTDLTKVH*TECCHGDLLECADDR	H242	HPNE
LVTDLTKVH*TECCHGDLLECADDRADLAK	H242	HPNE
VH*TECCH*GDLLECADDR	H242/H247	HNE/ONE, HNE, HPNE
VH*TECCH*GDLLECADDRADLAK	H242/H247	HNE, HNE−H_2O, HPNE/HNE, HPNE
SH*CIAEVENDEMPADLPSLAADFVESK	H288	HNE, HPNE
CCAAADPH*ECYAK	H367	HPNE
R*H*PDYSVVLLLR	R337/H338	ONE/HNE, HPNE
RH*PDYSVVLLLR	H338	HNE, HPNE
VFDEFK*PLVEEPQNLIK	K378	ONE
K*VPQVSTPTLVEVSR	K414	HPNE
NLGK*VGSK	K432	ONE, HPNE
EFNAETFTFH*ADICTLSEK	H510	HNE, HPNE
EFNAETFTFHADICTLSEK*ER	K519	HPNE
K*QTALVELVK	K525	ONE−H_2O

* indicates a modification site.
